# High-fat diet, microbiome-gut-brain axis signaling, and anxiety-like behavior in male rats

**DOI:** 10.1186/s40659-024-00505-1

**Published:** 2024-05-06

**Authors:** Sylvana I. S. Rendeiro de Noronha, Lauro Angelo Gonçalves de Moraes, James E. Hassell, Christopher E. Stamper, Mathew R. Arnold, Jared D. Heinze, Christine L. Foxx, Margaret M. Lieb, Kristin E. Cler, Bree L. Karns, Sophia Jaekel, Kelsey M. Loupy, Fernanda C. S. Silva, Deoclécio Alves Chianca-Jr., Christopher A. Lowry, Rodrigo Cunha de Menezes

**Affiliations:** 1 Department of Biological Sciences, Laboratory of Cardiovascular Physiology, Federal University of Ouro Preto, Ouro Preto, MG 35400-000 Brazil; 2 Department of Integrative Physiology, University of Colorado Boulder, Boulder, CO 80309 USA; 3 Computing Department, Federal University of Ouro Preto, Ouro Preto, MG 35400-000 Brazil; 4 Department of Psychology and Neuroscience, University of Colorado Boulder, Boulder, CO 80309 USA; 5 Center for Neuroscience, University of Colorado Boulder, Boulder, CO 80309 USA; 6 Department of Biological Science Laboratory of Cardiovascular Physiology, Campus Morro do Cruzeiro s/n, Ouro Preto, 35400-000 MG Brazil

**Keywords:** Anxiety, Dorsal raphe nucleus, High-fat diet, Microbiome, Microbiome-gut-brain axis, Obesity, Raphe nuclei, Serotonergic system, Serotonin, *tph2*

## Abstract

Obesity, associated with the intake of a high-fat diet (HFD), and anxiety are common among those living in modern urban societies. Recent studies suggest a role of microbiome-gut-brain axis signaling, including a role for brain serotonergic systems in the relationship between HFD and anxiety. Evidence suggests the gut microbiome and the serotonergic brain system together may play an important role in this response. Here we conducted a nine-week HFD protocol in male rats, followed by an analysis of the gut microbiome diversity and community composition, brainstem serotonergic gene expression (*tph2*, *htr1a*, and *slc6a4*), and anxiety-related defensive behavioral responses. We show that HFD intake decreased alpha diversity and altered the community composition of the gut microbiome in association with obesity, increased brainstem *tph2*, *htr1a* and *slc6a4* mRNA expression, including in the caudal part of the dorsomedial dorsal raphe nucleus (cDRD), a subregion previously associated with stress- and anxiety-related behavioral responses, and, finally, increased anxiety-related defensive behavioral responses. The HFD increased the Firmicutes/Bacteroidetes ratio relative to control diet, as well as higher relative abundances of *Blautia*, and decreases in *Prevotella*. We found that *tph2*, *htr1a* and *slc6a4* mRNA expression were increased in subregions of the dorsal raphe nucleus in the HFD, relative to control diet. Specific bacterial taxa were associated with increased serotonergic gene expression in the cDRD. Thus, we propose that HFD-induced obesity is associated with altered microbiome-gut-serotonergic brain axis signaling, leading to increased anxiety-related defensive behavioral responses in rats.

**Supplementary Information:**

The online version contains supplementary material available at 10.1186/s40659-024-00505-1.

## Introduction

Obesity and anxiety disorders are often comorbid [1] and are increasing in modern urban societies [2, 3]. Inflammatory signaling is thought to be important in this comorbidity [1]. In animal models, high-fat diet (HFD)-induced obesity increases neuroinflammation and anxiety-related defensive behavioral responses [4–6]. For instance, we have recently shown that rats given a HFD respond with increased neuroinflammation in brain regions that control anxiety-related defensive behavioral responses [6]. Although the mechanism involved in the effects of HFD on neuroinflammation and anxiety-related defensive behavioral responses are not fully understood, altered microbiome-gut-brain (MGB) axis signaling is thought to play an important role [7, 8]. HFD-induced changes in the diversity and community composition of the gut microbiome are fundamental in determining health and disease through changes in innate immunity, inflammation, and cognitive function [9, 10]. Gut dysbiosis – a condition where the microbiome presents an unhealthy imbalance in bacterial diversity and community composition – induces inflammation and neuroinflammation, alters brain serotonergic signaling, and increases anxiety-like defensive behavioral responses [4, 8, 9, 12].

The brain’s serotonergic system plays an important role in regulating emotional behavior, including anxiety-like defensive behavioral responses [13–15]. The dorsal raphe nucleus (DR) is the main source of serotonin in the brain [16], and serotonergic projections arising from the DR modulate different aspects of emotional behavior and cognition [17]. Specifically, the activation of serotonergic neurons in the dorsal part of the dorsal raphe nucleus (DRD), and the caudal part of the dorsal raphe nucleus (DRC) facilitate anxiety-like responses [18]. The caudal aspect of the DRD nucleus (cDRD), also referred to as the dorsomedial DR (for a description of nomenclature, see Table 4 in [13], which has strong projections to forebrain systems controlling emotional behavior, is believed to be a key player in controlling anxiety-related defensive behavioral responses in rodents [13, 14, 19, 20] and displays increased expression of *TPH2* mRNA, encoding the neuronal isoform of tryptophan hydroxylase, TPH2, in persons with depression who died by suicide [21].

Previous studies have shown that a HFD can influence serotonergic signaling. For instance, male rats fed a HFD for 12 weeks had higher levels of serotonin (5-hydroxytryptamine; 5-HT) in the hippocampus [5]. However, the mechanisms underlying the effects of HFD on serotonergic signaling are not well understood. Interestingly, *tph2*, which encodes tryptophan hydroxylase 2, the rate-limiting enzyme for the synthesis of 5-HT in the central nervous system of rodents, impacts emotional behavior [22, 23]. Likewise, other genes related to 5-HT signaling, such as *htr1a* and *slc6a4*, have also been linked to anxiety-related defensive behavioral responses in rodents [24–26]. The *HTR1A* gene, which encodes the 5-hydroxytryptamine receptor 1 A, an autoreceptor that controls serotonergic neuronal firing rates and thus the synthesis and release of serotonin, has been associated with generalized anxiety disorder and panic disorder [27, 28]. The *SLC6A4* gene, which encodes the sodium dependent, high-affinity, low-capacity serotonin transporter, regulates extracellular and synaptic serotonin concentrations, and has been associated with anxiety-related temperamental dimensions [29], and depression [30].

Previous studies suggest that animals fed a HFD have a higher risk of developing anxiety-related defensive behaviors, but the underlying mechanisms remain unknown. Given the aforementioned knowledge gap, we hypothesized that a HFD alters the gut microbiome diversity and community composition, increases the serotonergic gene expression in the cDRD, and induces anxiety-related defensive behavioral responses in male Wistar rats. Thus, in the present study, we investigated the effects of a HFD treatment for 9 weeks on the gut microbiome, expression of *tph2*, *htr1a* and *slc6a4* genes in the brainstem raphe nuclei, and anxiety-related defensive behavioral responses in adult male Wistar rats.

## Materials and methods

### Animals and ethics statement

Male Wistar rats weighing 100 ± 10 g (5–6 weeks old) were provided by the Center of Animal Science from the Federal University of Ouro Preto (CCA/UFOP) and housed in groups of four in acrylic cages (34 cm width x 41 cm length x 17 cm height) and kept in the maintenance animal facility annex to the Cardiovascular Physiology Laboratory for 9 weeks. Adolescence in rats consists of early adolescence [prepubescent or juvenile, postnatal day (pnd) 21–34], middle adolescence (periadolescent, pnd 35–46), and late adolescence (pnd 47–59) time periods [31]. Since rats were subjected to the protocol around five weeks of age, they were in the middle-adolescence period (pnd 35–42) upon arrival, and adulthood (pnd ≥ 91–98) at the time of behavioral testing [31]. During the experiment, animals were kept in the maintenance facility annex to the Cardiovascular Physiology Laboratory. Animals were kept under a light/dark cycle of 12:12 h (lights on at 6 am), controlled temperature (23 ± 1 ºC), and controlled noise (60–80 dB) with free access to food and tap water. All procedures were approved by the Institutional Animal Care and Use Committee (CEUA#2015/16) and performed according to the regulations set forth by the National Institutes of Health *Guide for the Care and Use of Laboratory Animals*, and the National Council for the Control of Animal Experimentation (CONCEA).

### Experimental design

**Fig. 1:**
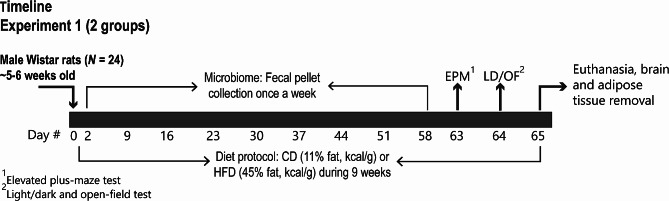
Experimental timeline. Beginning at about 5 or 6 weeks of age (postnatal day (pnd) 35–42; post-weaning period; 100 g ± 10 g), adolescent male rats were housed in groups of 4 per cage. Animals were fed either a standard control diet (CD) or a 45% high-fat diet (HFD), for 9 weeks, until the young adult age (pnd 98–105). Fecal stool samples were collected from both CD and HFD animals, once a week during the nine weeks of the diet protocol, for analysis of the fecal microbiome. Rats were subjected to the elevated plus-maze (EPM) test on day 63 and the light/dark box (LDB) test on day 64 followed by the open-field (OF) test to assess anxiety-like behavior. Twenty-four hours following the behavioral tests, rats were euthanized, and their fat pad tissues (epididymal, inguinal, and retroperitoneal) were removed for determination of the adiposity index (AI). Further, brains were removed, and flash frozen in liquid isopentane, wrapped in aluminum foil and stored at − 80 ºC in preparation for in situ hybridization histochemistry for analysis of *tph2*, *htr1a*, and *slc6a4* mRNA expression in the brainstem raphe nuclei. Abbreviations: AI, adiposity index; CD, control diet; EPM, elevated plus-maze; HFD, high-fat diet; LDB, light/dark box; OF, open-field

The effects of HFD feeding on gut microbiome diversity and community composition, central serotonergic systems, and anxiety-like defensive behavioral responses in the EPM, LDB and OF tests were assessed. Rats fed with a control diet (CD; *N* = 12) or HFD (*N* = 12) were group housed in groups of four according to their diet treatment for 9 weeks. During this period, fecal pellets were collected once a week, for later microbiome analysis. The first day of the diet protocol was considered day 0. On day 63 (1st day of behavioral testing), animals were transferred to the experimental room, 30 min before the beginning of the behavioral testing, under controlled temperature (23 ± 1 ºC), noise (60–80 dB), and luminosity (∼ 60 lx). All behavioral tests took place between 9:00 am and 5:00 pm. Animals were placed on the EPM facing one of the enclosed arms of the apparatus, and allowed to freely explore the center and the four arms of the apparatus for 300 s. Both groups (i.e., CD and HFD) were tested in the same period of the day, alternating between CD and HFD cages. After behavioral testing on the EPM, rats were returned to the animal maintenance facility. On day 64 (2nd day of behavioral testing), animals were transferred to the same experimental room, under the same experimental conditions. Animals were placed in the dark part of the LD apparatus for 30 s with the door closed, and then, as previously described [32], allowed to freely explore the testing environment for 300 s. Immediately following this test, animals were removed from the LDB and put in the center area of the OF arena (60–70 lx) and allowed to freely explore for 300 s. At the end of the experimental procedures, rats were regrouped in their original home cage and returned to the animal maintenance facility. All tests were video recorded for later behavioral analyses. On day 65, all animals were euthanized using rapid decapitation, and their brains were removed for in situ hybridization histochemistry for analysis of *tph2, htr1a*, and *slc6a4* mRNA expression in the dorsal raphe nucleus (DR), median raphe nucleus (MnR), pontomesencephalic reticular formation (PMRF), and B9 serotonergic cell group. Adipose tissue was removed for confirmation of the effects of HFD on adiposity (see Fig. 1 for details).

### Diet

Male Wistar rats were randomly divided into two groups based on diet protocol: (1) fed with a standard control diet (CD; NuviLab^®^; Table S1) with 11% kcal/g fat; or (2) fed with a high-fat diet (HFD; PragSoluções Biociências, Comércio e Serviços Ltda; Table S1) with 45% kcal/g fat, as previously described [4, 6]. Changes in body composition induced by HFD feeding were evaluated for final body weight, body weight gain, and adiposity index (AI; calculated by the sum of epididymal, retroperitoneal, and inguinal visceral white fat pad weight divided by final rat weight x 100), as shown in Table S2.

### Behavioral apparatus

To investigate anxiety-related defensive behavioral responses in rodents we applied a sequence of three behavioral tests commonly used in this context: the elevated plus-maze (EPM), the light/dark box (LDB), and the open-field (OF). The EPM (Insight®, Ribeirão Preto, Brasil) was made of wood with four arms of equal dimensions (50 cm length x 12 cm width), wherein, two arms were enclosed by 40-cm high walls, and two arms were open and only surrounded by a 1 cm tall Plexiglas® (polymethyl methacrylate) rim to avoid falls. The maze was elevated 50 cm above the floor. Rats were placed in the central area of the EPM, facing one of the enclosed arms, and the time spent exploring the open arm or the enclosed arm, as well as the number of entries on each arm, was recorded for behavioral assessment.

The LDB was made of acrylic and had two compartments of equal dimensions (24 cm width x 24 cm length x 30 cm height) connected by a small opening at the floor level (11 cm x 11 cm) to allow animals to cross into either side. One of the compartments was made with a transparent acrylic, and the other made with a black mat acrylic. Animals were tested in the LDB as previously described [32]. The time spent by each animal in the light or dark compartment (light part with ∼ 60 lx) and the number of entries into the light compartment were recorded and analyzed.

The OF square arena (tested under ∼ 60 lx illumination) was made from black mat acrylic (60 cm width x 60 cm length x 40 cm height) and had its floor divided into sixteen squares in a 4 × 4 grid, modified from [33]. Three behavioral parameters, besides the locomotor activity [6], were observed in this test [34]: (1) time spent in the center area (i.e., the four squares in the center of the arena); (2) time spent in the outer area (i.e., the twelve squares adjacent to the walls of the arena); and (3) the number of rearing events (i.e., vertical exploration). Animals were placed in the center area of the OF and tested for 300 s. All apparatus were cleaned before each exposure with a 20% ethanol solution.

### Fecal collection and microbiome analysis by 16S rRNA gene sequencing

During the 9 weeks of diet protocol (i.e., CD or HFD), once a week (i.e., 9 samples total), each rat was individually transferred to a new cage (cleaned with 70% ethanol) layered with sterilized bedding to have their fecal stools collected. Two fecal pellets were collected from each animal in a 1 h interval, just before the beginning of the active (dark) phase of the daily light: dark cycle. All samples were collected in RNase-free 2 mL Eppendorf tubes and immediately frozen in the − 80 ºC freezer until further processing.

DNA was extracted using the PowerSoil DNA extraction kit (Cat No. 12888-100 & 12955-4, MoBio/Qiagen Laboratories, Carlsbad, CA, USA) according to the manufacturer’s instructions. 16S rRNA gene sequences in isolated DNA were PCR-amplified using HotStarTaq Master Mix (Cat. No. 203433, Qiagen, Valencia, CA, USA) and 515 F (5’-GTGCCAGCMGCCGCGGTAA-3’), 806 R (5’-GGACTACHVGGGTWTCTAAT-3’) primer pair (Integrated DNA Technologies, Coralville, IA, USA) targeting the V4 hypervariable region of the 16S rRNA gene modified with a unique 12-base sequence identifier for each sample and the Illumina adapter as previously described in [35]. The thermocycling program consisted of an initial step at 94 °C for 3 min followed by 35 cycles (94 °C for 45 s, 55 °C for 1 min, and 72 °C for 1.5 min), and a final extension at 72 °C for 10 min. PCR reactions were run in duplicate and the products from the duplicate reactions were pooled and visualized on an agarose gel to ensure successful amplification. PCR products were cleaned and normalized using a SequalPrep Normalization Kit (Cat. No. A1051001, Thermo Fisher, Waltham, MA, USA) following manufacturer’s instructions. The normalized amplicon pool was sequenced on an Illumina MiSeq run by using V3 chemistry and 600 cycle, 2 × 300-bp paired-end sequencing. All sequencing and library preparation were conducted at the University of Colorado Boulder BioFrontiers Next-Gen Sequencing core facility.

### In situ hybridization histochemistry

Previously published methods were used for in situ hybridization histochemistry [13, 36]. Briefly, brains were sectioned into 12 μm-thick sections on a cryostat (Leica CM 1950, Leica Biosystems, Buffalo Grove, IL, USA), in a series of 7 sets of sections, thaw mounted on Histobond® slides (Cat. No. 16,004-406; VWR, West Chester, PA, USA) and stored at − 80 °C. [^35^S]-UTP-labeled riboprobes directed against *tph2* mRNA were generated using standard transcription methods, as described previously [36]. The *tph2* mRNA was detected using a 462 base (1552–2013) antisense riboprobe (kindly provided by Dr. Stanley J. Watson, re-subcloned by Dr. Heidi Day) complementary to the rat cDNA encoding Tph2 (i.e., *tph2*, NCBI Reference Sequence: NC_005106.4). To obtain an appropriate template for riboprobe transcription, a HindIII fragment was removed, leaving a 461 bp fragment of rat *tph2*, containing 23 bp of coding sequence plus 438 bp of 3 = UTR (corresponding to sequence numbers 1552–2013) in the parent vector, pT7T3D-PacI. The resulting construct was linearized with HindIII and transcribed with T3 RNA polymerase (Promega, Madison, WI, USA) to generate a specific *tph2* antisense riboprobe. The control sense probe was created by linearizing the same construct with EcoRI, and transcribing with T7 RNA polymerase [15].

Previously published methods were used for in situ hybridization histochemistry using oligonucleotide probes [37, 13. To detect *htr1a* mRNA, two synthetic anti-sense oligonucleotides, one 49-base oligonucleotide (5′-ACG AAG TTC CTA AGC TGG TGC CTG CTC CCT TCT TTT CCA CCT TCC TGA C-3′, Integrated DNA Technologies, Coralville, IA, USA) complementary to bases 810–858 of rat *htr1a* mRNA and one 47-base oligonucleotide (5′-GCC TCA CTG CCC CAT TAG TGC ACG GAG TCC CCA CCG CCC TGT TCT CA-3′, Integrated DNA Technologies) complementary to bases 923–969 of rat *htr1a* mRNA, were used [37.

To detect *slc6a4* mRNA, a synthetic 50-base antisense oligonucleotide (5′-ACT GCA GAG TAC CCA TTG GAT ATT TGG CTA GGC TCT GCC CTG TCC GCT GT-3′, Integrated DNA Technologies) based on the published sequence of the rat serotonin transporter cDNA clone [38] of *slc6a4* mRNA (*Rattus norvegicus* solute carrier family 6 (neurotransmitter transporter, serotonin), member 4, mRNA; GenBank® Accession no., NM_013034.3) was used as previously described [13, 15]. The oligonucleotides were labeled at the 3’end with [^35^S]-deoxyadenosine-5′-triphosphate (Cat. No. 5,620,001, MP Biomedicals, Santa Ana, CA, USA) using terminal deoxynucleotidyl transferase (20 U/µL, Cat. No. EP0161, Fermentas, Glen Burnie, MD, USA) for 1 h at 37 °C, cleaned using a QIAquick® nucleotide removal kit (Cat. No. 28,304, Qiagen, Valencia, CA, USA), and used in an in situ hybridization histochemistry assay as described previously [15, 37].

For riboprobe-based detection of *tph2* mRNA expression, sections were fixed in 4% paraformaldehyde for 1 h, acetylated in 0.1 M triethanolamine hydrochloride with 0.25% acetic anhydride for 10 min, and dehydrated through graded alcohols. Sections were hybridized overnight at 55 °C with a [^35^S]-UTP-labeled riboprobe diluted in hybridization buffer containing 50% formamide, 10% dextran sulfate, 2× saline sodium citrate (SSC), 50 mM PBS, pH 7.4, 1× Denhardt’s solution, and 0.1 mg/ml yeast tRNA. The following day, sections were treated with RNase A, 200 µg/ml at 37 °C for 1 h and washed to a final stringency of 0.1× SSC at 65 °C (1 h). Dehydrated sections were exposed to x-ray film (BioMax MR; Eastman Kodak, Rochester, NY, USA) for region- and probe-appropriate times (1–3 weeks) prior to film development. For oligonucleotide-based detection of *htr1a* and *slc6a4* mNA expression, we used an in situ hybridization histochemistry assay as described previously [37].

Autoradiographic images of the probe bound to *tph2* mRNA, encoding the rate-limiting enzyme in the synthesis of brain serotonin; *htr1a* mRNA, encoding the 5-HT_1A_ inhibitory auto-receptor and *slc6a4* mRNA, encoding the sodium dependent, high-affinity, low-capacity serotonin transporter, together with ^14^C-labeled standards, were measured using a computer‐assisted image analysis system. For each gene, all slides from the study were apposed to the same film, allowing us to use a single set of ^14^C‐labeled standards for reference per gene. Analysis was performed on a PC using the publicly available NIH‐developed image analysis software ImageJ (https://imagej.nih.gov/ij/). Virtual matrices in the shape of the respective DR subregions were created, overlaid with the image, and the “mean gray value x area”, taking into account only the area of above-threshold signal, within each matrix, was measured. All measurements were taken while blinded to the treatment groups. During the entire analysis, a constant threshold function was applied, which determined the area that was measured within each matrix. Thus, all pixels with a gray density below threshold were automatically excluded. In addition, the individual background of each image, taken from the adjacent periaqueductal gray region, was measured, and subtracted from each value. An atlas for analysis of *tph2, htr1a*, and *slc6a4* mRNA expression across the rostrocaudal extent of the DR was created by comparing the image of the tissue sections with a stereotaxic rat brain atlas [39] and with *tph2* mRNA expression topography as reported by Gardner et al. [15]. According to Gardner et al. [15] for Tph immunostaining [40], each rostrocaudal level was further divided into respective subregions of the DR. Throughout all rostrocaudal levels, the values for each subdivision were then averaged to obtain mean mRNA expression values in each subregion, and all values in all rostrocaudal levels and subregions were calculated in each animal to evaluate the effects of treatment on the overall levels of mRNA expression in the DR, MnR, PMRF, and B9 serotonergic cell group. A total of 16 rostrocaudal levels were studied throughout the brainstem (see Fig. S1). The subdivisions of the DR studied were defined as follows: dorsal raphe nucleus, caudal part (DRC), − 8.336 mm to − 8.672 mm from bregma; dorsal raphe nucleus, dorsal part (DRD), − 7.412 mm to − 8.252 mm from bregma; dorsal raphe nucleus, interfascicular part (DRI), − 8.336 mm to − 8.672 mm from bregma; dorsal raphe nucleus, ventral part (DRV), − 7.412 mm to − 8.420 mm from bregma; left and right dorsal raphe nucleus, ventrolateral part/ventrolateral periaqueductal gray region (left and right DRVL/VLPAG), − 7.748 mm to − 8.420 mm from bregma; left and right supralemniscal nucleus (B9), − 7.412 mm to − 8.168 mm from bregma; median raphe nucleus (MnR), − 7.412 mm to − 8.672 mm from bregma; pontomesencephalic reticular formation (PMRF) – 7.412 mm to – 8.084 mm from bregma.

### Statistical analysis

For analysis of EPM, LDB, and OF test behavioral data, comparisons of two independent samples were made using Student’s *t*-tests. To test normality of the data, we considered the Kolmogorov-Smirnov test for *p >* 0.05. For statistical comparisons, GraphPad Prism software (version 8.01, GraphPad Inc., La Jolla, CA, USA) was used.

For analysis of in situ hybridization histochemistry data we used the software package IBM SPSS Statistics (versions 22.0 and 24.0, SPSS Inc., Chicago, IL, USA). Extreme statistical outliers were identified using Grubbs’ test for single extreme outliers using two-sided α = 0.05 [41] and were removed from the analysis. In situ hybridization histochemistry analyses were made through a survey of linear mixed models (LMM) with different covariance structures performed and the model with the best − 2 log-likelihood value, an information criterion function used for goodness of fit, was selected. For analysis of in situ hybridization histochemistry data, mean gray values x area for each DR subdivision at each rostrocaudal level of the DR in each treatment group were generated. The model with the best − 2 log-likelihood value was selected for each gene, *tph2*, *htr1a*, and *slc6a4* with diet, rostrocaudal level, and DR subregion as fixed effects, and rostrocaudal level as the repeated effect. Additional linear mixed models were run, and the best covariance structure was selected using the best − 2 log-likelihood value for each individual subregion of the DR, with diet and rostrocaudal level as fixed effects and rostrocaudal level as a repeated effect. Post hoc pairwise comparisons were made with Fisher’s least significant difference (LSD) test. Furthermore, in the in situ hybridization histochemistry analysis no post hoc analyses were conducted at specific points in the rostrocaudal extent of the DR, MnR, PMRF, or B9 if one of the group sample sizes was below 50% of the full sample size for that treatment group. Additionally, post hoc analyses were conducted only when overall and secondary linear mixed models yielded significant effects of diet or interactions between diet and rostrocaudal level within raphe subregion or rostrocaudal level. Data are presented as means ± standard errors of means (SEMs). Two-tailed significance was set at *p* < 0.05.

For analysis of the effects of HFD or CD on microbiome diversity and community structure, raw sequences were trimmed, demultiplexed, merged, quality filtered (maxee value of 1 and singletons removed), and clustered into greater than or equal to 97% similar phylotypes using QIIME 2 UPARSE 8 [42]. Quality reports from the fastq_eestats2 command were used to determine the fixed length (197 nucleotides for the forward read and 151 nucleotides for the reverse read) at which the raw sequences were trimmed as suggested by the developers. Merging criteria were adjusted according to developer guidelines found on the UPARSE website for merging reads with long overlap. Taxonomy was assigned using the Ribosomal Database Project classifier [43] trained on the Greengenes 13_8 16S rRNA gene database [44]. For downstream diet life stage analysis, samples were rarefied to a depth of 2500.

Quantitative Insights into Microbial Ecology (QIIME 2 2020.2) was used to generate core-metrics results for alpha and beta diversity, and biplot distance, to examine the microbial community structure [35, 45]. Relative abundance was analyzed using the mctools package (https://github.com/leffj/mctoolsr/*)* in the open-source tools for R (RStudio, Inc, version 1.2.1578, Boston, MA, USA). Analysis of Composition of Microbiomes (ANCOM v2.1; https://github.com/FrederickHuangLin/ANCOM*)* package was used to analyze relative abundance composition.

Pearson’s or Spearman’s correlation regression, heatmap correlations, Firmicutes/Bacteroidetes ratio, gene expression x relative abundance of specific taxa (transformed from arcsin sqrt root), and time in the open arm x relative abundance of specific taxa were analyzed and constructed using Jupyter notebook (code available at https://github.com/bioinfonupeb/hfd-insitu-microbiome), and plotted using the GraphPad Prism 8.0.

## Results

### Control diet (CD) and high-fat diet (HFD) differentially shaped gut microbiota composition along nine weeks of diet protocol

**Fig. 2:**
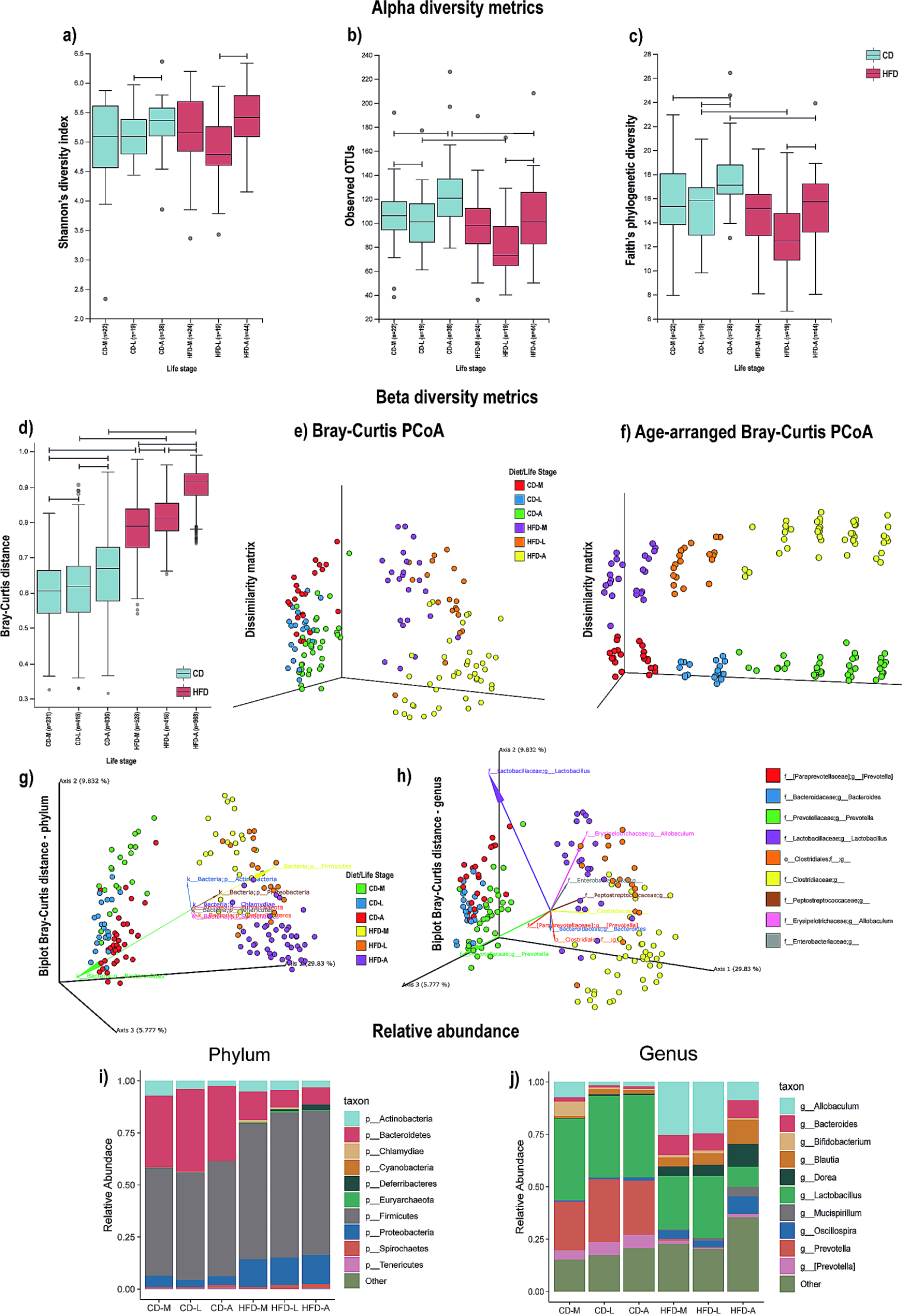
Effects of high-fat diet (HFD) on alpha diversity, beta diversity, and community composition of the gut microbiome across mid-adolescence, late adolescence, and adulthood. Alpha diversity as measured by (**a**) Shannon’s diversity index, (**b**) Observed OTUs, (**c**) Faith’s phylogenetic diversity. (**d**) Beta diversity distance comparison plot with box plots illustrating distances within and between a single treatment group using Bray-Curtis distance, (**e**) Bray-Curtis principal coordinates (PCoA) plot of dissimilarity matrix (phylum level), and (**f**) age-arranged Bray-Curtis PCoA plot of dissimilarity matrix (phylum level). Biplot using Bray-Curtis distance for (**g**) phylum and (**h**) genus, illustrating microbial community analysis and composition (vectors) using principal component analysis of center log ratio transformed and standardized data from CD and HFD groups. Vectors point in the direction of the greatest increase of values for corresponding phylum and genus. Top ten taxa with highest relative abundances, illustrated by stacked vertical bar charts for (**i**) phylum and (**j**) genus. Data are expressed as means ± SEMs, *p* < 0.05; Kruskal–Wallis *H* test and PERMANOVA pairwise test. Abbreviations: CD-A, control diet group/adulthood; CD-L, control diet group/late adolescence; CD-M, control diet group/middle adolescence; HFD-A, high-fat diet group/adulthood; HFD-L, high-fat diet group/late adolescence; HF D-M, high-fat diet group/middle adolescence; pnd, postnatal day

HFD for 9 weeks induced obesity in rats (Table S2) and impacted the gut microbiota alpha diversity and community composition. Alpha diversity was analyzed at three life-stages during each diet condition, i.e., middle adolescence (compiled for samples collected at 5–6 weeks of age for CD-M and HFD-M), late adolescence (compiled for samples collected at 7–8 weeks of age CD-L and HFD-L) and adult (compiled for samples collected at 9–13 weeks for CD-A and HFD-A). The HFD-A and the CD-A had higher alpha diversity during this phase than during late adolescence (HFD-L and CD-L; Fig. 2a), based on the Shannon’s diversity index (Kruskal–Wallis *H* test= 13.59; *p* < 0.018). The observed OTUs (Fig. 2b) were consistent with the analysis, demonstrating that the gut microbiome is more diverse in adulthood than in earlier stages, regardless of the diet. Analysis of Observed OTUs also showed that the CD-A microbiome was more diverse compared to the HFD-A microbiome (Fig. 2b). In order to confirm these data we analyzed the alpha diversity metrics using Faith’s phylogenetic diversity, which measures the total length of branches in a reference phylogenetic tree for all species in a given sample [46]. These analyses confirmed that animals had a higher alpha diversity during adulthood and that the HFD reduced diversity when compared to the control diet (Kruskal–Wallis *H* test = 34.12; *p <* 0.001; Fig. 2c). Overall, these data indicate increasing diversity of the gut microbiome across developmental stages and indicates that the HFD reduces the microbiome diversity at the adult stage.

Analysis of beta diversity revealed clear differences between HFD- and CD-fed groups, as showed by Bray-Curtis distances at the phylum level, PCoA at the phylum level, and age-arranged Bray-Curtis dissimilarity matrix, also at the phylum level (Fig. 2d–f). Bray-Curtis distances analysis (PERMANOVA; *pseudoF =* 20.241; *p <* 0.001) pointed to a strong difference between all life stages of rats fed a HFD compared to a CD (Fig. 2d). Differences in bacterial community dissimilarity were observed between HFD- and CD-fed groups across time (Fig. 2f). Moreover, we also observed changes in the bacterial community dissimilarity throughout development regardless of the diet regimen (Fig. 2d). Weighted and unweighted UniFrac analysis and PCoA matrices pointed in the same direction, reinforcing that dissimilarity of microbial communities was dependent on life stage (i.e., middle adolescence, late adolescence, or adult stage) and dependent on the composition of the diet consumed (i.e., CD or HFD), see supplementary text and (Fig. S2a-f). Further, we visualized the results from combined principal component scores (i.e., diet and life stages) and loading vectors as taxa (illustrated as a biplot using Bray-Curtis distance), providing insight into how specific taxa contributed to the dissimilarity between groups, for phylum (Fig. 2g) and genus levels (Fig. 2h).

Higher relative abundances of the Firmicutes phylum (also referred to as Bacillota) were associated with the HFD group in all life stages (Fig. 2i), while higher relative abundances of the Bacteroidetes phylum were associated with the CD group in all life stages. Furthermore, at the genus level, we observed higher relative abundances of the genera *Lactobacillus* and *Prevotella* (within the Bacteriodetes phylum, also referred to as Bacteroidota) within CD, and higher *Allobaculum, Blautia* and *Dorea* within the HFD group. Further analysis of all taxonomic levels using taxa bar plots is found in the supplementary text (Fig. S2g-j).

**Fig. 3:**
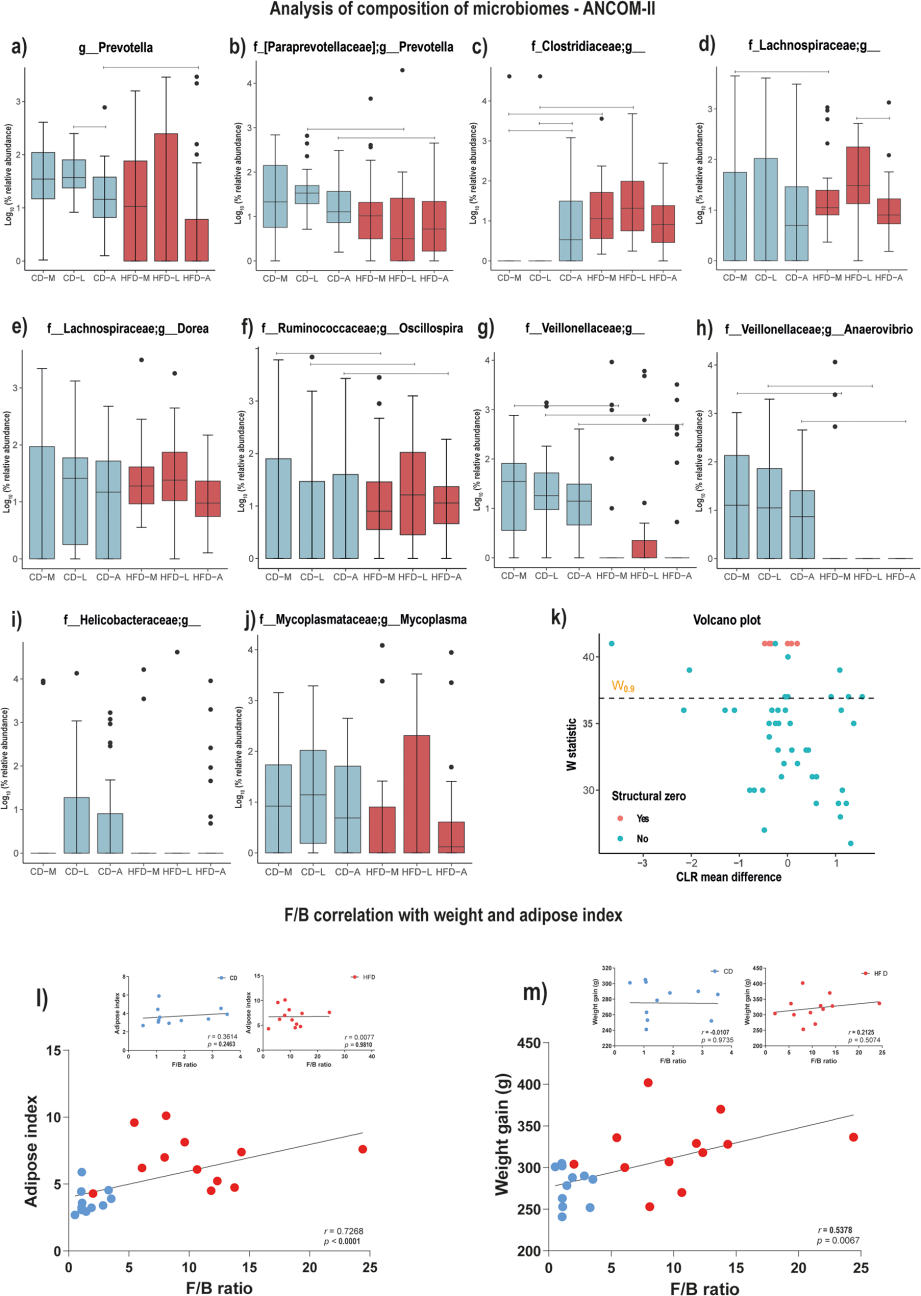
Effects of CD or HFD, and stratified life-stages on gut microbiome community composition. Main effect of diet was analyzed using ANCOM-II (significant at FDR 0.05). Main effects of diet were observed for the following taxa. (**a**) *Prevotella*, (**b**) [f_Paraprevotellaceae];g_*Prevotella*, (**c**) Clostridiaceae;g_, (**d**) Lachnospiraceae;g_, (**e**) f_Lachnospiraceae;g_*Dorea*, (**f**) f_Ruminococcaceae;g_*Oscillospira*, (**g**) f_Veillonellaceae;g_, (**h**) f_Veillonellaceae;g_*Anaerovibrio*, (**i**) f_Helicobacteraceae;g_, (**j**) f_Mycoplasmataceae;g_*Mycoplasma*, and (**k**) Volcano plot. (**l** and **m**) Effect of control diet (CD) and high-fat diet (HFD) on the ratio between the relative abundances of Firmicutes and Bacteroidetes phyla (F/B ratio). (**l**) and (**m**) represent Pearson’s correlation coefficient of F/B ratio versus adiposity measures, and *r* and *p* values are shown on the panels according to the analysis of both CD and HFD groups x F/B ratio, or separately for CD x FB/ratio, and HFD x F/B ratio. Graphs illustrate correlations between the F/B ratio and (**l**) Adipose index and (**m**) total body weight gain. Blue circles represent CD (*n* = 12) group and red circles represent HFD (*n* = 12) group. Abbreviations: CD-A, control diet group/adulthood; CD-L, control diet group/late adolescence; CD-M, control diet group/middle adolescence; HFD-A, high-fat diet group/adulthood; HFD-L, high-fat diet group/late adolescence; HFD-M, high-fat diet group/middle adolescence

As a conservative approach to understanding which taxa were modulated by diet at the genus level [47–49], we performed an ANCOM-II analysis. At this level, relative abundances of multiple *Prevotella* taxa, including *Prevotella* (g_Prevotella; within the Bacteroidetes phylum) and *Prevotella* (f_Paraprevotellaceaea; g_*Prevotella*) were overall lower in HFD-A relative to CD-A (Fig. 3a-b). Further, relative abundances of an unidentified genus in the family Veillonellaceae and genus *Anaerovibrio* (also within the Veillonellaceae family) were lower in HFD compared to the matched CD-life stages (see supplementary text and Fig. 3g-h). Finally, an unidentified genus in the family Clostridiaceae was higher in the HFD-M and HFD-L life stages, compared to matched CD-life stages (see Fig. 3c and supplementary text).

In summary, the main findings were that bacterial communities changed throughout adolescent development and adulthood phases and that consumption of a HFD altered the gut microbiome diversity and community composition. These changes were characterized by lower relative abundances of *Prevotella* and multiple taxa belonging to the *Lactobacillus* genus in animals fed a HFD.

### High-fat diet induced-obesity increased *tph2, htr1a*, and *slc6a4* mRNA expression in brainstem raphe nuclei

#### *tph2* gene expression

**Fig. 4:**
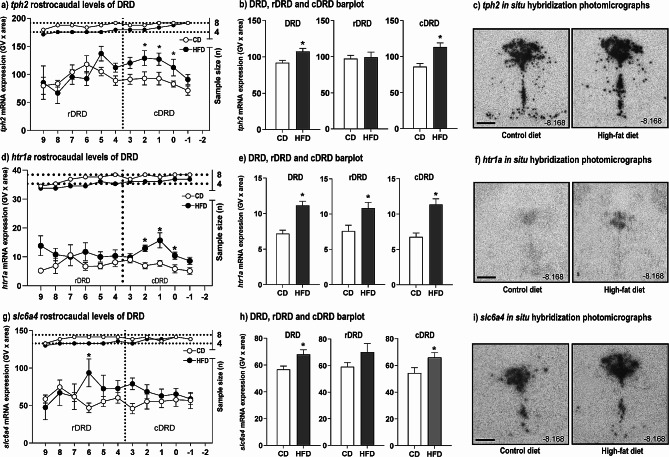
Effects of nine weeks of control diet (CD) or high-fat diet (HFD) protocol on *tph2* mRNA expression in subdivisions of the dorsal raphe nucleus (DRD, rDRD and cDRD). (**a-i**) Graphs illustrate (**a**) *tph2*, (**c**) *htr1a*, and (**e**) *slc6a4* mRNA expression in the dorsal raphe nucleus, dorsal part (DRD), including the rostral (rDRD) and caudal (cDRD) aspects. Expressed as means ± SEMs of gene expression levels at specific rostrocaudal levels or within subregions. (**b, e, h**) Compiled rostrocaudal levels for DRD, rDRD, and cDRD of (**b**) *tph2*, (**e**) *htr1a*, and (**h**) *slc6a4*. (**c, f, i**) Photomicrographs for in situ hybridization histochemistry of (**c**) *tph2*, (**f**) *htr1a*, and (**i**) *slc6a4*. White circles represent CD, and black circles represent HFD group. *p < 0.05 versus CD at the same rostrocaudal level: versus CD. Rostrocaudal levels 9 = − 7.412 mm, 8 = − 7.496 mm, 7 = − 7.580 mm, 6 = − 7.664 mm, 5 = − 7.748 mm, 4 = − 7.832 mm, 3 = − 7.916 mm, 2 = − 8.00 mm, 1 = − 8.084 mm, 0 = − 8.168 mm, − 1 = − 8.252 mm, − 2 = − 8.336 mm, − 3 = − 8.420 mm, − 4 = − 8.504 mm, − 5 = − 8.588 mm, and − 6 = − 8.672 mm. Abbreviations: cDRD, caudal aspect of the dorsal raphe nucleus, dorsal part; DRD, dorsal raphe nucleus, dorsal part;; rDRD, rostral aspect of the dorsal raphe nucleus, dorsal part; CD-A, control diet group/adulthood; CD-L, control diet group/late adolescence; CD-M, control diet group/middle adolescence; HFD-A, high-fat diet group/adulthood; HFD-L, high-fat diet group/late adolescence; HF D-M, high-fat diet group/middle adolescence. Scale bar (**c, f, i**) 1 mm

In situ hybridization histochemistry was used to investigate the effects of HFD intake on serotonergic gene expression, i.e., *tph2, slc6a4* and *htr1a* mRNA, in the DR, MnR, PMRF, and B9 serotonergic cell group. Linear mixed model (LMM) analysis of *tph2* mRNA expression showed a main effect of diet [*F*_(1, 165.3)_ = 19.1; *p* < 0.0001] for compiled DR, MnR, B9 and PMRF structures (Figure S3a). Further, interaction between diet x subregion approached statistical significance [*F*_(9, 98.7)_ = 1.9; *p* = 0.067]. Mean expression of *tph2* mRNA compiled across all rostrocaudal levels of all subregions was increased in HFD group compared to CD group (*p <* 0.0001; Fig. S3i). Based on this finding, secondary LMM analyses were used to determine effects of diet within each subregion. This analysis revealed increased *tph2* mRNA expression in the cDRD [*F*_(9, 2727.1)_ = 4.2; *p* < 0.0001; Fig. 4a, b], a subregion of the DR in which increased *tph2* mRNA expression is associated with increased anxiety-like states [13, 50]. Post hoc analysis using Fisher’s LSD pairwise comparisons showed increased expression of *tph2* in rats in the HFD group at bregma level − 8.000 (*p* = 0.003), − 8.084 (*p* = 0.015), and − 8.168 (*p* = 0.018; Fig. 4a, g). Further, the mean expression of *tph2* compiled for the cDRD showed increases in the HFD group (*p =* 0.0002; Fig. 4b). In the rostral aspect of the dorsal raphe nucleus, dorsal part (rDRD), values approached significance [*F*_(11, 1713.8)_ = 1.9; *p =* 0.05; Fig. 4a], and no difference was shown in the rDRD in the compiled analysis (Fig. 4b).

The LMM analysis considering subregions of the DR also showed increased *tph2* mRNA expression in the caudal aspect of the ventral part of the dorsal raphe nucleus [cDRV; *F*_(13, 3029.8)_ = 8.9; *p* < 0.0001; Fig. S3b], and post hoc pairwise comparisons showed increased *tph2* mRNA expression in the HFD group at bregma − 8.252 (*p* = 0.030), − 8.000 (*p* = 0.004), and − 7.916 (*p* = 0.007). Further, the mean compiled *tph2* mRNA expression in the cDRV was higher in the HFD group, relative to the CD group (*p =* 0.0023; Fig. S3j). No differences were found at specific rostrocaudal levels of the rostral aspect of the ventral part of the DR (rDRV; Fig. S3b), but there was an increase in the mean compiled *tph2* mRNA expression in the HFD group compared to the CD group (*p =* 0.0340; Fig. S3j). Additionally, differences in *tph2* mRNA expression were found between the HFD and CD groups in the MnR [*F*_(31, 2535.8)_ = 4.30; *p* < 0.0001; Fig. S3f] and post hoc analysis indicated increased *tph2* mRNA at bregma levels − 7.916 (*p =* 0.036), − 7.832 (*p* < 0.001), and − 7.748 (*p =* 0.030). Further, the mean compiled *tph2* mRNA expression across the rostrocaudal extent of the MnR showed increased *tph2* mRNA expression in the HFD group compared to the CD (*p =* 0.0002; Fig. S3n). There were no differences in *tph2* mRNA expression between CD and HFD in the ventrolateral part of the DR (DRVL/VLPAG; Fig. S3c, k) or caudal part of the DR (DRC; Fig. S3d, l). Analysis of the mean compiled *tph2* mRNA expression in the interfascicular part of the DR (DRI; Fig. S3e, m) revealed increased expression in the HFD group (*p =* 0.0121). There were no differences in *tph2* mRNA expression between HFD and CD groups in the pontomesencephalic reticular formation (PMRF; Fig. S3g, o) or B9 serotonergic cell group (B9; Fig. S3h, p).

#### *htr1a* mRNA expression

Linear mixed model analysis of *htr1a* mRNA expression of compiled subregions of the DR, MnR, PMRF, and B9 cells showed effects of diet [*F*_(1, 106.5)_ = 47.5; *p* < 0.0001] and a diet x rostrocaudal level interaction [*F*_(15, 76.9)_ = 3.5; *p* < 0.0001] (Figure S4). Fisher’s LSD post hoc tests revealed differences in overall *htr1a* mRNA expression at bregma level − 7.748 (*p <* 0.0001), − 8.000 (*p <* 0.0001), and − 8.084 (*p <* 0.0001). Mean expression of *htr1a* mRNA compiled across all rostrocaudal levels of all subregions showed no difference (Fig. S4a, i).

Further analysis using a second LMM considering diet treatment on DR subdivisions revealed an increased *htr1a* mRNA expression in the cDRD [*F*_(1, 17.4)_ = 24.5; *p* < 0.0001; Fig. 4d] in line with the effects of diet treatment on *tph2* mRNA expression in this subregion of the DR that has been associated with increased anxiety-like states [13, 50]. Fisher’s LSD post hoc tests revealed differences between HFD and CD groups at bregma level − 8.000 (*p* < 0.003), − 8.084 (*p* < 0.0001) and − 8.168 (*p =* 0.015) of the cDRD. Analysis of the mean compiled *htr1a* mRNA expression across the rostrocaudal extent of the rDRD and cDRD subregions showed increased *htr1a* mRNA expression in the HFD group, relative to the CD group, in the DRD (*p <* 0.0001), rDRD (*p* = 0.0148) and cDRD (*p <* 0.0001; Fig. 4e).

LMM analysis also indicated increased *htr1a* mRNA expression in the cDRV [*F*_(6, 19.6)_ = 6.1; *p* = 0.001; Fig. S4b]. Fisher’s LSD showed increased *htr1a* mRNA expression in the HFD group compared to the CD group at bregma level − 7.916 (*p* = 0.010), − 8.000 (*p* < 0.0001), and − 8.084 (*p <* 0.0001; Fig. S4b). The mean compiled *htr1a* mRNA expression across the rostrocaudal extent of the rDRV and cDRV subregions showed increased *htr1a* mRNA expression in the HFD group, relative to the CD group, in the DRV (*p <* 0.0001), rDRV (*p* = 0.0145) and cDRV (*p <* 0.0001; Fig. S4b, j).

LMM analysis revealed an effect of diet on *htr1a* mRNA expression in the MnR [*F*_(15, 13.7)_ = 5.7; *p* = 0.001]. Fisher’s LSD post hoc comparisons showed increased *htr1a* mRNA expression in the HFD group compared to the CD group at bregma levels − 7.748 (*p* = 0.013), − 8.000 (*p* < 0.0001), and − 8.336 (*p* = 0.044; Fig. S4f). Analysis of the mean compiled *htr1a* mRNA expression across the rostrocaudal extent of the MnR indicated that the HFD group had increased *htr1a* mRNA expression compared to the CD group (*p* < 0.0001; Fig. S4n).

LMM analysis revealed an effect of diet on *htr1a* mRNA expression in the PMRF [*F*_(1, 13.5)_ = 5.3; *p* = 0.037]. Fisher’s LSD post hoc comparisons showed increased *htr1a* mRNA expression in the HFD group compared to the CD group at bregma levels − 7.748 (*p* = 0.005) and − 8.000 (*p =* 0.040; Fig. S4g). Mean compiled *htr1a* mRNA expression across PMRF extent showed increased expression of *htr1a* in the HFD compared to CD group (*p =* 0.0003; Fig. S4o).

LMM analysis revealed an effect of diet on *htr1a* mRNA expression in the B9 serotonergic cell group [*F*_(9, 8.64)_ = 5.2; *p* = 0.012]. Fisher’s LSD post hoc comparisons showed increased *htr1a* mRNA expression in the HFD group compared to the CD group at bregma levels − 7.748 (*p <* 0.001), and − 8.000 (*p =* 0.008; Fig. S4h). Analysis of the mean compiled *htr1a* mRNA expression across the rostrocaudal extent of the B9 serotonergic cell group revealed increased *htr1a* mRNA expression in the HFD group relative to the CD group (*p <* 0.0001; Fig. S4p).

No differences in *htr1a* mRNA expression were found between HFD and CD groups in the DRVL/VLPAG (Fig. S4c, k), in the DRC (Fig. S4d, l), or in the DRI (Fig. S4e, m).

#### *slc6a4* mRNA expression

Linear mixed model analysis of *slc6a4* mRNA expression of compiled DR, MnR, PMRF, and B9 cells showed global effects of diet [*F*_(1, 157.1)_ = 26.4; *p* < 0.0001], diet x rostrocaudal level [*F*_(15, 89.4)_ = 2.6; *p* = 0.0023], and diet x rostrocaudal level within sub-region [*F*_(53, 82.3)_ = 1.5; *p* = 0.0498] (Fig. S5). Fisher’s LSD post hoc tests pointed to differences in overall analysis at bregma levels − 8.420 (*p =* 0.026) and − 8.672 (*p =* 0.017; Fig. S5a). Mean expression of *slc6a4* mRNA compiled across all rostrocaudal levels of all subregions was increased in the HFD group compared to the CD group (*p <* 0.0001; Fig. S5i). Further analysis using a secondary LMM considering DR subdivisions pointed to an increased *slc6a4* mRNA expression in the rostral aspect of the DRD [rDRD; *F*_(5, 13.6)_ = 6.1; *p* = 0.004]. Fisher’s LSD post hoc comparisons showed differences between HFD and CD groups at bregma level − 7.664 (*p* = 0.047; Fig. 4g, i). The mean compiled *slc6a4* mRNA expression in the rDRD subregion was not different between HFD and CD groups (Fig. 4h). However, the compiled *slc6a4* mRNA expression in the DRD (*p =* 0.0093), and the cDRD was increased in the HFD group compared to the CD group (*p =* 0.0372; Fig. 4h).

LMM analysis also indicated increased *slc6a4* mRNA expression in the rostral aspect of the ventral part of the DR [(rDRV; *F*_(5, 11.9)_ = 3.8; *p* = 0.027]. Fisher’s LSD post hoc tests showed increased expression in the HFD group compared to the CD group at bregma level − 7.832 (*p* = 0.004; Fig. S5b). Further, the mean compiled *slc6a4* mRNA expression in the rDRV was increased in the HFD group compared to the CD group (*p =* 0.0317; Fig. S5j). Further, the mean compiled *slc6a4* mRNA expression in the DRV (*p =* 0.0026), and cDRV (*p =* 0.0162) was increased in the HFD group compared to the CD group (Fig. S5j). The LMM analysis in the MnR revealed effects of diet on *slc6a4* mRNA expression [*F*_(1, 17.1)_ = 5.8; *p* = 0.027] (Fig. S5f). Post hoc comparisons using Fisher’s LSD showed increased mRNA expression in the HFD group compared to the CD group at bregma levels − 7.748 (*p =* 0.035) and − 7.916 (*p =* 0.003; Fig. S5f). Compiled *slc6a4* mRNA expression across the rostrocaudal extent of the MnR indicated that the HFD group had increased *slc6a4* mRNA expression compared to the CD group (*p =* 0.0004; Fig. S5n).

Analysis of *slc6a4* mRNA expression in the DRI revealed effects of diet [*F*_(1, 14.1)_ = 9.4; *p* = 0.008] and a diet x rostrocaudal level interaction approached statistical significance [*F*_(4, 12.8)_ = 3.0; *p* = 0.055]. Post hoc analysis using Fisher’s LSD showed differences in *slc6a4* mRNA expression in HFD and CD groups at bregma levels − 8.504 (*p* = 0.004) and − 8.672 (*p =* 0.002) within the DRI (Fig. S5e). Mean compiled *slc6a4* mRNA expression in the DRI revealed increased expression in the HFD group compared to the CD group (*p =* 0.0005; Fig. S5m).

No differences were found between HFD and CD groups in the DRVL/VLPAG (Fig. S5c, k), in the B9 serotonergic cell group (B9; Fig. S5h, r), or in the PMRF (Fig. S5g, o). Although LMM analysis did not indicate an effect of diet on *slc6a4* mRNA in the DRC, analysis of the compiled mean *slc6a4* mRNA expression across rostrocaudal levels of the DRC revealed increased expression in the HFD group compared to the CD group (*p =* 0.0021; Fig. S5d, l).

Overall, our results show that a HFD increases *tph2, htr1a* and *slc6a4* mRNA gene expression in areas of the DR, including subregions (e.g. cDRD) involved in the regulation of anxiety-related behaviors. Further detailed atlases of *tph2, htr1a*, and *slc4a6* mRNA expression in rat brain can be seen elsewhere [15, 50, 51].

### High-fat diet feeding induced anxiety-related behavior in rats tested in the EPM but not the LD and OF tests

#### Elevated plus-maze

**Fig. 5:**
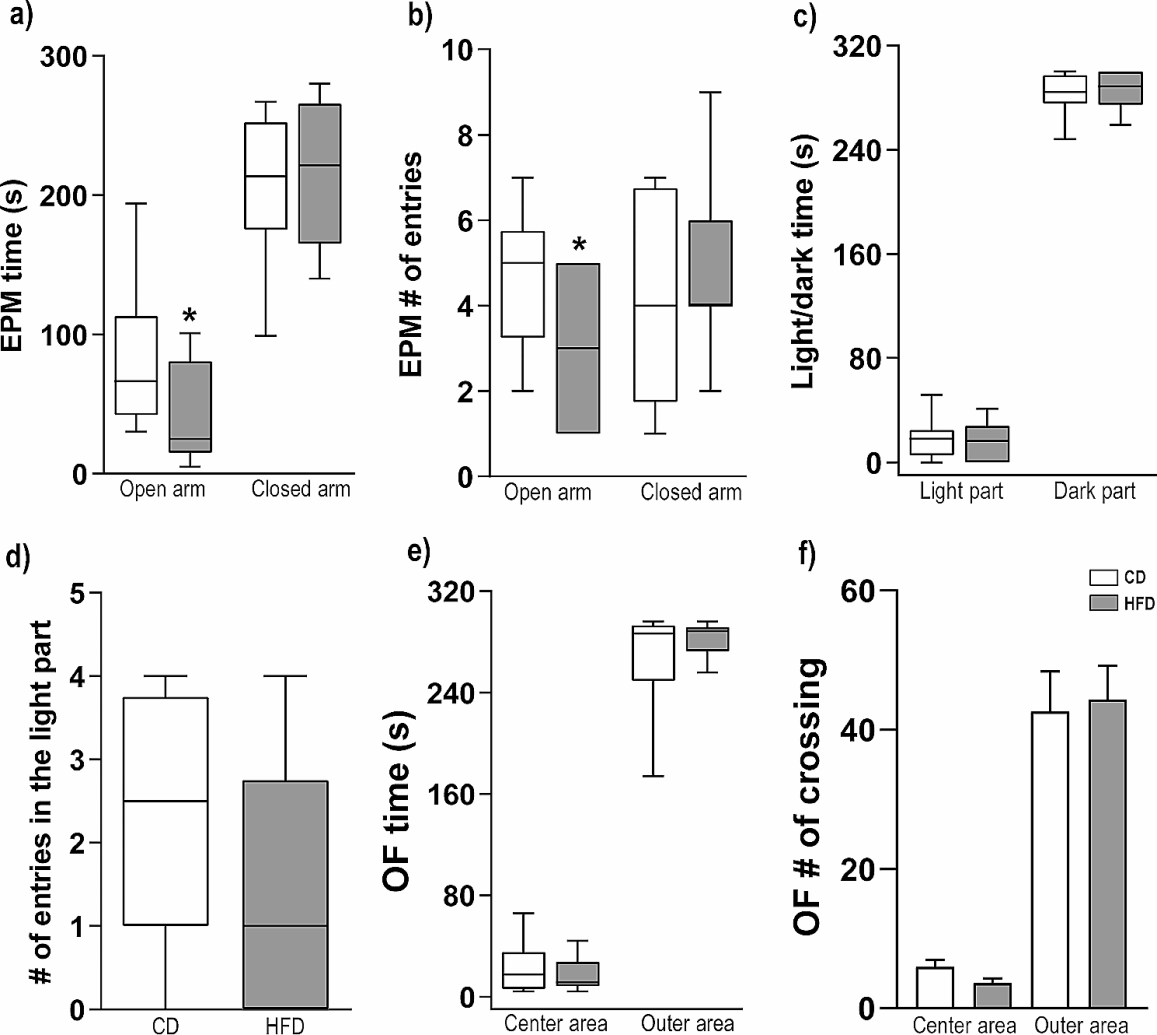
(**a-b**) Behavioral responses as measured by (**a**) time in the open and enclosed arms of the EPM, (**b**) number of entries in the open and enclosed arms of the EPM, (**c**) time in the light and dark part of the LD box, (**d**) number of entries in the light part of the LD box, (**e**) time in the center or outer area of the OF arena, (**f**) OF number of crossed squares. Data are expressed as means ± SEMs or interquartile ranges. g *p < 0.05. EPM, elevated plus-maze, LD, light-dark box; OF, open-field

Animals fed either with HFD or CD for 9 weeks were subjected to the EPM test (Fig. 5a, b). Our results corroborate the expected outcome that HFD increases anxiety-like defensive behavioral responses [4, 6], as measured by time spent on the open arms [*U*(24) = 33.00; *p* = 0.0232] and the number of entries in the open arms [*U*(24) = 38.50; *p* = 0.0440] of the EPM. Data with percent of time for both parameters are in Table S3. The time spent in the enclosed arm [*U*(24) = 67.00; *p* > 0.05] and the number of entries in the enclosed arm [*U*(24) = 61.50; *p* > 0.05] were not different between the HFD and CD animals (Fig. 5a, b).

#### Light/dark box test

To assess HFD effects on conflict anxiety-related behavior, animals were tested in the LDB as described previously [32]. The time spent in the light side of the LDB [*U*(24) = 70.00; *p* > 0.05], in the dark side of the LDB [*U*(24) = 60.50; *p* > 0.05] and the number of times animals crossed to light side [*U*(24) = 51.50; *p* > 0.05] were not different between HFD and CD groups, indicating that HDF did not affect the conflict anxiety-related behavior of rats exposed to the LDB (Fig. 5c-d). Rats fed a CD spent very little time in the light part of the LDB, suggesting a floor effect in this test.

#### Open-field test

Rats were also tested in the OF for 5 min. No differences were found between HFD and CD groups as measured by the time spent in the central area [*U*(24) = 62.00; *p* > 0.05; Fig. 5e] or the outer area [*U*(24) = 65.50; *p* > 0.05; Fig. 5e] during the 5 min test. Number of squares crossed by rats in the center [*t*(24) = 1.84; *p* > 0.05] or outer area [*t*(24) = 0.23; *p* > 0.05] of the OF showed no difference (Fig. 5f). Rats fed a CD spent very little time in the center of the OF, suggesting a floor effect in this test.

### Features of the gut microbial community shaped by HFD intake were associated with increased cDRD gene expression, an anxiety-related subregion of the DR

**Fig. 6:**
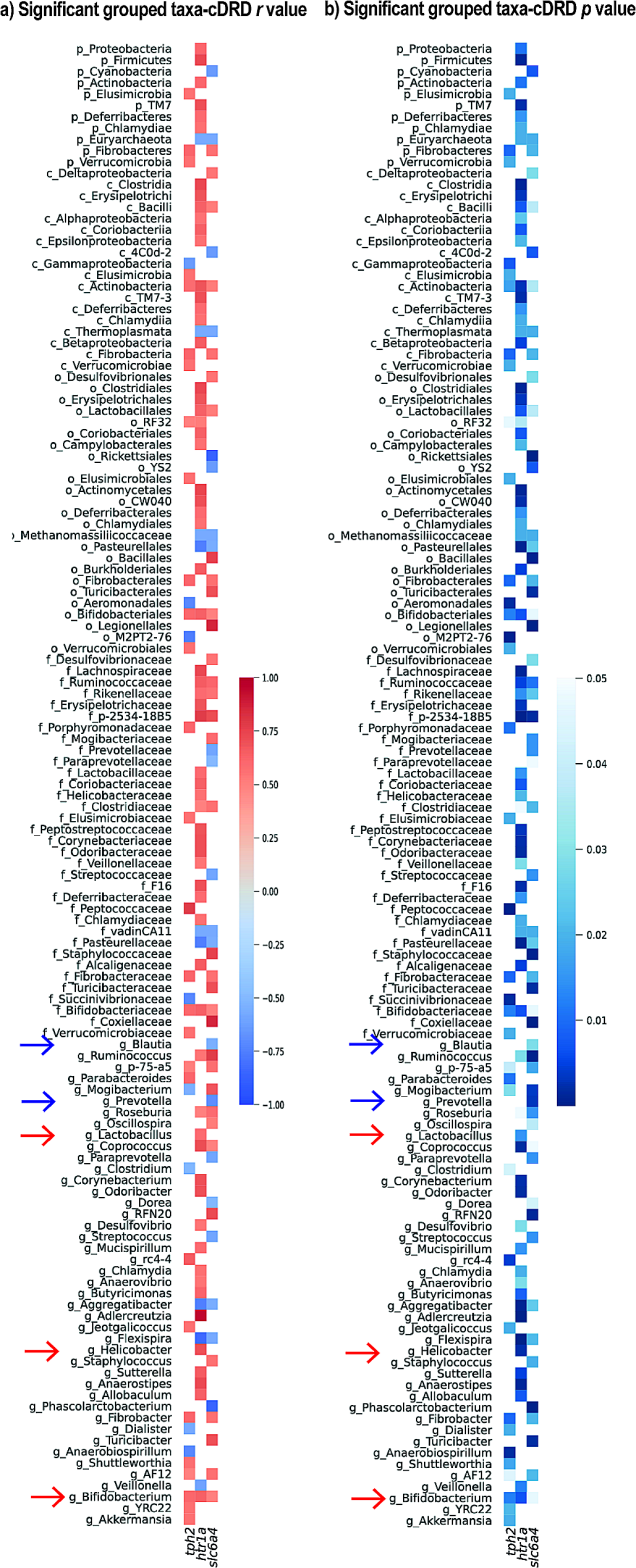
(**a, b**) Compiled significant Pearson’s correlation coefficient (blue and blue arrows, negative correlation; red and red arrows, positive correlation) among cDRD nuclei and relative abundance of different taxa for (**a**) grouped taxa *r* value, and (**b**) grouped taxa *p* value

Here we tested whether specific microbial taxa were correlated with gene expression in the anxiety-related cDRD subregion of the DR. Our results for Spearman’s correlation coefficient revealed that some genera were correlated with cDRD gene expression. We found that a range of taxa were correlated with the *tph2, htr1a*, and *slc6a4* gene expression in the cDRD (Fig. 6a-b). Importantly the changes in serotonergic gene expression were correlated with specific bacterial taxa (e.g., *Prevotella* genus; Fig. 6a-b), which were also correlated to the changes in behavior (Fig. S6). We also found a total of 31 genus level taxa to be positively correlated with mRNA expression in the cDRD while we found a total of 13 genus level taxa to be negatively correlated to mRNA expression in the cDRD. These results indicate significant associations between different taxonomic groups of gut bacteria and gene expression in the anxiety-related cDRD subregion.

We also evaluated potential associations between the Firmicutes/Bacteroidetes ratio among life stages and between adipose index and weight gain. Here we found a consistent F/B ratio throughout development in the CD group: CD-M (mean ± SD F/B ratio = 1.57 ± 0.16); CD-L (mean ± SD F/B ratio = 1.27 ± 0.10); and CD-A (mean ± SD F/B ratio = 1.67 ± 0.14). The F/B ratio was consistently higher in the HFD group: HFD-M (mean ± SD F/B ratio = 6.87 ± 0.20), HFD-L (mean ± SD F/B ratio = 10.84 ± 0.88), and HFD-A (mean ± SD F/B ratio = 6.71 ± 0.09). Mann-Whitney U-test comparison revealed that CD-L was lower in F/B ratio compared to HFD-L (*p* = 0.0063), and CD-A compared to HFD-A (*p* < 0.0001). Pearson’s correlation coefficient testing the association between F/B ratio in both groups was significant for adipose index (*p* < 0.0001) and weight gain (*p* = 0.0067; Fig. 3l-m). Together, these results suggest that HFD intake alters the gut microbiome community composition among life stages, which, in turn, is potentially associated with increased adiposity.

## Discussion

Here, we show that consumption of a HFD over a nine-week period, relative to a control diet condition, altered the gut microbial diversity and community composition. At the same time, we found that HFD intake affected serotonergic gene expression in the midbrain and pontine raphe nuclei, as evidenced by increased *tph2*, *htr1a*, and *slc6a4* mRNA expression in subregions of the DR associated with anxiety-like defensive behavioral responses, particularly the cDRD or dorsomedial DR, a subregion that gives rise to serotonergic projections to forebrain anxiety circuits. Finally, HFD induced anxiety-like behavioral responses in rats.

### High-fat diet impacted the gut microbiome

Here, we found HFD-induced obesity was associated with lower alpha diversity and altered community composition of the gut microbiome at later life stages, relative to CD controls. In addition, the gut microbiome’s composition varied from mid-adolescence to late adolescence to adulthood regardless of the diet regimen, with greater dissimilarity at the adult stage. The gut microbiome is a dynamic system that can be influenced by various factors such as host genetics, birth delivery method, medication, social interaction, environment, and diet [52]. Even though a greater dissimilarity in the microbiome was noted in HFD-rats relative to CD-rats, increased dissimilarity was also observed in CD-rats across life stages, highlighting the significance of the stage of life in microbiome community structure. After birth, in humans, the microbiome undergoes three stages – development, transition, and finally, stability [53]. Youth, adult, or senior populations have different gut microbiome compositions, which can be influenced by a range of age-associated factors [54]. Our data suggest that the HFD impacted the gut microbiome diversity and community composition across the 9 weeks of diet treatment. Several studies, in humans, have described environmental factors, such as diet, to have a major influence on the gut microbiome composition [55–57], and even more relevant, that the gut microbiome is one of the main players in health and disease outcomes, including anxiety in both experimental pre-clinical models and humans [58–60].

Here we found that the relative abundance of *Prevotella*, a genus previously associated with consumption of a diet high in fiber [61], is lower in HFD rats. Western-style diets (i.e., high in fat and/or sugar, low in fiber), can decrease beneficial bacteria, particularly species, such as *Prevotella* spp., that metabolize plant-derived polysaccharides to short-chain fatty acids (SCFAs) [62]. Dysregulation of the gut microbiome also impacts the host metabolism, by increasing energy production from macronutrients, and by being an active player in the low-grade inflammation response [63], a process that appears to be involved in the development of anxiety-related behaviors in animals fed a HFD [6].

In the present study we observed significantly lower relative abundance of members of the VANISH taxa, including the genus *Prevotella* (a member of the Bacteroidetes phylum) in HFD rats. Notably, the microbiome of human traditional hunter-gatherer populations contain taxa that have been lost or reduced in individuals living in industrialized societies [64]. These “lost taxa” have been termed “VANISH” (volatile and/or associated negatively with industrialized societies of humans) [64, 65]. *Prevotella* have been described as a potential biomarker of diet intake, positively associated with complex carbohydrates and fiber intake and capable of breaking down polysaccharides [61]. The presence of natural polysaccharides in the gut microenvironment has been shown to improve features of the gut microbiome by influencing the species predominance, strengthening intestinal barrier function, developing antioxidant activity, promoting SCFA production and reducing proinflammatory mediators [66].

The relative abundance of *Prevotella* was negatively correlated with *slc6a4* mRNA expression in the cDRD. Previous studies have shown that a double hit of adverse early life experience in rats, i.e., maternal separation, followed by an acute stress exposure (in this case, social defeat) induces an increase in *slc6a4* mRNA expression in the DR, including the cDRD region [15]. Thus, lower relative abundance of *Prevotella*, associated with higher expression of *slc6a4* mRNA expression, is consistent with a stress-like state with increased *slc6a4* mRNA expression.

We also found that *Lactobacillus* abundance, another VANISH taxa, was lower in HFD rats. *Lactobacillus* spp. can break down fructose and glucose, and its lower abundance has been linked to weight changes in obese individuals [67]. Interestingly, *Lactobacillus* spp. abundance varies among obese people of different origin, and an example is that higher levels are noted in those from Brazil compared to France [67]. Further, increased protein in the diet has been shown to be associated to higher *Lactobacillus* abundance, suggesting its relation to dietary factors [68]. Many species of the *Lactobacillus* genus provide probiotic benefits, including reducing inflammation and regulating immune system function, and also it was described that some strains within this genus may protect animals from novel stressors, promoting their health [68]. In our study, the genus *Blautia* had higher relative abundance in the HFD groups, suggesting its enrichment due to high-fat diet intake. In industrialized populations some taxa are enriched, which collectively have been termed BLoSSUM (Bloom or Selected in Societies of Urbanization/Modernization) taxa [65]. Higher relative abundances of *Blautia* may be related to increases in inflammation [69], and high symptom levels in depression [70].

Another BLoSSUM taxa [65], the Clostridiaceae family, was enriched in rats fed a HFD, specifically at the HFD-M and HFD-L stages. This result is in line with a recent study showing an increase in the relative abundance of the Clostridiaceae family in a high-anxiety mouse model [71]. Taken together, our findings closely resemble the adaptations in the microbiome caused by the transition from non-industrialized societies to a more industrialized society.

Our results showed an increase in the Firmicutes/Bacteroidetes (F/B) ratio in rats fed a HFD. The gut microbiome is dominated by two phyla – Firmicutes and Bacteroidetes [72]. The F/B ratio was previously reported to be associated with obesity, as Firmicutes are increased in HFD-fed individuals and enriched in genes coding for nutrient transporters, while Bacteroidetes are associated with a diet rich in fiber and consequently enriched in genes coding for carbohydrate metabolism [73, 74]. In this sense, the F/B ratio was described to be a useful tool for classifying obesity, although this has been contested [75]. Herein, we found positive correlations between F/B ratio and all fat pad tissues through the adipose index (i.e., sum of epididymal, inguinal and retroperitoneal fat pads) and final weight in HFD versus CD groups. Moreover, consistent with an increase in the F/B ratio in HFD animals, within the Firmicutes phylum, we found an increased relative abundance of *Blautia* in HFD animals, a genus that is reliably associated with abdominal fat accumulation [76]. While a high F/B ratio is not always found to be increased in obese individuals [77], humans studies have a series of confounding factors that can influence the microbiome composition, such as the use of antibiotics and other medications, and clinical conditions such as diabetes. Moreover, higher relative abundance of the Firmicutes phylum can also be observed in the United States of America population [78], which coincides with the increased incidence of obesity in this population [79].

### HFD increases brainstem serotonergic gene expression

Anxiety states and anxiety-like behavioral responses are associated with increases in serotonergic mRNA expression in the dorsomedial DR (also referred to as the cDRD [for discussion of nomenclature, see Table 4 [13]; see also [80]]. Increased activity of the cDRD induces anxiety-like defensive behavioral responses [13, 50]. Our results show animals fed a HFD had higher expression of *tph2, htr1a* and *slc6a4* in the anxiety-related cDRD subregion. This is consistent with the hypothesis that the anxiety-related responses observed in HFD-fed rats are, in part, caused by changes in serotonergic signaling within cDRD limbic circuits [80]. We have previously shown a chronic HFD intake from middle adolescence to adulthood induces neuroinflammation [6], which could increase serotonergic gene expression in the cDRD.

Higher expression of *tph2* in the cDRD is a particularly consistent finding in several chronic anxiety-like states in rodents, and in persons with affective disorders [21, 81]. For example, two different models of inescapable stress increase *tph2* mRNA expression in the cDRD in association with increased anxiety-like defensive behavioral responses [13]. In addition, corticotropin-releasing hormone (Crh) receptor priming in the bed nucleus of the stria terminalis (BNST) induces increases in *tph2* mRNA expression in the cDRD in association with a chronic anxiety-like state, and *tph2* mRNA expression in the cDRD is highly correlated with anxiety-like defensive behavioral responses [14]. Furthermore, maternal separation during early life, which induces a chronic anxiety-like state throughout adulthood, increases *tph2* mRNA expression in the cDRD in rats [15]. Moreover, chronic administration of corticosterone, which induces a chronic anxiety-like state, induces an increase in *tph2* mRNA expression in the DR, although these effects are not restricted to the cDRD region [82]. Finally, microinjection of the anxiety- and fear-related peptide, Crh, directly into the cDRD region increases serotonin release in the central nucleus of the amygdala, which is temporally associated with fear-related behavioral responses [19]. Studies in humans have found increased expression of CRH in the caudal DR of persons with depression who died by suicide, suggesting that the caudal DR also may play a role in stress-related psychiatric disorders and suicide risk in humans [83]. Other studies have found increased *tph2* mRNA [84] and protein expression [81] in the caudal part of the DR in persons with depression who died by suicide, while in alcohol-dependent persons with depression who died by suicide, increases in TPH2 protein are restricted to the cDRD region [21].

HFD animals also had higher expression of the *htr1a* gene in the cDRD nucleus. Although the connection between *htr1a* expression and chronic anxiety is not well understood, studies have shown that single nucleotide polymorphisms in the *HTR1A* gene, along with recent stressful life events, were associated with higher depression and anxiety scores [85, 86]. This suggests that the 5-HT_1A_ receptor confers vulnerability to anxiety and mood disorders by modulating threat-related information processing [86].

Rats fed a HFD also presented with higher *slc6a4* expression in the cDRD. Previous studies in rats have shown that exposure to adverse early life experiences, caused by maternal separation, followed by acute social defeat stress in adulthood, results in increased *slc6a4* mRNA expression in the cDRD relative to animal facility reared controls and to rats exposed to neonatal handling [15]. Increased expression of *tph2*, *htr1a*, and *slc6a4* in the cDRD of HFD-fed rats is consistent with previous studies implicating the cDRD in chronic (even lifelong) anxiety-like states, and vulnerability to future stress exposures in adulthood. Early life adverse experiences increase the risk of developing stress-related psychiatric disorders, including anxiety disorders [87]. In this sense, consumption of a HFD during adolescence could be an important factor increasing susceptibility to developing stress-related psychiatric disorders later in life.

Changes in the gut microbiome taxa were correlated with changes in expression of serotonergic genes, including *tph2*, *htr1a*, and *slc6a4* in the cDRD subregion. Specific taxa were differently associated with up- or down-regulated gene expression. Of note, the family, Verrucomicrobiaceae, and *Akkermansia*, a genus within the Verrucomicrobiaceae family, was positively correlated with *tph2* gene expression. On the other hand, relative abundances of the genus *Prevotella* was negatively correlated with *slc6a4* gene expression in the cDRD. Our results provide new insights into a role of the microbiome-gut-brain axis in regulating anxiety-related behaviors by pointing to a possible participation of the brain serotonergic systems in this process.

### HFD induces anxiety-related behavioral responses

Rats exposed to consumption of a HFD responded with increased anxiety-related behaviors in the EPM; however, this effect was not observed in the LDB and OF. As previously proposed, each paradigm investigates different aspects of aversive responses, and the stress associated with the repeated handling and testing in the behavioral test battery could have impaired our ability to detect HFD-induced changes in approach and avoidance behaviors, due to repeated exposure to threatening stimuli [88]. Indeed, rats exposed to the CD condition had very high baseline measures of anxiety-like defensive behavioral responses in both the LDB and OF, which may have resulted in a floor effect. Our results using the EPM, however, corroborate others that investigated weight gain (i.e., increased adiposity) followed by increases in anxiety-like defensive behavioral responses [89, 90]. Importantly, the developmental period during which dietary intervention is introduced might impact both gut microbiome and brain development. In the present study we initiated HFD around five weeks of age, equivalent to the mid-adolescent period, corroborating studies that have consistently shown that introducing a HFD early during development impacts a range of responses, such as spatial learning, memory and anxiety-like defensive behavioral responses [4, 6, 91, 92].

### Limitations

Our work has a number of important limitations. First, the fecal pellet collection started on the second day of the diet protocol, meaning that rats from the HFD group were eating a HFD for at least 48 h before the first fecal pellet collection, which is sufficient time for diet to impact the community composition of the gut microbiome [93]. However, in our understanding, this limitation does not decrease the relevance of our findings, as the diet treatment lasted for 9 weeks, and the serotonergic gene expression and behavior assessments were performed only at the end of this period. A second limitation was that we did not directly investigate mechanisms, such as afferent neuronal signaling, microbiome-derived metabolites, or altered immune signaling derived from the microbiome that could be involved in the changes in serotonergic gene expression and anxiety-like behavioral responses. Finally, the study was conducted using male rats across mid-adolescence to the adult period; it is therefore not clear to what extent these data would generalize to female rats or other life stages. Our initial question aimed to limit the variety of uncontrolled parameters, including estrous cycling in female rats [see, for example [94]], and in this sense we moved to investigate male rats in our initial studies. Given that the incidence of anxiety disorders, mood disorders, and trauma-and stressor-related disorders are higher in females than in males [95], this is an important limitation and sex as a biological variable represents an important direction for future research.

## Conclusion and clinical implications

The present study showed that a 9-week exposure to a HFD altered gut microbiome diversity and community composition, with changes in community composition that closely align with those seen in industrialized human microbiomes, including decreases in VANISH taxa, increases in BLoSSUM taxa, and increases in the F/B ratio. Consumption of a HFD was also associated with changes in serotonergic gene expression in the brainstem raphe nuclei, including the anxiety-related cDRD or dorsomedial DR, and anxiety-like behavioral responses. Considering the early introduction of high-fat foods in children’s diets, and the ever-increasing obesity epidemic, our data introduce a possible scenario by which the dietary choices during adolescence can influence the gut microbiome, brainstem serotonergic systems, and the susceptibility to the development of psychiatric disorders in adulthood. This knowledge could lead to new microbiome-based approaches to prevent stress-related psychiatric disorders such as anxiety disorders.

### Electronic supplementary material

Below is the link to the electronic supplementary material.

**Supplementary Material 1**: **Supplementary Fig. 1.** Atlases of serotonergic gene expression. (a) Atlas of rat tryptophan hydroxylase 2 (*tph2*) mRNA expression, (b) atlas of rat 5-HT_1A_ receptor (*htr1a*) mRNA expression, and (c) atlas of rat serotonin transporter (*slc6a4*) mRNA expression in the midbrain and pontine raphe complex (84 μm intervals) used for analysis of subregions of the dorsal raphe nucleus (DR), median raphe nucleus (MnR), pontomesencephalic reticular formation (PMRF) and B9 serotonergic cell group (B9) with a high level of neuroanatomical resolution. Photographs are autoradiographic images of *tph2*, *htr1a*, and *slc6a4* mRNA expression. The levels chosen for analysis ranged from (A) − 7.412 mm bregma through (P) − 8.672 mm bregma. Dotted lines delineate different subdivisions of the DR analyzed in this study, based on a stereotaxic atlas of the rat brain [39]. Abbreviations: B9, supralemniscal serotonergic cell group; MnR, median raphe nucleus; DRC, dorsal raphe nucleus, caudal part; DRD, dorsal raphe nucleus, dorsal part; DRI, dorsal raphe nucleus, interfascicular part; DRV, dorsal raphe nucleus, ventral part; DRVL, dorsal raphe nucleus, ventrolateral part; PMRF, pontomesencephalic reticular formation; VLPAG, ventrolateral periaqueductal gray. Numbers in the upper right of each panel indicate the rostrocaudal coordinates relative to bregma (in mm). Numbers in the lower right of each panel indicate the rostrocaudal level of each DR. Scale bar, 1 mm. The rostrocaudal levels and matrices defining subregions of the DR that were defined using *tph2* autoradiograms were also used in the analysis of *htr1a* and *slc6a4* to ensure sampling of the same anatomical regions for each gene

**Supplementary Material 2**: **Supplementary Fig. 2**. Effects of high-fat diet (HFD) on alpha diversity, beta diversity, and community composition of the gut microbiome across mid-adolescence, late adolescence, and adulthood. (a) Beta diversity distance comparison plot with box plots illustrating distances within samples and between groups using Weighted UniFrac distance, (b) Weighted UniFrac PCoA plot of dissimilarity matrix, and (c) age-arranged weighted UniFrac PCoA plot of dissimilarity matrix. (d) Beta diversity distance comparison plot with box plots illustrating distances within samples and between groups using Unweighted UniFrac distance, (e) Unweighted UniFrac PCoA plot of dissimilarity matrix, and (f) age-arranged Unweighted UniFrac PCoA plot of dissimilarity matrix. Top ten taxa with highest relative abundances illustrated by stacked vertical bar charts for (g) class, (h) order, (i) family, and (j) genus. Data are expressed as boxplots, where bottom and tops of boxes indicate the first and third quartiles, respectively; whiskers indicate the interquartile range (IQR) beyond the upper and lower quartiles. PERMANOVA pairwise test. Abbreviations: CD-A, control diet group/adulthood; CD-L, control diet group/late adolescence; CD-M, control diet group/middle adolescence; HFD-A, high-fat diet group/adulthood; HFD-L, high-fat diet group/late adolescence; HFD-M, high-fat diet group/middle adolescence

**Supplementary Material 3**: **Supplementary Fig. 3.** Effects of nine weeks of a control diet (CD) or high-fat diet (HFD) protocol on *tph2* mRNA expression in subdivisions of the dorsal raphe nucleus (DR), median raphe nucleus (MnR), pontomesencephalic reticular formation (PMRF), and B9 serotonergic cell group. Each graph represents the means ± SEMs of *tph2* mRNA expression levels at specific rostrocaudal levels or within subregions. Graphs illustrate *tph2* mRNA expression in the (a) total DR, MnR, PMRF, and B9 supralemniscal serotonergic cell group, (b) dorsal raphe nucleus, ventral part (DRV), including the rostral (rDRV) and caudal (cDRV) aspects, (c) dorsal raphe nucleus, ventrolateral part (DRVL)/ventrolateral periaqueductal gray (VLPAG), (d) dorsal raphe nucleus, caudal part (DRC), (e) dorsal raphe nucleus, interfascicular part (DRI), (f) MnR, (g) PMRF, and (h) B9 supralemniscal serotonergic cell group. Further compiled levels are shown for (i) total rostrocaudal levels of DR, MnR, PMRF, and B9 serotonergic cell group bar plot, (j) DRV, rDRV and cDRV bar plots, (k) DRVL/VLPAG bar plots, (l) DRC bar plot, (m) DRI bar plot, (n) MnR bar plot, (o) PMRF bar plot, and (p) B9 supralemniscal serotonergic cell group bar plot. *p < 0.05 versus CD at the same rostrocaudal level (a-h); versus CD based on compiled *tph2* mRNA expression across rostrocaudal levels (i-p), white circles/bars represent CD group, and black circles/bars represent HFD group. Rostrocaudal levels 9 = − 7.412 mm, 8 = − 7.496 mm, 7 = − 7.580 mm, 6 = − 7.664 mm, 5 = − 7.748 mm, 4 = − 7.832 mm, 3 = − 7.916 mm, 2 = − 8.00 mm, 1 = − 8.084 mm, 0 = − 8.168 mm, − 1 = − 8.252 mm, − 2 = − 8.336 mm, − 3 = − 8.420 mm, − 4 = − 8.504 mm, − 5 = − 8.588 mm, and − 6 = − 8.672 mm. Sample sizes for each treatment group at each rostrocaudal level of analysis are shown on the upper section of the panel (a-h). Abbreviations: CD, control diet; HFD, high-fat diet

**Supplementary Material 4**: **Supplementary Fig. 4**. Effects of nine weeks of a control diet (CD) or high-fat diet (HFD) protocol on *htr1a* mRNA expression in subdivisions of the dorsal raphe nucleus (DR), median raphe nucleus (MnR), pontomesencephalic reticular formation (PMRF), and B9 serotonergic cell group. Each graph represents the means ± SEMs of *htr1a* mRNA expression levels at specific rostrocaudal levels or within subregions. Graphs illustrate *htr1a* mRNA expression in the (a) total combined expression in dorsal raphe nucleus (DR), median raphe nucleus (MnR), pontomesencephalic reticular formation (PMRF), and B9 supralemniscal serotonergic cell group, (b) dorsal raphe nucleus, ventral part (DRV), including the rostral (rDRV) and caudal (cDRV) aspects, (c) dorsal raphe nucleus, ventrolateral part (DRVL)/ventrolateral periaqueductal gray (VLPAG), (d) dorsal raphe nucleus, caudal part (DRC), (e) dorsal raphe nucleus, interfascicular part (DRI), (f) MnR, (g) PMRF, and (h) B9 supralemniscal serotonergic cell group. Further compiled levels are shown for (i) total rostrocaudal levels of DR bar plot, (j) DRV, rDRV and cDRV bar plots, (k) DRVL/VLPAG bar plots, (l) DRC bar plot, (m) DRI bar plot, (n) MnR bar plot, (o) PMRF bar plot, and (p) B9 supralemniscal serotonergic cell group bar plot. *p < 0.05, versus CD at the same rostrocaudal level (a-h); versus CD based on compiled *htr1a* mRNA expression across rostrocaudal levels (i-p), white circles/bars represent CD group, and black circles/bars represent HFD group. Rostrocaudal levels 9 = − 7.412 mm, 8 = − 7.496 mm, 7 = − 7.580 mm, 6 = − 7.664 mm, 5 = − 7.748 mm, 4 = − 7.832 mm, 3 = − 7.916 mm, 2 = − 8.00 mm, 1 = − 8.084 mm, 0 = − 8.168 mm, − 1 = − 8.252 mm, − 2 = − 8.336 mm, − 3 = − 8.420 mm, − 4 = − 8.504 mm, − 5 = − 8.588 mm, and − 6 = − 8.672 mm. Sample sizes for each treatment group at each rostrocaudal level of analysis are shown on the upper section of the panel (a-h). Abbreviations: CD, control diet; HFD, high-fat diet

**Supplementary Material 5**: **Supplementary Fig. 5.** Effects of nine weeks of a control diet (CD) or high-fat diet (HFD) protocol on *slc6a4* mRNA expression in subdivisions of the dorsal raphe nucleus (DR), median raphe nucleus (MnR), pontomesencephalic reticular formation (PMRF), and B9 supralemniscal serotonergic cell group. Each graph represents the means ± SEMs of *slc6a4* mRNA expression levels at specific rostrocaudal levels or within subregions. Graphs illustrate *slc6a4* mRNA expression in the (a) total combined expression in the DR, MnR, PMRF, and B9 supraleminiscal serotonergic cell group, (b) dorsal raphe nucleus, ventral part (DRV), including the rostral (rDRV) and caudal (cDRV) aspects, (c) dorsal raphe nucleus, ventrolateral part (DRVL)/ventrolateral periaqueductal gray (VLPAG), (d) dorsal raphe nucleus, caudal part (DRC), (e) dorsal raphe nucleus, interfascicular part (DRI), (f) MnR, (g) PMRF, and (h) B9 supralemniscal serotonergic cell group. Further compiled levels are shown for (i) total rostrocaudal levels of DR bar plot, (j) DRV, rDRV and cDRV bar plots, (k) DRVL/VLPAG bar plots, (l) DRC bar plot, (m) DRI bar plot, (n) MnR bar plot, (o) PMRF bar plot, and (p) B9 supralemniscal serotonergic cell group bar plot. *p < 0.05 versus CD at the same rostrocaudal level (a-h); versus CD based on compiled *slc6a4* mRNA expression across rostrocaudal levels (i-p), white circles/bars represent CD group, and black circles/bars represent HFD group. Rostrocaudal levels 9 = − 7.412 mm, 8 = − 7.496 mm, 7 = − 7.580 mm, 6 = − 7.664 mm, 5 = − 7.748 mm, 4 = − 7.832 mm, 3 = − 7.916 mm, 2 = − 8.00 mm, 1 = − 8.084 mm, 0 = − 8.168 mm, − 1 = − 8.252 mm, − 2 = − 8.336 mm, − 3 = − 8.420 mm, − 4 = − 8.504 mm, − 5 = − 8.588 mm, and − 6 = − 8.672 mm. Sample sizes for each treatment group at each rostrocaudal level of analysis are shown on the upper section of the panel (a-h). Abbreviations: CD, control diet; HFD, high-fat diet

**Supplementary Material 6**: **Supplementary Fig. 6**. Analysis for rats treated with control diet (CD) and high-fat diet (HFD) to reveal correlations of the time spent in the open-arm of the elevated plus-maze versus relative abundance of specific taxa. Each graph represents Spearman’s correlation coefficient of time in the open arm versus relative abundance of specific taxa, and *r* and *p* values are shown on the panels according to the analysis of time spent in the open-arm of the elevated plus-maze x taxa, separately for CD and HFD. (a-b) CD taxa x open arm correlation, (c-d) HFD taxa x open arm correlation. (e-f) CD and HFD taxa combined x open arm correlation. (g) Analysis of correlation between time in the open arm versus Prevotella relative abundance. (h) *slc6a4* gene expression versus Prevotella. For *r* values, blue represents negative correlations and red positive correlations; for *p* values heatmap, blue scale represents variations within 0.05 and 0.01. Abbreviations: CD, control diet; HFD, high-fat diet

**Supplementary Material 7**:

**Supplementary Material 8**

## Data Availability

Code used in this study can be found in the GitHub address https://github.com/bioinfonupeb/hfd-insitu-microbiome. All other data and code may be provided by the corresponding author on reasonable request.

## Abbreviations

5-HT: 5-hydroxytryptamine, 5-HT, serotonin
ACTH: adrenocorticotropic hormone
AI: adiposity index
B9: B9 serotonergic cell group, supralemniscal serotonergic cell group
BDNF: Brain-derived neurotrophic factor
BloSSUM: Bloom or Selected in Societies of Urbanization/Modernization
CD: Control diet
CD-A: Control diet group/adulthood
CD-L: Control diet group/late adolescence
CD-M: Control diet group/middle adolescence
cDRD: Caudal aspect of the dorsal raphe nucleus, dorsal part; dorsomedial part of the dorsal raphe nucleus
cDRV: Caudal aspect of the dorsal raphe nucleus, ventral part
CORT: Corticosterone
DR: Dorsal raphe nucleus
DRC: Dorsal raphe nucleus, caudal part
DRD: Dorsal raphe nucleus, dorsal part
DRI: Dorsal raphe nucleus, interfascicular part
DRV: Dorsal raphe nucleus, ventral part
DRVL/VLPAG: Dorsal raphe nucleus, ventrolateral part/ventrolateral periaqueductal gray region
EPM: Elevated plus-maze
F/B: Firmicutes/Bacteroidetes
HFD: High-fat diet
HFD-A: High-fat diet group/adulthood
HFD-L: High-fat diet group/late adolescence
HFD-M: High-fat diet group/middle adolescence
*htr1a*: Gene encoding serotonin (5-hydroxytryptamine; 5-HT) receptor 1 A
LDB: Light/dark box
MGB: Microbiome-gut-brain
MnR: Median raphe nucleus
OF: Open-field
PMRF: Pontomesencephalic reticular formation
rDRD: Rostral aspect of the dorsal raphe nucleus, dorsal part
rDRV: Rostral aspect of the dorsal raphe nucleus, ventral part
*slc6a4*: Gene encoding solute carrier family 6 member 4, serotonin transporter
*tph2*: Gene encoding tryptophan hydroxylase 2
VANISH: Volatile and/or Associated Negatively with Industrialized Societies of Humans

## References

[1] Castanon N, Lasselin J, Capuron L. Neuropsychiatric comorbidity in obesity: role of inflammatory processes. Front Endocrinol (Lausanne) 2014;5:74. 10.3389/fendo.2014.00074 PMC4030152 24860551

[2] Amiri S, Behnezhad S (2019). Obesity and anxiety symptoms: a systematic review and meta-analysis. Neuropsychiatrie.

[3] Santomauro DF, Herrera AMM, Shadid J, Zheng P, Ashbaugh C, Pigott DM, Abbafati C, Adolph C, Amlag JO, Aravkin AY (2021). Global prevalence and burden of depressive and anxiety disorders in 204 countries and territories in 2020 due to the COVID-19 pandemic. Lancet.

[4] de Noronha SR, Campos GV, Abreu AR, de Souza AA, Chianca DA, de Menezes RC (2017). High fat diet induced-obesity facilitates anxiety-like behaviors due to GABAergic impairment within the dorsomedial hypothalamus in rats. Behav Brain Res.

[5] Haleem DJ, Mahmood K (2021). Brain serotonin in high-fat diet-induced weight gain, anxiety and spatial memory in rats. Nutr Neurosci.

[6] Noronha SSR, Lima PM, Campos GSV, Chirico MTT, Abreu AR, Figueiredo AB, Silva FCS, Chianca DA Jr., Lowry CA, De Menezes RCA. Association of high-fat diet with neuroinflammation, anxiety-like defensive behavioral responses, and altered thermoregulatory responses in male rats. Brain, behavior, and immunity 2019;80:500–511. 10.1016/j.bbi.2019.04.030 31022457

[7] Foster JA, McVey Neufeld K-A (2013). Gut–brain axis: how the microbiome influences anxiety and depression. Trends Neurosci.

[8] Peirce JM, Alviña K (2019). The role of inflammation and the gut microbiome in depression and anxiety. J Neurosci Res.

[9] Ahmadi S, Wang S, Nagpal R, Wang B, Jain S, Razazan A, Mishra SP, Zhu X, Wang Z, Kavanagh K (2020). A human-origin probiotic cocktail ameliorates aging-related leaky gut and inflammation via modulating the microbiota/taurine/tight junction axis. JCI Insight.

[10] Yang Y, Du L, Shi D, Kong C, Liu J, Liu G, Li X, Ma Y (2021). Dysbiosis of human gut microbiome in young-onset colorectal cancer. Nat Commun.

[11] Alam R, Abdolmaleky HM, Zhou JR (2017). Microbiome, inflammation, epigenetic alterations, and mental diseases. Am J Med Genet Part B: Neuropsychiatric Genet.

[12] O’Mahony SM, Clarke G, Borre YE, Dinan TG, Cryan JF (2015). Serotonin, tryptophan metabolism and the brain-gut-microbiome axis. Behav Brain Res.

[13] Donner NC, Kubala KH, Hassell JE, Lieb MW, Nguyen KT, Heinze JD, Drugan RC, Maier SF, Lowry CA (2018). Two models of inescapable stress increase tph2 mRNA expression in the anxiety-related dorsomedial part of the dorsal raphe nucleus. Neurobiol Stress.

[14] Donner NC, Davies SM, Fitz SD, Kienzle DM, Shekhar A, Lowry CA (2020). Crh receptor priming in the bed nucleus of the stria terminalis (BNST) induces tph2 gene expression in the dorsomedial dorsal raphe nucleus and chronic anxiety. Prog Neuropsychopharmacol Biol Psychiatry.

[15] Gardner KL, Hale MW, Lightman SL, Plotsky PM, Lowry CA (2009). Adverse early life experience and social stress during adulthood interact to increase serotonin transporter mRNA expression. Brain Res.

[16] Paxinos G, Xu-Feng H, Sengul G, Watson C, Edition) JK, Mai, Paxinos G (2012). Chapter 8 - Organization of Brainstem nuclei. The human nervous system.

[17] Bacqué-Cazenave J, Bharatiya R, Barrière G, Delbecque J-P, Bouguiyoud N, Di Giovanni G, Cattaert D, De Deurwaerdère P (2020). Serotonin in animal cognition and behavior. Int J Mol Sci.

[18] Hale MW, Shekhar A, Lowry CA (2012). Stress-related serotonergic systems: implications for symptomatology of anxiety and affective disorders. Cell Mol Neurobiol.

[19] Forster GL, Pringle RB, Mouw NJ, Vuong SM, Watt MJ, Burke AR, Lowry CA, Summers CH, Renner KJ (2008). Corticotropin-releasing factor in the dorsal raphe nucleus increases medial prefrontal cortical serotonin via type 2 receptors and median raphe nucleus activity. Eur J Neurosci.

[20] Fox JH, Lowry CA (2013). Corticotropin-releasing factor-related peptides, serotonergic systems, and emotional behavior. Front Neurosci.

[21] Bonkale WL, Turecki G, Austin MC (2006). Increased tryptophan hydroxylase immunoreactivity in the dorsal raphe nucleus of alcohol-dependent, depressed suicide subjects is restricted to the dorsal subnucleus.

[22] Gutknecht L, Kriegebaum C, Waider J, Schmitt A, Lesch KP (2009). Spatio-temporal expression of tryptophan hydroxylase isoforms in murine and human brain: convergent data from Tph2 knockout mice. Eur Neuropsychopharmacol.

[23] Mosienko V, Bader M, Alenina N. Chapter 35 - The serotonin-free brain: behavioral consequences of Tph2 deficiency in animal models. In Handbook of Behavioral Neuroscience, C.P. Müller, and K.A. Cunningham, eds. (Elsevier), 2020, pp. 601–607.

[24] Albert PR, Le François B, Millar AM (2011). Transcriptional dysregulation of 5-HT1A autoreceptors in mental illness. Mol Brain.

[25] Donaldson ZR, Piel DA, Santos TL, Richardson-Jones J, Leonardo ED, Beck SG, Champagne FA, Hen R. Developmental Effects of Serotonin 1A Autoreceptors on Anxiety and Social Behavior. Neuropsychopharmacology 2014;39:291–302. 10.1038/npp.2013.185 PMC3870787 23907404

[26] Lesch KP (2011). When the serotonin transporter gene meets adversity: the contribution of animal models to understanding epigenetic mechanisms in affective disorders and resilience. Curr Top Behav Neurosci.

[27] Angles MR, Ocana DB, Medellin BC, Tovilla-Zarate C (2012). No association between the HTR1A gene and suicidal behavior: a meta-analysis. Braz J Psychiatry.

[28] Mohammad F, Ho J, Woo JH, Lim CL, Poon DJJ, Lamba B, Claridge-Chang A (2016). Concordance and incongruence in preclinical anxiety models: systematic review and meta-analyses. Neurosci Biobehav Rev.

[29] Licht CL, Mortensen EL, Hjordt LV, Stenbæk DS, Arentzen TE, Nørremølle A, Knudsen GM. Serotonin transporter gene (SLC6A4) variation and sensory processing sensitivity—comparison with other anxiety-related temperamental dimensions. Mol Genet Genom Med 2020;8:e1352. 10.1002/mgg3.1352 PMC7434600 32543106

[30] Homberg JR, Lesch K-P (2011). Looking on the bright side of serotonin transporter gene variation. Biol Psychiatry.

[31] Spear LP (2000). The adolescent brain and age-related behavioral manifestations. Neurosci Biobehav Rev.

[32] Reber SO, Siebler PH, Donner NC, Morton JT, Smith DG, Kopelman JM, Lowe KR, Wheeler KJ, Fox JH, Hassell JE (2016). Immunization with a heat-killed preparation of the environmental bacterium Mycobacterium vaccae promotes stress resilience in mice. Proc Natl Acad Sci U S A.

[33] Bouwknecht JA, Spiga F, Staub DR, Hale MW, Shekhar A, Lowry CA (2007). Differential effects of exposure to low-light or high-light open-field on anxiety-related behaviors: relationship to c-Fos expression in serotonergic and non-serotonergic neurons in the dorsal raphe nucleus. Brain Res Bull.

[34] Gould TD, Dao DT, Kovacsics CE, Gould TD (2009). The Open Field Test. Mood and anxiety related phenotypes in mice: characterization using behavioral tests.

[35] Caporaso JG, Kuczynski J, Stombaugh J, Bittinger K, Bushman FD, Costello EK, Fierer N, Pena AG, Goodrich JK, Gordon JI (2010). QIIME allows analysis of high-throughput community sequencing data. Nat Methods.

[36] Day HE, Akil H (1996). Differential pattern of c-fos mRNA in rat brain following central and systemic administration of interleukin-1-beta: implications for mechanism of action. Neuroendocrinology.

[37] Arnold MR, Williams PH, McArthur JA, Archuleta AR, O’Neill CE, Hassell JE, Smith DG, Bachtell RK, Lowry CA (2019). Effects of chronic caffeine exposure during adolescence and subsequent acute caffeine challenge during adulthood on rat brain serotonergic systems. Neuropharmacology.

[38] Blakely RD, Berson HE, Fremeau RT, Caron MG, Peek MM, Prince HK, Bradley CC (1991). Cloning and expression of a functional serotonin transporter from rat brain. Nature.

[39] Paxinos GW (1998). The rat brain in stereotaxic coordinates.

[40] Abrams JK, Johnson PL, Hollis JH, Lowry CA (2004). Anatomic and functional topography of the dorsal raphe nucleus. Ann N Y Acad Sci.

[41] Grubbs FE (1969). Procedures for detecting outlying observations in samples. Technometrics.

[42] Edgar RC (2013). UPARSE: highly accurate OTU sequences from microbial amplicon reads. Nat Methods.

[43] Wang Q, Garrity GM, Tiedje JM, Cole JR (2007). Naive bayesian classifier for rapid assignment of rRNA sequences into the new bacterial taxonomy. Appl Environ Microbiol.

[44] McDonald D, Price MN, Goodrich J, Nawrocki EP, DeSantis TZ, Probst A, Andersen GL, Knight R, Hugenholtz P (2012). An improved Greengenes taxonomy with explicit ranks for ecological and evolutionary analyses of bacteria and archaea. ISME J.

[45] Lozupone C, Knight R (2005). UniFrac: a new phylogenetic method for comparing microbial communities. Appl Environ Microbiol.

[46] Faith DP (1992). Conservation evaluation and phylogenetic diversity. Biol Conserv.

[47] Liu S, Qin P, Wang J. High-Fat Diet alters the intestinal microbiota in Streptozotocin-Induced type 2 Diabetic mice. Microorganisms 2019, 7. 10.3390/microorganisms7060176 PMC6617241 31208113

[48] Zhang C, Zhang M, Pang X, Zhao Y, Wang L, Zhao L (2012). Structural resilience of the gut microbiota in adult mice under high-fat dietary perturbations. ISME J.

[49] Zheng Z, Lyu W, Ren Y, Li X, Zhao S, Yang H, Xiao Y. Allobaculum involves in the modulation of intestinal ANGPTLT4 expression in mice treated by High-Fat Diet. 2021, 8. 10.3389/fnut.2021.690138 PMC8171929 34095196

[50] Hassell JE, Yamashita PSM, Johnson PL, Zangrossi H, Shekhar A, Lowry CA, Fink G (2017). Chapter 15 - stress, panic, and Central Serotonergic Inhibition. Stress: Neuroendocrinology and Neurobiology.

[51] Fujita T, Aoki N, Mori C, Fujita E, Matsushima T, Homma KJ, Yamaguchi S (2021). Serotonergic neurons in the Chick Brainstem Express various serotonin receptor subfamily genes. Front Physiol.

[52] Ley RE, Peterson DA, Gordon JI (2006). Ecological and evolutionary forces shaping microbial diversity in the human intestine. Cell.

[53] Stewart CJ, Ajami NJ, O’Brien JL, Hutchinson DS, Smith DP, Wong MC, Ross MC, Lloyd RE, Doddapaneni H, Metcalf GA (2018). Temporal development of the gut microbiome in early childhood from the TEDDY study. Nature.

[54] Li W, Nelson KE (2021). Microbial species that initially colonize the human gut at birth or in early childhood can stay in human body for lifetime. Microb Ecol.

[55] Gentile CL, Weir TL (2018). The gut microbiota at the intersection of diet and human health. Science.

[56] Leeming ER, Johnson AJ, Spector TD, Le Roy CI. Effect of Diet on the Gut Microbiota: Rethinking Intervention Duration. Nutrients 2019, 11. 10.3390/nu11122862 PMC6950569 31766592

[57] Rothschild D, Weissbrod O, Barkan E, Kurilshikov A, Korem T, Zeevi D, Costea PI, Godneva A, Kalka IN, Bar N (2018). Environment dominates over host genetics in shaping human gut microbiota. Nature.

[58] Boddy SL, Giovannelli I, Sassani M, Cooper-Knock J, Snyder MP, Segal E, Elinav E, Barker LA, Shaw PJ, McDermott CJ (2021). The gut microbiome: a key player in the complexity of amyotrophic lateral sclerosis (ALS). BMC Med.

[59] Chen YH, Bai J, Wu D, Yu SF, Qiang XL, Bai H, Wang HN, Peng ZW (2019). Association between fecal microbiota and generalized anxiety disorder: severity and early treatment response. J Affect Disord.

[60] Hills RD, Pontefract BA, Mishcon HR, Black CA, Sutton SC, Theberge CR (2019). Gut microbiome: profound implications for diet and disease. Nutrients.

[61] Precup G, Vodnar DC (2019). Gut Prevotella as a possible biomarker of diet and its eubiotic versus dysbiotic roles: a comprehensive literature review. Br J Nutr.

[62] Simpson HL, Campbell BJ (2015). Review article: dietary fibre-microbiota interactions. Aliment Pharmacol Ther.

[63] Castaner O, Goday A, Park YM, Lee SH, Magkos F, Shiow STE, Schroder H. (2018). The gut Microbiome Profile in obesity: a systematic review. Int J Endocrinol 2018, 4095789. 10.1155/2018/4095789 PMC5933040 29849617

[64] Sonnenburg ED, Sonnenburg JL (2019). The ancestral and industrialized gut microbiota and implications for human health. Nat Rev Microbiol.

[65] Merrill BD, Carter MM, Olm MR, Dahan D, Tripathi S, Spencer SP, Yu B, Jain S, Neff N, Jha AR et al. Ultra-deep Sequencing of Hadza Hunter-Gatherers Recovers Vanishing Gut Microbes. bioRxiv. 2022. 10.1016/j.cell.2023.05.046 PMC10330870 37348505

[66] Tang C, Ding R, Sun J, Liu J, Kan J, Jin C (2019). The impacts of natural polysaccharides on intestinal microbiota and immune responses - a review. Food Funct.

[67] Angelakis E, Bachar D, Yasir M, Musso D, Djossou F, Melenotte C, Robert C, Davoust B, Gaborit B, Azhar EI (2019). Comparison of the gut microbiota of obese individuals from different geographic origins. New Microbes New Infect.

[68] Anders JL, Mychajliw AM, Moustafa MAM, Mohamed WMA, Hayakawa T, Nakao R, Koizumi I (2022). Dietary niche breadth influences the effects of urbanization on the gut microbiota of sympatric rodents. Ecol Evol.

[69] Wong ML, Inserra A, Lewis MD, Mastronardi CA, Leong L, Choo J, Kentish S, Xie P, Morrison M, Wesselingh SL (2016). Inflammasome signaling affects anxiety- and depressive-like behavior and gut microbiome composition. Mol Psychiatry.

[70] Bosch JA, Nieuwdorp M, Zwinderman AH, Deschasaux M, Radjabzadeh D, Kraaij R, Davids M, de Rooij SR, Lok A (2022). The gut microbiota and depressive symptoms across ethnic groups. Nat Commun.

[71] Jin X, Zhang Y, Celniker SE, Xia Y, Mao JH, Snijders AM, Chang H (2021). Gut microbiome partially mediates and coordinates the effects of genetics on anxiety-like behavior in Collaborative Cross mice. Sci Rep.

[72] Qin J, Li R, Raes J, Arumugam M, Burgdorf KS, Manichanh C, Nielsen T, Pons N, Levenez F, Yamada T (2010). A human gut microbial gene catalogue established by metagenomic sequencing. Nature.

[73] Krajmalnik-Brown R, Ilhan ZE, Kang DW, DiBaise JK (2012). Effects of gut microbes on nutrient absorption and energy regulation. Nutr Clin Pract.

[74] Magne F, Gotteland M, Gauthier L, Zazueta A, Pesoa S, Navarrete P, Balamurugan R. The Firmicutes/Bacteroidetes ratio: a relevant marker of gut dysbiosis in obese patients? Nutrients 2020, 12. 10.3390/nu12051474 PMC7285218 32438689

[75] Walters WA, Xu Z, Knight R (2014). Meta-analyses of human gut microbes associated with obesity and IBD. FEBS Lett.

[76] Ozato N, Yamaguchi T, Mori K, Katashima M, Kumagai M, Murashita K, Katsuragi Y, Tamada Y, Kakuta M, Imoto S et al. Two Blautia Species Associated with visceral Fat Accumulation: a one-year longitudinal study. Biology (Basel) 2022, 11. 10.3390/biology11020318 PMC8869763 35205184

[77] Jumpertz R, Le DS, Turnbaugh PJ, Trinidad C, Bogardus C, Gordon JI, Krakoff J (2011). Energy-balance studies reveal associations between gut microbes, caloric load, and nutrient absorption in humans. Am J Clin Nutr.

[78] Jha AR, Davenport ER, Gautam Y, Bhandari D, Tandukar S, Ng KM, Fragiadakis GK, Holmes S, Gautam GP, Leach J. Gut microbiome transition across a lifestyle gradient in Himalaya. PLoS Biol 2018;16:e2005396. 10.1371/journal.pbio.2005396 PMC6237292 30439937

[79] Clarke SF, Murphy EF, Nilaweera K, Ross PR, Shanahan F, O’Toole PW, Cotter PD (2012). The gut microbiota and its relationship to diet and obesity: new insights. Gut Microbes.

[80] Commons KG, Connolley KR, Valentino RJ (2003). A neurochemically distinct dorsal raphe-limbic circuit with a potential role in affective disorders. Neuropsychopharmacology.

[81] Boldrini M, Underwood MD, Mann JJ, Arango V (2005). More tryptophan hydroxylase in the brainstem dorsal raphe nucleus in depressed suicides. Brain Res.

[82] Donner NC, Montoya CD, Lukkes JL, Lowry CA (2012). Chronic non-invasive corticosterone administration abolishes the diurnal pattern of tph2 expression. Psychoneuroendocrinology.

[83] Austin MC, Janosky JE, Murphy HA (2003). Increased corticotropin-releasing hormone immunoreactivity in monoamine-containing pontine nuclei of depressed suicide men. Mol Psychiatry.

[84] Bach-Mizrachi H, Underwood MD, Tin A, Ellis SP, Mann JJ, Arango V (2008). Elevated expression of tryptophan hydroxylase-2 mRNA at the neuronal level in the dorsal and median raphe nuclei of depressed suicides. Mol Psychiatry.

[85] Albert PR, Le Francois B, Vahid-Ansari F (2019). Genetic, epigenetic and posttranscriptional mechanisms for treatment of major depression: the 5-HT1A receptor gene as a paradigm. J Psychiatry Neurosci.

[86] Mekli K, Payton A, Miyajima F, Platt H, Thomas E, Downey D, Lloyd-Williams K, Chase D, Toth ZG, Elliott R (2011). The HTR1A and HTR1B receptor genes influence stress-related information processing. Eur Neuropsychopharmacol.

[87] Syed SA, Nemeroff CB (2017). Early life stress, Mood, and anxiety disorders. Chronic Stress (Thousand Oaks).

[88] Chirico MTT, Guedes MR, Vieira LG, Reis TO, Dos Santos AM, Souza ABF, Ribeiro IML, Noronha S, Nogueira KO, Oliveira LAM (2021). Lasting effects of ketamine and isoflurane administration on anxiety- and panic-like behavioral responses in Wistar rats. Life Sci.

[89] Alonso-Caraballo Y, Hodgson KJ, Morgan SA, Ferrario CR, Vollbrecht PJ (2019). Enhanced anxiety-like behavior emerges with weight gain in male and female obesity-susceptible rats. Behav Brain Res.

[90] Foroozan P, Koushkie Jahromi M, Nemati J, Sepehri H, Safari MA, Brand S. Probiotic Supplementation and High-Intensity Interval Training Modify Anxiety-Like Behaviors and Corticosterone in High-Fat Diet-Induced Obesity Mice. Nutrients 2021, 13. 10.3390/nu13061762 PMC8224367 34064242

[91] Ajayi AM, John KA, Emmanuel IB, Chidebe EO, Adedapo ADA (2021). High-fat diet-induced memory impairment and anxiety-like behavior in rats attenuated by peel extract of Ananas comosus fruit via atheroprotective, antioxidant and anti-inflammatory actions. Metabol Open.

[92] Li SW, Yu HR, Sheen JM, Tiao MM, Tain YL, Lin IC, Lin YJ, Chang KA, Tsai CC, Huang LT (2017). A maternal high-fat diet during pregnancy and lactation, in addition to a postnatal high-fat diet, leads to metabolic syndrome with spatial learning and memory deficits: beneficial effects of resveratrol. Oncotarget.

[93] Grosicki GJ, Durk RP, Bagley JR. Rapid gut microbiome changes in a world-class ultramarathon runner. Physiological Rep 2019;7:e14313. 10.14814/phy2.14313 PMC6928244 31872558

[94] Donner NC, Lowry CA (2013). Sex differences in anxiety and emotional behavior. Pflugers Arch.

[95] McLean CP, Asnaani A, Litz BT, Hofmann SG (2011). Gender differences in anxiety disorders: prevalence, course of illness, comorbidity and burden of illness. J Psychiatr Res.

