# Supplementary figures and images for: High-fat diet, microbiome-gut-brain axis signaling, and anxiety-like behavior in male rats
Source: Biol Res. 2024 May 6;57:23. doi: 10.1186/s40659-024-00505-1 (PMC11071217; doi:10.1186/s40659-024-00505-1)

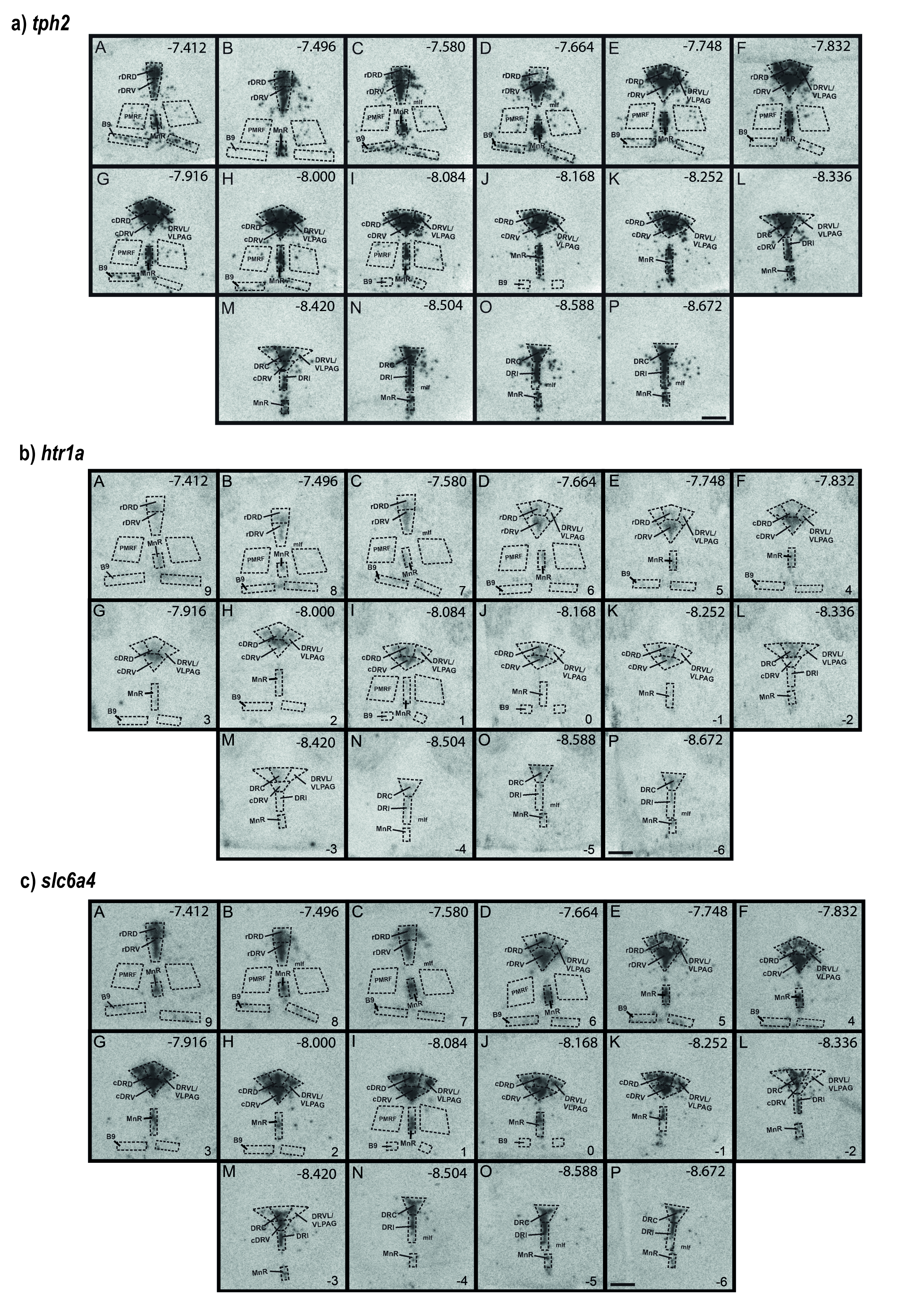

Supplement: Supplementary file 1 — Supplementary Material 1: Supplementary Fig. 1. Atlases of serotonergic gene expression. (a) Atlas of rat tryptophan hydroxylase 2 (tph2) mRNA expression, (b) atlas of rat 5-HT1A receptor (htr1a) mRNA expression, and (c) atlas of rat serotonin transporter (slc6a4) mRNA expression in the midbrain and pontine raphe complex (84 μm intervals) used for analysis of subregions of the dorsal raphe nucleus (DR), median raphe nucleus (MnR), pontomesencephalic reticular formation (PMRF) and B9 serotonergic cell group (B9) with a high level of neuroanatomical resolution. Photographs are autoradiographic images of tph2, htr1a, and slc6a4 mRNA expression. The levels chosen for analysis ranged from (A) − 7.412 mm bregma through (P) − 8.672 mm bregma. Dotted lines delineate different subdivisions of the DR analyzed in this study, based on a stereotaxic atlas of the rat brain [39]. Abbreviations: B9, supralemniscal serotonergic cell group; MnR, median raphe nucleus; DRC, dorsal raphe nucleus, caudal part; DRD, dorsal raphe nucleus, dorsal part; DRI, dorsal raphe nucleus, interfascicular part; DRV, dorsal raphe nucleus, ventral part; DRVL, dorsal raphe nucleus, ventrolateral part; PMRF, pontomesencephalic reticular formation; VLPAG, ventrolateral periaqueductal gray. Numbers in the upper right of each panel indicate the rostrocaudal coordinates relative to bregma (in mm). Numbers in the lower right of each panel indicate the rostrocaudal level of each DR. Scale bar, 1 mm. The rostrocaudal levels and matrices defining subregions of the DR that were defined using tph2 autoradiograms were also used in the analysis of htr1a and slc6a4 to ensure sampling of the same anatomical regions for each gene [file 40659_2024_505_MOESM1_ESM.tif]

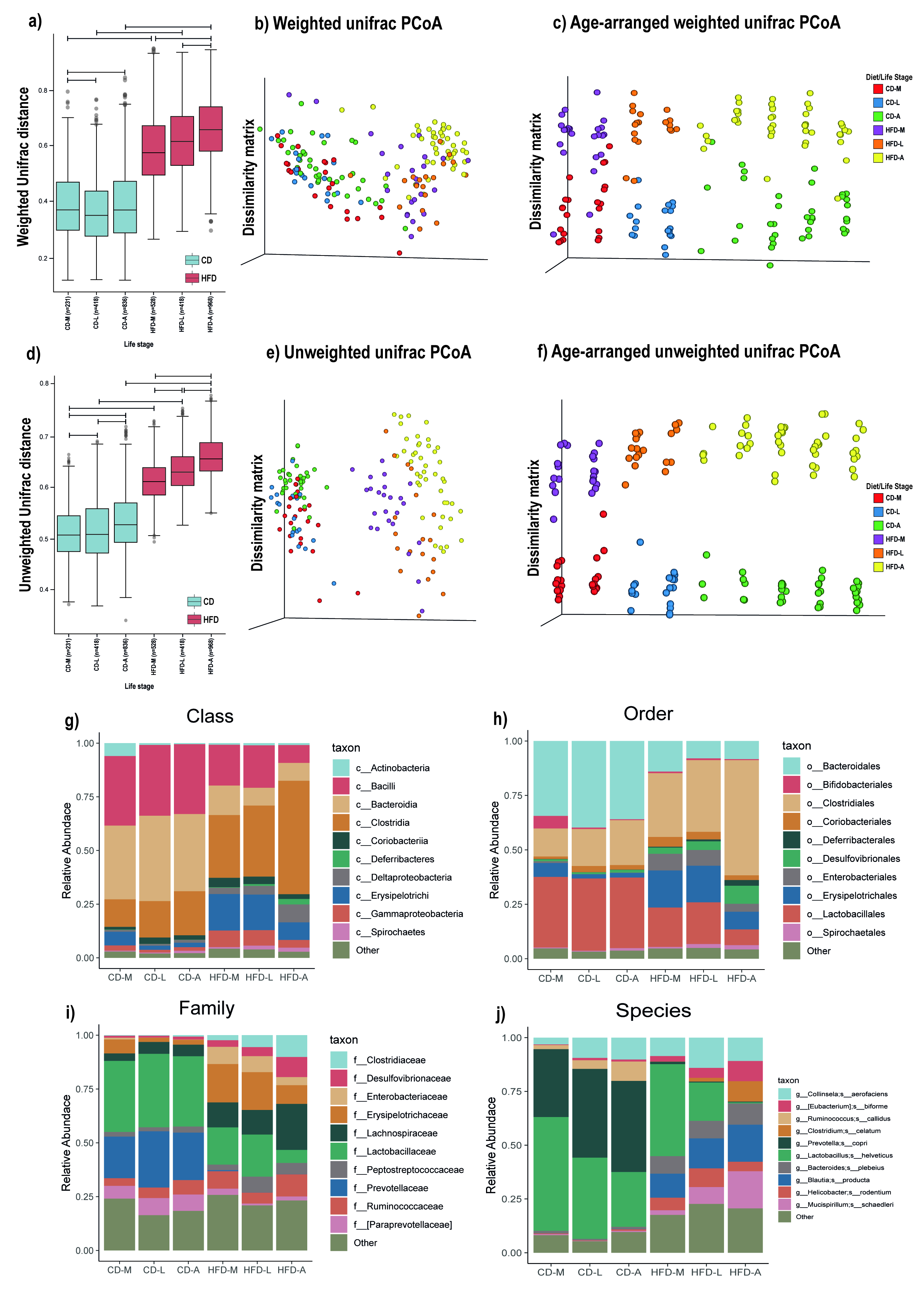

Supplement: Supplementary file 2 — Supplementary Material 2: Supplementary Fig. 2. Effects of high-fat diet (HFD) on alpha diversity, beta diversity, and community composition of the gut microbiome across mid-adolescence, late adolescence, and adulthood. (a) Beta diversity distance comparison plot with box plots illustrating distances within samples and between groups using Weighted UniFrac distance, (b) Weighted UniFrac PCoA plot of dissimilarity matrix, and (c) age-arranged weighted UniFrac PCoA plot of dissimilarity matrix. (d) Beta diversity distance comparison plot with box plots illustrating distances within samples and between groups using Unweighted UniFrac distance, (e) Unweighted UniFrac PCoA plot of dissimilarity matrix, and (f) age-arranged Unweighted UniFrac PCoA plot of dissimilarity matrix. Top ten taxa with highest relative abundances illustrated by stacked vertical bar charts for (g) class, (h) order, (i) family, and (j) genus. Data are expressed as boxplots, where bottom and tops of boxes indicate the first and third quartiles, respectively; whiskers indicate the interquartile range (IQR) beyond the upper and lower quartiles. PERMANOVA pairwise test. Abbreviations: CD-A, control diet group/adulthood; CD-L, control diet group/late adolescence; CD-M, control diet group/middle adolescence; HFD-A, high-fat diet group/adulthood; HFD-L, high-fat diet group/late adolescence; HFD-M, high-fat diet group/middle adolescence [file 40659_2024_505_MOESM2_ESM.tif]

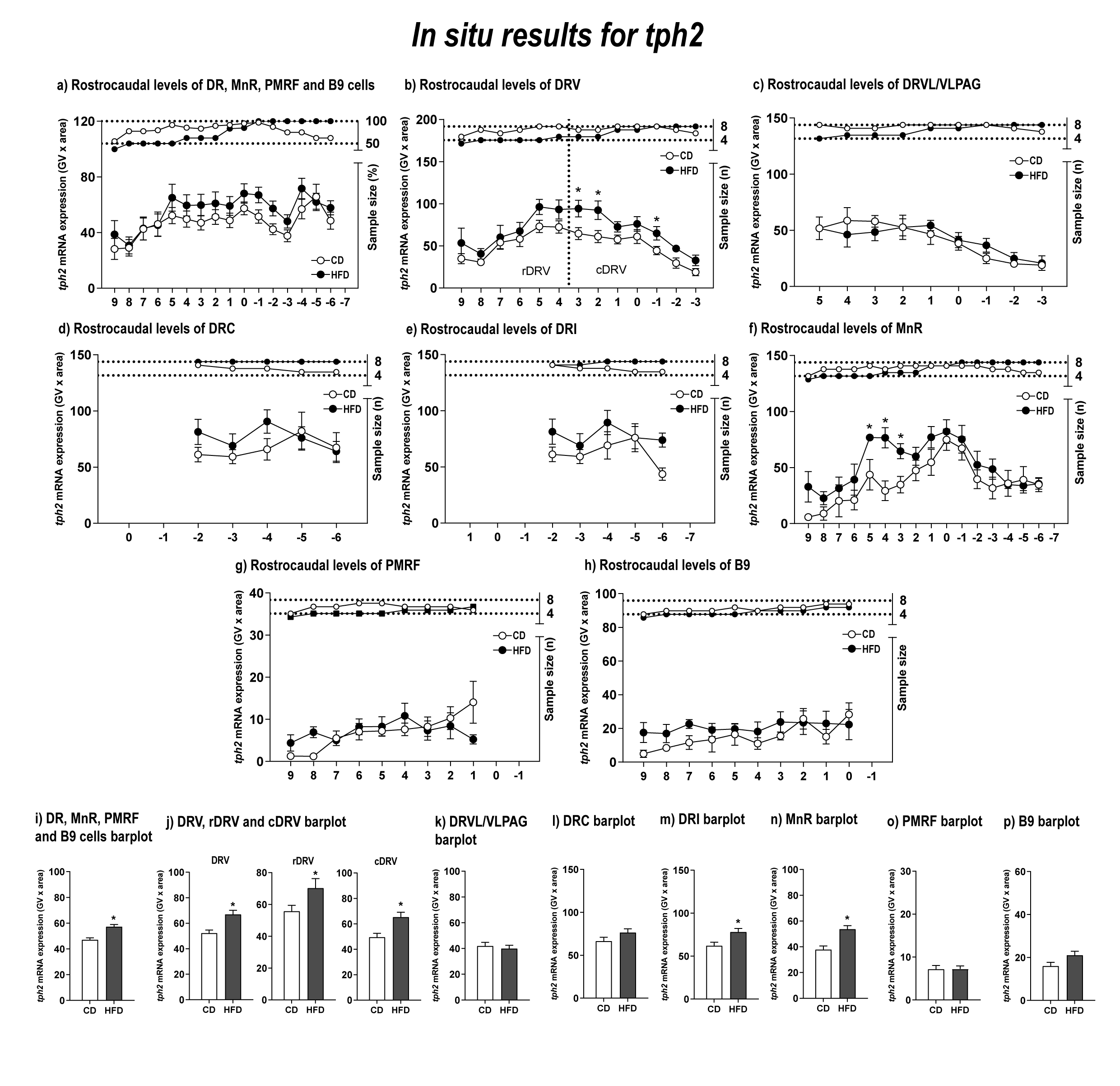

Supplement: Supplementary file 3 — Supplementary Material 3: Supplementary Fig. 3. Effects of nine weeks of a control diet (CD) or high-fat diet (HFD) protocol on tph2 mRNA expression in subdivisions of the dorsal raphe nucleus (DR), median raphe nucleus (MnR), pontomesencephalic reticular formation (PMRF), and B9 serotonergic cell group. Each graph represents the means ± SEMs of tph2 mRNA expression levels at specific rostrocaudal levels or within subregions. Graphs illustrate tph2 mRNA expression in the (a) total DR, MnR, PMRF, and B9 supralemniscal serotonergic cell group, (b) dorsal raphe nucleus, ventral part (DRV), including the rostral (rDRV) and caudal (cDRV) aspects, (c) dorsal raphe nucleus, ventrolateral part (DRVL)/ventrolateral periaqueductal gray (VLPAG), (d) dorsal raphe nucleus, caudal part (DRC), (e) dorsal raphe nucleus, interfascicular part (DRI), (f) MnR, (g) PMRF, and (h) B9 supralemniscal serotonergic cell group. Further compiled levels are shown for (i) total rostrocaudal levels of DR, MnR, PMRF, and B9 serotonergic cell group bar plot, (j) DRV, rDRV and cDRV bar plots, (k) DRVL/VLPAG bar plots, (l) DRC bar plot, (m) DRI bar plot, (n) MnR bar plot, (o) PMRF bar plot, and (p) B9 supralemniscal serotonergic cell group bar plot. *p < 0.05 versus CD at the same rostrocaudal level (a-h); versus CD based on compiled tph2 mRNA expression across rostrocaudal levels (i-p), white circles/bars represent CD group, and black circles/bars represent HFD group. Rostrocaudal levels 9 = − 7.412 mm, 8 = − 7.496 mm, 7 = − 7.580 mm, 6 = − 7.664 mm, 5 = − 7.748 mm, 4 = − 7.832 mm, 3 = − 7.916 mm, 2 = − 8.00 mm, 1 = − 8.084 mm, 0 = − 8.168 mm, − 1 = − 8.252 mm, − 2 = − 8.336 mm, − 3 = − 8.420 mm, − 4 = − 8.504 mm, − 5 = − 8.588 mm, and − 6 = − 8.672 mm. Sample sizes for each treatment group at each rostrocaudal level of analysis are shown on the upper section of the panel (a-h). Abbreviations: CD, control diet; HFD, high-fat diet [file 40659_2024_505_MOESM3_ESM.tif]

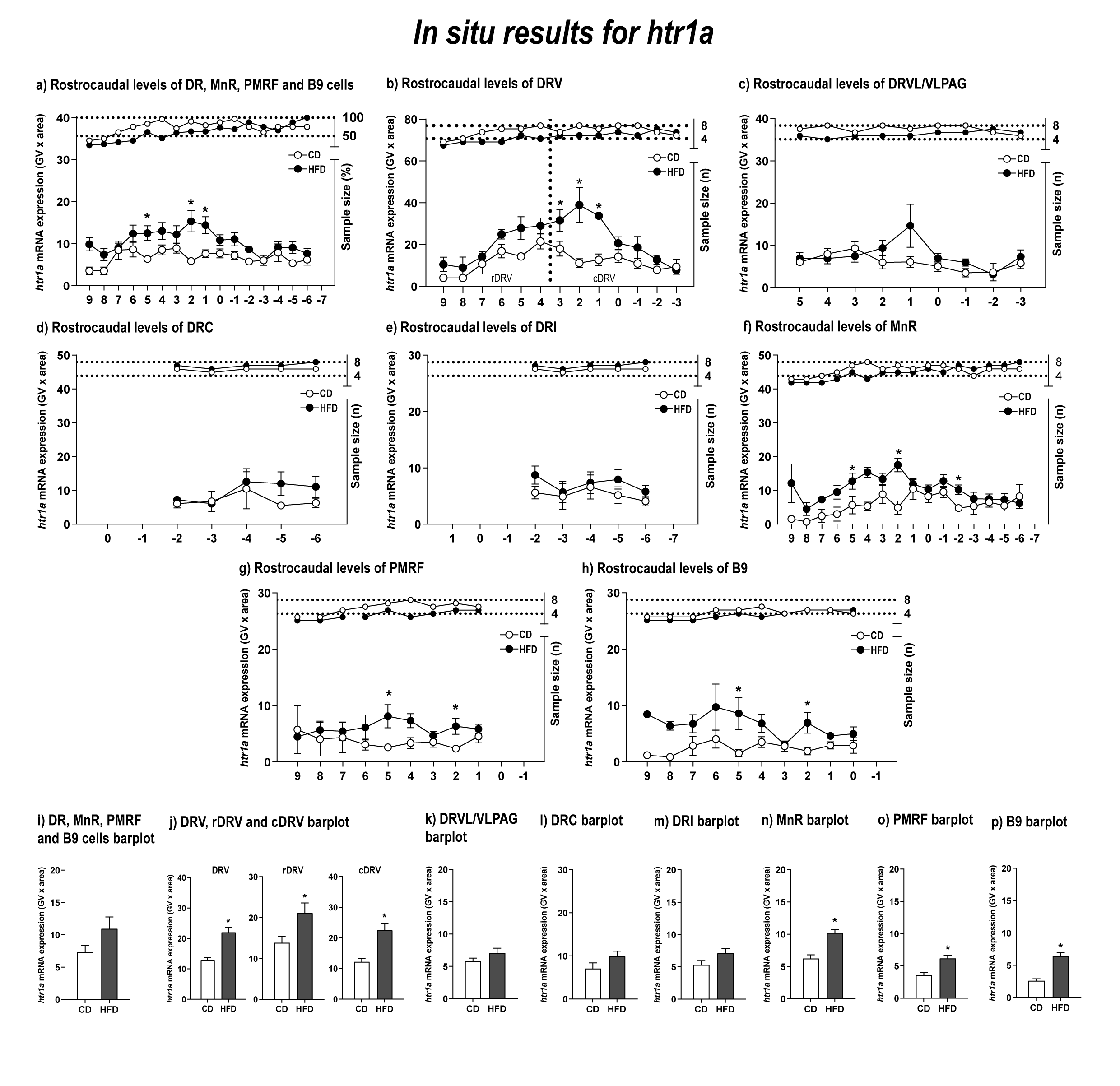

Supplement: Supplementary file 4 — Supplementary Material 4: Supplementary Fig. 4. Effects of nine weeks of a control diet (CD) or high-fat diet (HFD) protocol on htr1a mRNA expression in subdivisions of the dorsal raphe nucleus (DR), median raphe nucleus (MnR), pontomesencephalic reticular formation (PMRF), and B9 serotonergic cell group. Each graph represents the means ± SEMs of htr1a mRNA expression levels at specific rostrocaudal levels or within subregions. Graphs illustrate htr1a mRNA expression in the (a) total combined expression in dorsal raphe nucleus (DR), median raphe nucleus (MnR), pontomesencephalic reticular formation (PMRF), and B9 supralemniscal serotonergic cell group, (b) dorsal raphe nucleus, ventral part (DRV), including the rostral (rDRV) and caudal (cDRV) aspects, (c) dorsal raphe nucleus, ventrolateral part (DRVL)/ventrolateral periaqueductal gray (VLPAG), (d) dorsal raphe nucleus, caudal part (DRC), (e) dorsal raphe nucleus, interfascicular part (DRI), (f) MnR, (g) PMRF, and (h) B9 supralemniscal serotonergic cell group. Further compiled levels are shown for (i) total rostrocaudal levels of DR bar plot, (j) DRV, rDRV and cDRV bar plots, (k) DRVL/VLPAG bar plots, (l) DRC bar plot, (m) DRI bar plot, (n) MnR bar plot, (o) PMRF bar plot, and (p) B9 supralemniscal serotonergic cell group bar plot. *p < 0.05, versus CD at the same rostrocaudal level (a-h); versus CD based on compiled htr1a mRNA expression across rostrocaudal levels (i-p), white circles/bars represent CD group, and black circles/bars represent HFD group. Rostrocaudal levels 9 = − 7.412 mm, 8 = − 7.496 mm, 7 = − 7.580 mm, 6 = − 7.664 mm, 5 = − 7.748 mm, 4 = − 7.832 mm, 3 = − 7.916 mm, 2 = − 8.00 mm, 1 = − 8.084 mm, 0 = − 8.168 mm, − 1 = − 8.252 mm, − 2 = − 8.336 mm, − 3 = − 8.420 mm, − 4 = − 8.504 mm, − 5 = − 8.588 mm, and − 6 = − 8.672 mm. Sample sizes for each treatment group at each rostrocaudal level of analysis are shown on the upper section of the panel (a-h). Abbreviations: CD, control diet; HFD, high-fat diet [file 40659_2024_505_MOESM4_ESM.tif]

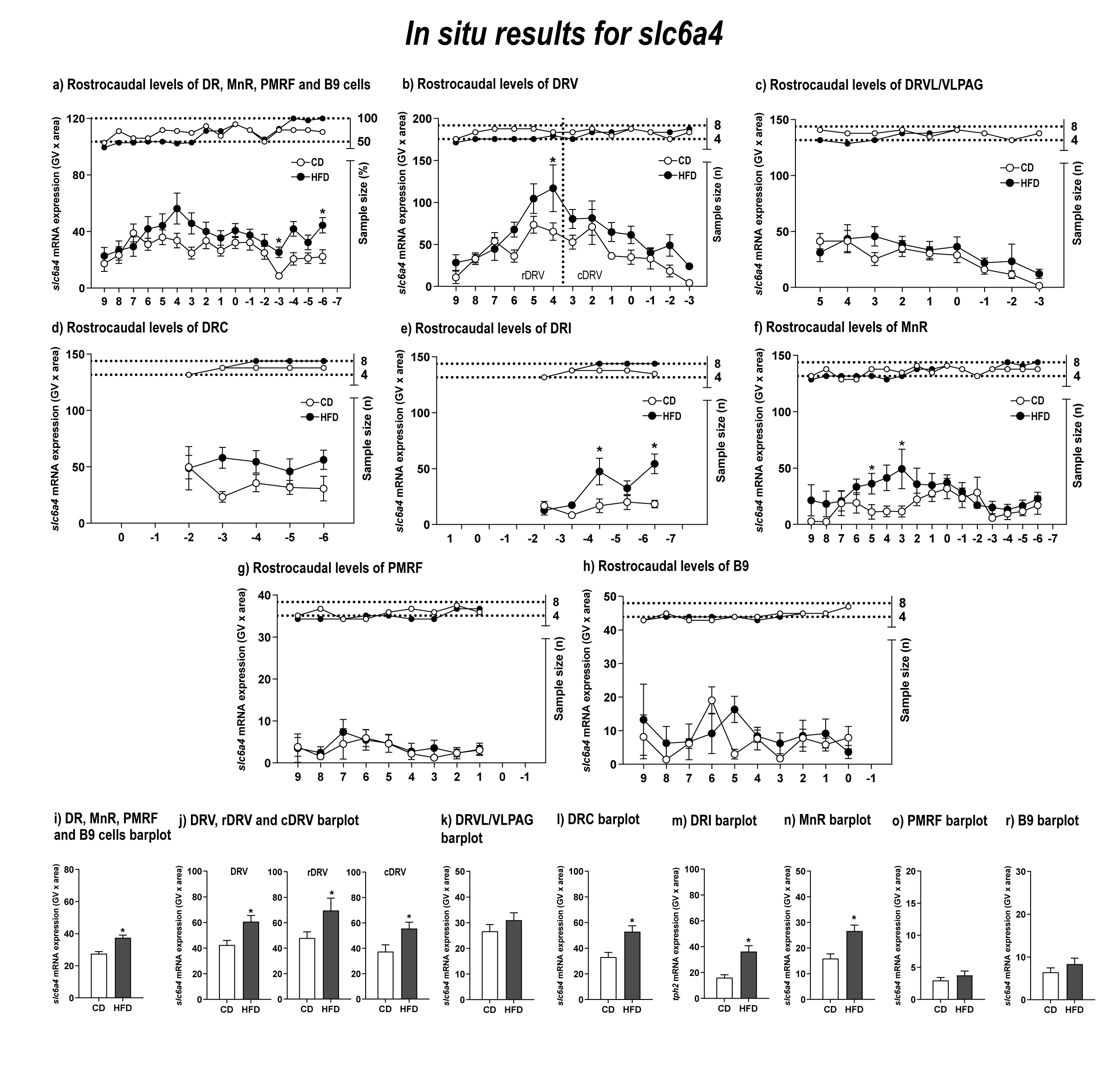

Supplement: Supplementary file 5 — Supplementary Material 5: Supplementary Fig. 5. Effects of nine weeks of a control diet (CD) or high-fat diet (HFD) protocol on slc6a4 mRNA expression in subdivisions of the dorsal raphe nucleus (DR), median raphe nucleus (MnR), pontomesencephalic reticular formation (PMRF), and B9 supralemniscal serotonergic cell group. Each graph represents the means ± SEMs of slc6a4 mRNA expression levels at specific rostrocaudal levels or within subregions. Graphs illustrate slc6a4 mRNA expression in the (a) total combined expression in the DR, MnR, PMRF, and B9 supraleminiscal serotonergic cell group, (b) dorsal raphe nucleus, ventral part (DRV), including the rostral (rDRV) and caudal (cDRV) aspects, (c) dorsal raphe nucleus, ventrolateral part (DRVL)/ventrolateral periaqueductal gray (VLPAG), (d) dorsal raphe nucleus, caudal part (DRC), (e) dorsal raphe nucleus, interfascicular part (DRI), (f) MnR, (g) PMRF, and (h) B9 supralemniscal serotonergic cell group. Further compiled levels are shown for (i) total rostrocaudal levels of DR bar plot, (j) DRV, rDRV and cDRV bar plots, (k) DRVL/VLPAG bar plots, (l) DRC bar plot, (m) DRI bar plot, (n) MnR bar plot, (o) PMRF bar plot, and (p) B9 supralemniscal serotonergic cell group bar plot. *p < 0.05 versus CD at the same rostrocaudal level (a-h); versus CD based on compiled slc6a4 mRNA expression across rostrocaudal levels (i-p), white circles/bars represent CD group, and black circles/bars represent HFD group. Rostrocaudal levels 9 = − 7.412 mm, 8 = − 7.496 mm, 7 = − 7.580 mm, 6 = − 7.664 mm, 5 = − 7.748 mm, 4 = − 7.832 mm, 3 = − 7.916 mm, 2 = − 8.00 mm, 1 = − 8.084 mm, 0 = − 8.168 mm, − 1 = − 8.252 mm, − 2 = − 8.336 mm, − 3 = − 8.420 mm, − 4 = − 8.504 mm, − 5 = − 8.588 mm, and − 6 = − 8.672 mm. Sample sizes for each treatment group at each rostrocaudal level of analysis are shown on the upper section of the panel (a-h). Abbreviations: CD, control diet; HFD, high-fat diet [file 40659_2024_505_MOESM5_ESM.tif]

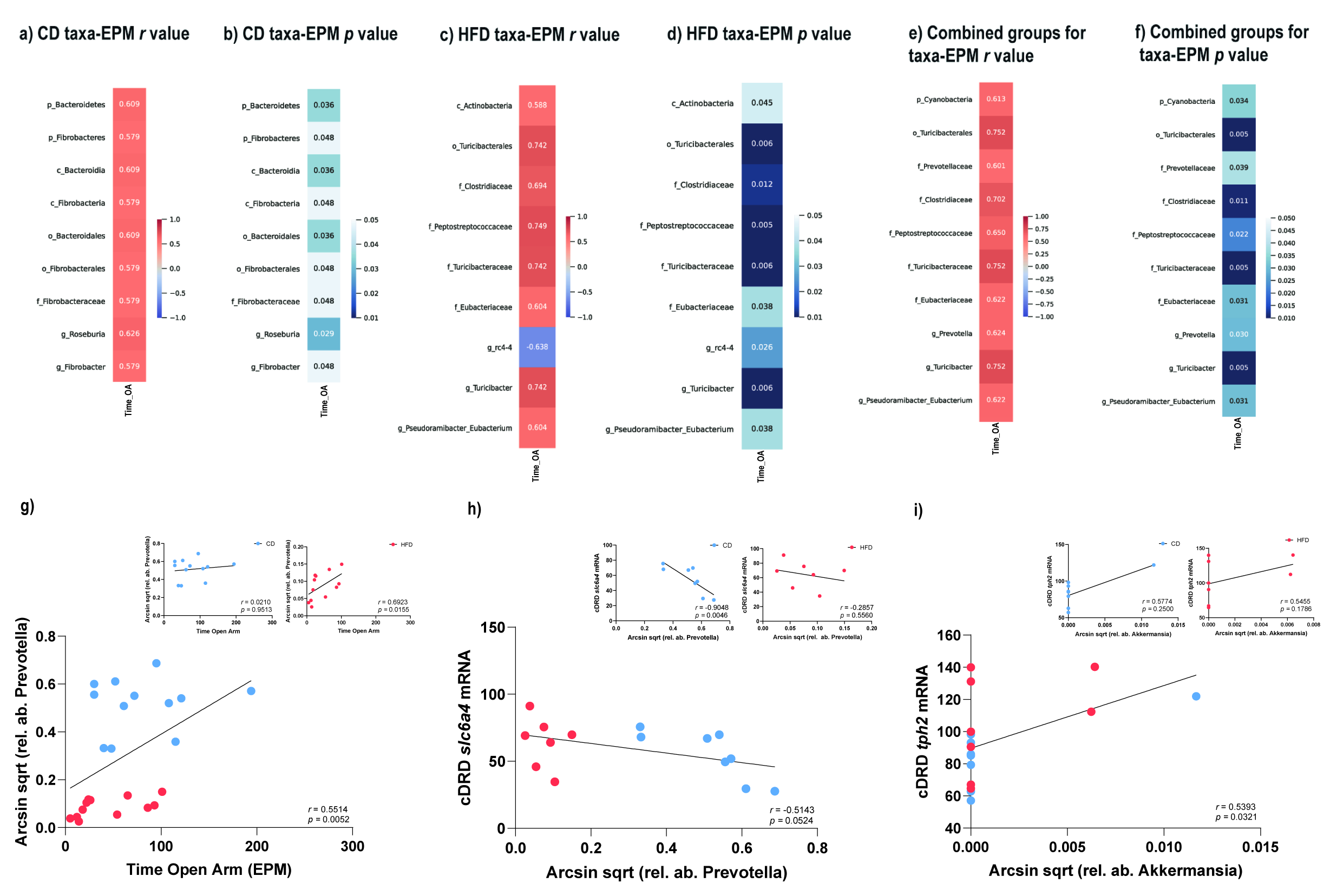

Supplement: Supplementary file 6 — Supplementary Material 6: Supplementary Fig. 6. Analysis for rats treated with control diet (CD) and high-fat diet (HFD) to reveal correlations of the time spent in the open-arm of the elevated plus-maze versus relative abundance of specific taxa. Each graph represents Spearman’s correlation coefficient of time in the open arm versus relative abundance of specific taxa, and r and p values are shown on the panels according to the analysis of time spent in the open-arm of the elevated plus-maze x taxa, separately for CD and HFD. (a-b) CD taxa x open arm correlation, (c-d) HFD taxa x open arm correlation. (e-f) CD and HFD taxa combined x open arm correlation. (g) Analysis of correlation between time in the open arm versus Prevotella relative abundance. (h) slc6a4 gene expression versus Prevotella. For r values, blue represents negative correlations and red positive correlations; for p values heatmap, blue scale represents variations within 0.05 and 0.01. Abbreviations: CD, control diet; HFD, high-fat diet [file 40659_2024_505_MOESM6_ESM.tif]
