# Supplementary material for: High-fat diet, microbiome-gut-brain axis signaling, and anxiety-like behavior in male rats
Source: Biol Res. 2024 May 6;57:23. doi: 10.1186/s40659-024-00505-1 (PMC11071217; doi:10.1186/s40659-024-00505-1)
Supplement: Supplementary file 8 — Supplementary Material 8 [file 40659_2024_505_MOESM8_ESM.docx]

**Supplementary Material**

**Supplementary Methods**

**1. Diet composition used for treatment**

**Table S1.** High-fat diet (45% kcal/g of fat) and a standard control diet (11% kcal/g fat) chemical composition.

| **High-fat diet 45%**  **kcal/w fat (HFD)*** | **g/Kg** | **kcal/g** | **Control diet 11% kcal/w fat (CD)**** | **g/Kg** | **kcal/g** |
| --- | --- | --- | --- | --- | --- |
| Choline | 2.5 | 0 | Protein | 220 | 880 |
| Mix of vitamins (AIN93) | 10 | 0 | Cellulose | 80 | 0 |
| Mix of minerals (AIN93) | 35 | 0 | Lipids | 40 | 360 |
| Cellulose | 50 | 0 | Carbohydrates*** | 560 | 2240 |
| Maltodextrin | 115 | 460 | Vitamins and Minerals | 50 | 0 |
| Starch | 127.5 | 510 | Humidity | 50 | 0 |
| Sucrose | 200 | 800 | - | - | - |
| Casein | 220 | 880 | - | - | - |
| Lard | 200 | 1800 | - | - | - |
| Soy Oil | 40 | 360 | - | - | - |
| **Total** | 1000 | 4810 | **Total** | 1000 | 3480 |

*High-fat diet (PRAG Soluções Comércio e Serviços em Biociências®; hyperlipidic diet 45% fat kcal/kg); **Standard control diet (NUVILAB CR1-NUVITAL®; standard diet 11% fat kcal/kg); ***Carbohydrates were mostly composed of whole ground corn, soybean, and wheat bran mix.

**2. Adipose tissue and weight composition from rats after 9 weeks**

**Table S2.** HFD effects on body mass composition, compared to CD adult male Wistar rats after 9 weeks of diet protocol.

| **Parameters (g)** | **Control diet (*N* = 12)** | | **High fat diet (*N* = 12)** | | ***p-value*** |
| --- | --- | --- | --- | --- | --- |
| Final body weight | | 380.1 ± 6.5 | 421.1 ± 11.6^#^ | *p* = 0.0054 | |
| Weight gain | | 281.5 ± 7.4 | 322.6 ± 11.1^#^ | *p* = 0.0055 | |
| Epididymal fat | | 3.1 ± 0.3 | 7.4 ± 0.8^#^ | *p* < 0.0001 | |
| Retroperitoneal fat | | 3.9 ± 0.4 | 9.0 ± 0.8^#^ | *p* < 0.0001 | |
| Inguinal fat | | 6.7 ± 0.4 | 11.8 ± 0.9^#^ | *p* < 0.0001 | |
| Adiposity index | | 3.6 ± 0.3 | 6.7 ± 0.6^#^ | *p* < 0.0001 | |

^+^sum of visceral fat (epididymal + retroperitoneal + inguinal) (g)/final body weight (g) x 100; ^#^Data are expressed as means ± SEMs; *p* < 0.05 between groups; unpaired Student's *t*-test.

**3. EPM parameters used for behavioral assessment**

**Table S3.** EPM parameters for the number, percent of time, and entries in both open and enclosed arms of the apparatus.

| **EPM parameters** | **Control Group** | **HFD group** | ***p* value** |
| --- | --- | --- | --- |
| Entries in open arms (n) | 4.66 ± 0.48 | 3.08 ± 0.52 | 0.0440 |
| Entries in closed arms (n) | 4.16 ± 0.66 | 4.91 ± 0.58 | 0.5550 |
| Percent entries in open arms (%) | 55.62 ± 3.95 | 36.22 ± 3.28 | 0.0013 |
| Time spent in open arms (s) | 80.50 ± 13.96 | 43.33 ± 10.03 | 0.0232 |
| Time spent in closed arms (s) | 208.90 ± 14.17 | 215.40 ± 14.34 | 0.7986 |
| Percent time spent in open arms (%) | 26.83 ± 4.65 | 14.44 ± 3.34 | 0.0232 |

Values represented as means ± SEMs, considering *p* < 0.05. n = number; s = seconds.

**4. Riboprobe preparation**

Riboprobes were radiolabeled via *in vitro* transcription, incorporating [^35^S]-UTP. Briefly, a nucleotide mixes of ATP, CTP, GTP (1.1 μl of 10 mM each), and UTP (1.1 μl of 0.2 mM) was added to 2.2 μl 10x transcription buffer (Promega, Madison, WI, USA, cat. no. FP021) and 0.22 μl 1 M dithiothreitol (DTT). From this solution, 6.2 μl was withdrawn and added to 1 μl (2 μg) cut DNA (antisense or sense), 1.8 μl RNase inhibitor (Invitrogen, RNaseOUT, cat. no. 100000840), 4 μl [^35^S]-UTP (1250 Ci/mmol; PerkinElmer, Boston, MA, USA, cat. no. NEG039H001MC), and 2 *μ*l of the appropriate RNA polymerase (T3 for antisense (cat. no. P208C) and T7 for sense (cat. no. P207B Promega). The mixture was incubated at 37 °C for 1 h. The template DNA was then removed by digestion with 0.75 *μ*l RNase-free DNase I (Promega, RQ1 DNase Stop Solution, cat. no. M199A) for 15 min at 37 °C. The probe was precipitated by ethanol in the presence of 0.5 mg/ml glycogen carrier and re-dissolved in 100 μl water. Probe activity (1 μl) was counted in 5 ml scintillation fluid (Beckman Coulter, Ready Safe, Fullerton, CA, USA, cat. no. p/n484013-ae) with a beta counter (Beckman LS 3801, ser. no. 7013835) and was typically 3–4X10^6^ cpm (Lieb et al. 2019).

**5. Supplementary statistical analysis for *in situ* hybridization histochemistry**

For analysis of *in situ* hybridization histochemistry data, statistical analyses were conducted using the software package IBM Statistical Package for the Social Sciences (version 25.0, SPSS Inc., Chicago, IL, USA). Mean (corrected for background) gray value x area values for each rostrocaudal level of each DR, MnR, PMRF, and B9 cells subdivision for each rat were generated for *tph2*, *htr1a*, and s*lc6a4* gene expression. Extreme outliers were identified using Grubbs' test for single outliers, using two-sided α = 0.05 (Grubbs, 1969). All tests were two-tailed with a level of significance of *p* < 0.05. We used a linear mixed effects model (LMM), modeling mRNA expression ([mean gray values (corrected for background)] x area) by diet (CD or HFD) at each rostrocaudal level (units of mm bregma and included the values: −7.412, −7.496, −7.580, −7.664, −7.748, −7.832, −7.916, −8.000, −8.084, −8.168, −8.252, −8.336, −8.420, −8.504, −8.588, and −8.672 ), within each subregion (DRD [caudal and rostral aspects], DRV [caudal and rostral aspects], DRVL/VLPAG, DRC, DRI, MnR, PMRF and B9 cells).

In addition, 16 covariate structures correlations were used, including: ARMA (1,1); compound symmetry; correlation compound symmetry; diagonal; first-order analytic; first-order ante-dependence; first-order autoregressive; first-order factor analytic; heterogeneous compound symmetry; heterogeneous first-order autoregressive; heterogeneous toeplitz; Huynh-Feldt; identity; toeplitz; unstructured; and unstructured. To exemplify the SPSS syntax for the level one model was as follows:

MIXED mRNA BY Diet RC_LEVEL subregion

/CRITERIA=CIN(95) MXITER(100) MXSTEP(10) SCORING(1) SINGULAR(0.000000000001) HCONVERGE(0,

ABSOLUTE) LCONVERGE(0, ABSOLUTE) PCONVERGE(0.000001, ABSOLUTE)

/FIXED=Diet RC_LEVEL subregion Diet*RC_LEVEL Diet*subregion RC_LEVEL(subregion)

Diet*RC_LEVEL(subregion) | SSTYPE(3)

/METHOD=REML

/REPEATED=RC_LEVEL | SUBJECT(Animal*subregion) COVTYPE(AD1)

/SAVE=PRED RESID.

The best fit model based on the covariance structures surveyed were compared, and the lowest −2 log-likelihood of each gene were chosen. After the overall analysis, if any a main effect interaction or any other factor reached *p* < 0.05, a subsequent LMM analysis were applied for each individual subregion of the DR, PMRF, MnR and B9 cells, using the modeling for mRNA expression and the 16 covariate structures listed. The SPSS syntax for the level two model was as follows:

MIXED rDRD BY Diet RC_level

/CRITERIA=CIN(95) MXITER(100) MXSTEP(10) SCORING(1) SINGULAR(0.000000000001) HCONVERGE(0,

ABSOLUTE) LCONVERGE(0, ABSOLUTE) PCONVERGE(0.000001, ABSOLUTE)

/FIXED=Diet RC_level Diet*RC_level | SSTYPE(3)

/METHOD=REML

/REPEATED=RC_level | SUBJECT(Animal) COVTYPE(AD1).

After fitting the best model within the survey of covariance structures minimized for −2 log-likelihood, the main effect interaction that reached the *p* < 0.05 was tested with the post-hoc pairwise comparisons using Fisher’s least significant difference (LSD). The following SPSS syntax for this step is as follows:

UNIANOVA rDRD BY Diet_RC_level

/METHOD=SSTYPE(3)

/INTERCEPT=INCLUDE

/POSTHOC=Diet_RC_level(LSD)

/CRITERIA=ALPHA(0.05)

/DESIGN=Diet_RC_level.

Analysis of the mean compiled values of the mRNA gene expression across the rostrocaudal extent of each subregion was performed (see Fig. 1 a-i, and supplementary Fig. S1, S2 and S3). *In situ* hybridization histochemistry analysis was performed when the rostrocaudal extent of subregions of the DR, MnR, PMRF and B9 cells had sample sizes over 50% of the full sample, and post hoc analyses were obtained only when overall and follow-up LMM yielded significant outcomes. Data were presented as means ± standard error of the means (SEM) at a two-tailed significance of *p* < 0.05.

**5.1. Output for *in situ* hybridization for *tph2* mRNA expression**

**Table S4. Linear mixed model analysis for *tph2***

| **Model and source** | | **Test statistic** | ***p*-value** |
| --- | --- | --- | --- |
| **Overall Analysis (covariance structure)** | |  |  |
| **Entire dataset - First-order ante-dependence** | |  |  |
|  | Diet (CD vs. HFD) | *F*_(1,165.35)_ = 19.09 | <0.001 |
|  | Rostrocaudal level | *F*_(15,121.05)_ = 14.83 | <0.001 |
|  | Raphe subregion | *F*_(9,98.76)_ = 67.72 | <0.001 |
|  | Diet *Rostrocaudal level | *F*_(15,121.05)_ = 1.29 | 0.214 |
|  | Diet *Raphe subregion | *F*_(9,98.76)_ = 1.85 | 0.067 |
|  | Rostrocaudal level (Raphe subregion) | *F*_(52,140.7)_ = 10.70 | 0.001 |
|  | Treatment *Rostrocaudal level (Raphe subregion) | *F*_(52,140.7)_ = 1.20 | 0.229 |
|  |  |  |  |
| **Subregional Analyses (covariance structure)** | |  |  |
| **rDRD (First-Order Ante-Dependence)** | | |  |
|  | Treatment (CD vs. HFD) | *F*_(1,10.38)_ < 0.01 | 0.984 |
|  | Rostrocaudal level | *F*_(5,14.58)_ = 5.33 | 0.006 |
|  | Treatment *Rostrocaudal Level | *F*_(5,14.58)_ = 1.96 | 0.145 |
| **cDRD (Unstructured)** | | |  |
|  | Treatment (CD vs. HFD) | *F*_(1,12.61)_ = 5.90 | 0.031 |
|  | Rostrocaudal level | *F*_(4,10.42)_ = 21.40 | <0.001 |
|  | Treatment *Rostrocaudal Level | *F*_(4,10.42)_ = 2.08 | 0.156 |
| **rDRV (First-Order Ante-Dependence)** | | |  |
|  | Treatment (CD vs. HFD) | *F*_(1,12.25)_ = 3.93 | 0.070 |
|  | Rostrocaudal level | *F*_(1,14,67)_ = 17.82 | <0.001 |
|  | Treatment *Rostrocaudal Level | *F*_(1,14.67)_ = 0.26 | 0.929 |
| **cDRV (Unstructured)** | | |  |
|  | Treatment (CD vs. HFD) | *F*_(1,12.13)_ = 7.25 | 0.019 |
|  | Rostrocaudal level | *F*_(6,11.90)_ = 20.93 | <0.001 |
|  | Treatment *Rostrocaudal Level | *F*_(6,11.90)_ = 0.64 | 0.694 |
| **DRVL/VLPAG (First-Order Ante-Dependence)** | |  |  |
|  | Treatment (CD vs. HFD) | *F*_(1,15.68)_ = 0.27 | 0.872 |
|  | Rostrocaudal level | *F*_(8,26.68)_ = 9.36 | <0.001 |
|  | Treatment *Rostrocaudal Level | *F*_(8,26.68)_ = 1.25 | 0.307 |
| **DRC (Unstructured)** | |  |  |
|  | Treatment (CD vs. HFD) | *F*_(1,13.36)_ = 0.75 | 0.401 |
|  | Rostrocaudal level | *F*_(4,11.97)_ = 2.84 | 0.072 |
|  | Treatment *Rostrocaudal Level | *F*_(4,11.97)_ = 0.93 | 0.477 |
| **DRI (Unstructured)** | |  |  |
|  | Treatment (CD vs. HFD) | *F*_(1,13.35)_ = 2,44 | 0.141 |
|  | Rostrocaudal level | *F*_(4,12.61)_ = 5,24 | 0.010 |
|  | Treatment *Rostrocaudal Level | *F*_(4,12.61)_ = 2.79 | 0.073 |
| **MnR (First-Order Ante-Dependence)** | | |  |
|  | Treatment (CD vs. HFD) | *F*_(1,24.29)_ = 6.22 | 0.020 |
|  | Rostrocaudal level | *F*_(15,26.84)_ = 9.49 | <0.001 |
|  | Treatment *Rostrocaudal Level | *F*_(15,26.84)_ = 1.06 | 0.429 |
| **PMRF (First-Order Ante-Dependence)** | | |  |
|  | Diet (CD vs HFD) | *F*_(1,15.28)_ = 0.17 | 0.682 |
|  | Rostrocaudal level | *F*_(8,13.49)_ = 4.82 | 0.006 |
|  | Treatment *Rostrocaudal Level | *F*_(8,13.49)_ = 2.45 | 0.070 |
| **B9 (First-Order Ante-Dependence)** | | |  |
|  | Treatment (CD vs. HFD) | *F*_(1,19.53)_ = 2.85 | 0.107 |
|  | Rostrocaudal level | *F*_(9,12.51)_ = 1.52 | 0.240 |
|  | Treatment *Rostrocaudal Level | *F*_(9,12.51)_ = 0.82 | 0.608 |

**5.2. Output for *in situ* hybridization for *htr1a* mRNA expression**

**Table S5. Linear mixed model analysis for *htr1a***

| **Model and source** | | **Test statistic** | ***p*-value** |
| --- | --- | --- | --- |
| **Overall Analysis (covariance structure)** | |  |  |
| **Entire dataset - First-order ante-dependence** | |  |  |
|  | Diet (CD vs. HFD) | *F*_(1,106.58)_ = 47.55 | <0.001 |
|  | Rostrocaudal level | *F*_(15,76.91)_ = 6.55 | <0.001 |
|  | Raphe subregion | *F*_(9,74.57)_ = 26.65 | <0.001 |
|  | Diet *Rostrocaudal level | *F*_(15,76.91)_ = 3.56 | 0.214 |
|  | Diet *Raphe subregion | *F*_(9,74.57)_ = 3.31 | 0.002 |
|  | Rostrocaudal level (Raphe subregion) | *F*_(53,63.95)_ = 2.56 | <0.001 |
|  | Treatment *Rostrocaudal level (Raphe subregion) | *F*_(53,63.95)_ = 1.31 | 0.147 |
|  |  |  |  |
| **Subregional Analyses (covariance structure)** | |  |  |
| **rDRD (First-Order Ante-Dependence)** | | |  |
|  | Treatment (CD vs. HFD) | *F*_(1,9.57)_ = 2.23 | 0.159 |
|  | Rostrocaudal level | *F*_(5,12.50)_ = 0.39 | 0.849 |
|  | Treatment *Rostrocaudal Level | *F*_(5,12.50)_ = 1.02 | 0.447 |
| **cDRD (Unstructured)** | | |  |
|  | Treatment (CD vs. HFD) | *F*_(1,11,77)_ = 28.89 | <0.001 |
|  | Rostrocaudal level | *F*_(4,10.46)_ = 5.33 | 0.0013 |
|  | Treatment *Rostrocaudal Level | *F*_(4,10.46)_ = 3.80 | 0.037 |
| **rDRV (First-Order Ante-Dependence)** | | |  |
|  | Treatment (CD vs. HFD) | *F*_(1,3.78)_ = 1.23 | 0.334 |
|  | Rostrocaudal level | *F*_(5,3.14)_ = 6.31 | 0.075 |
|  | Treatment *Rostrocaudal Level | *F*_(5,3.14)_ = 1.81 | 0.325 |
| **cDRV (First-Order Ante-Dependence)** | | |  |
|  | Treatment (CD vs. HFD) | *F*_(1,20.91)_ = 19.45 | <0.001 |
|  | Rostrocaudal level | *F*_(6,19.65)_ = 10.74 | <0.001 |
|  | Treatment *Rostrocaudal Level | *F*_(6,19.65)_ = 6.15 | <0.001 |
| **DRVL/VLPAG (First-Order Ante-Dependence)** | |  |  |
|  | Treatment (CD vs. HFD) | *F*_(1,20.71)_ = 3.27 | 0.085 |
|  | Rostrocaudal level | *F*_(8,15.66)_ = 4.63 | 0.005 |
|  | Treatment *Rostrocaudal Level | *F*_(8,15.66)_ = 1.49 | 0.238 |
| **DRC (Unstructured)** | |  |  |
|  | Treatment (CD vs. HFD) | *F*_(1,11.85)_ = 1.59 | 0.231 |
|  | Rostrocaudal level loop | *F*_(4,10.84)_ = 3.49 | 0.046 |
|  | Treatment *Rostrocaudal Level | *F*_(4,10.84)_ = 0.98 | 0.458 |
| **DRI (Unstructured)** | |  |  |
|  | Treatment (CD vs. HFD) | *F*_(1,12.31)_ = 0.70 | 0.417 |
|  | Rostrocaudal level | *F*_(4,10.27)_ = 1.07 | 0.421 |
|  | Treatment *Rostrocaudal Level | *F*_(4,10.27)_ = 1.92 | 0.182 |
| **MnR (Heterogeneous First-Order Autoregressive)** | | |  |
|  | Treatment (CD vs. HFD) | *F*_(1,16.51)_ = 11.39 | 0.004 |
|  | Rostrocaudal level | *F*_(15,27.31)_ = 6.18 | <0.001 |
|  | Treatment *Rostrocaudal Level | *F*_(15,27.31)_ = 3.72 | 0.001 |
| **PMRF (First-Order Ante-Dependence)** | | |  |
|  | Diet (CD vs HFD) | *F*_(1,9.23)_ = 3.78 | 0.083 |
|  | Rostrocaudal level | *F*_(8,6.83)_ = 0.44 | 0.865 |
|  | Treatment *Rostrocaudal Level | *F*_(8,6.83)_ = 1.51 | 0.304 |
| **B9 (First-Order Ante-Dependence)** | | |  |
|  | Treatment (CD vs. HFD) | *F*_(1,9.13)_ = 12.88 | 0.006 |
|  | Rostrocaudal level | *F*_(9,8.65)_ = 4.84 | 0.015 |
|  | Treatment *Rostrocaudal Level | *F*_(9,8.65)_ = 5.17 | 0.012 |

**5.3. Output for *in situ* hybridization for *slc6a4* mRNA expression**

**Table S6. Linear mixed model analysis for *slc6a4***

| **Model and source** | | **Test statistic** | ***p*-value** |
| --- | --- | --- | --- |
| **Overall Analysis (covariance structure)** | |  |  |
| **Entire dataset - First-order ante-dependence** | |  |  |
|  | Diet (CD vs. HFD) | *F*_(1,157.19)_ = 26.47 | <0.001 |
|  | Rostrocaudal level | *F*_(15,89.48)_ = 9.43 | <0.001 |
|  | Raphe subregion | *F*_(9,94.24)_ = 38.73 | <0.001 |
|  | Diet *Rostrocaudal level | *F*_(15,89.48)_ = 2.64 | 0.002 |
|  | Diet *Raphe subregion | *F*_(9,94.24)_ = 1.31 | 0.248 |
|  | Rostrocaudal level (Raphe subregion) | *F*_(53,82.36)_ = 2.83 | <0.001 |
|  | Treatment *Rostrocaudal level (Raphe subregion) | *F*_(53,82.36)_ = 1.49 | 0.050 |
|  |  |  |  |
| **Subregional Analyses (covariance structure)** | |  |  |
| **rDRD (First-Order Ante-Dependence)** | | |  |
|  | Treatment (CD vs. HFD) | *F*_(1,9.48)_ = 0.77 | 0.403 |
|  | Rostrocaudal level | *F*_(5,13.62)_ = 3.04 | 0.047 |
|  | Treatment *Rostrocaudal Level | *F*_(5,13.62)_ = 6.13 | 0.004 |
| **cDRD (Unstructured)** | | |  |
|  | Treatment (CD vs. HFD) | *F*_(1,12.16)_ = 0.95 | 0.349 |
|  | Rostrocaudal level | *F*_(4,10.01)_ = 0.66 | 0.635 |
|  | Treatment *Rostrocaudal Level | *F*_(4,10.01)_ = 2.27 | 0.133 |
| **rDRV (First-Order Ante-Dependence)** | | |  |
|  | Treatment (CD vs. HFD) | *F*_(1,12.01)_ = 3.79 | 0.075 |
|  | Rostrocaudal level | *F*_(5,11.91)_ = 8.85 | <0.001 |
|  | Treatment *Rostrocaudal Level | *F*_(5,11.91)_ = 3.80 | 0.027 |
| **cDRV (First-Order Ante-Dependence)** | | |  |
|  | Treatment (CD vs. HFD) | *F*_(1,13.23)_ = 5.79 | 0.031 |
|  | Rostrocaudal level | *F*_(6,12.93)_ = 13.17 | <0.001 |
|  | Treatment *Rostrocaudal Level | *F*_(6,12.93)_ = 0.74 | 0.628 |
| **DRVL/VLPAG (First-Order Ante-Dependence)** | | |  |
|  | Treatment (CD vs. HFD) | *F*_(1,16.42)_ = 1.73 | 0.206 |
|  | Rostrocaudal level | *F*_(8,14.48)_ = 13.88 | <0.001 |
|  | Treatment *Rostrocaudal Level | *F*_(8,14.48)_ = 0.97 | 0.492 |
| **DRC (Unstructured)** | | |  |
|  | Treatment (CD vs. HFD) | *F*_(1,12.93)_ = 4.28 | 0.059 |
|  | Rostrocaudal level | *F*_(4,8.37)_ = 12.67 | <0.001 |
|  | Treatment *Rostrocaudal Level | *F*_(4,8.37)_ = 1.20 | 0.380 |
| **DRI (Unstructured)** | | |  |
|  | Treatment (CD vs. HFD) | *F*_(1,8.34)_ = 10.74 | 0.011 |
|  | Rostrocaudal level | *F*_(4,6.38)_ = 5.98 | 0.024 |
|  | Treatment *Rostrocaudal Level | *F*_(4, 6.38)_ = 2.73 | 0.125 |
| **MnR (Heterogeneous First-Order Autoregressive)** | | |  |
|  | Treatment (CD vs. HFD) | *F*_(1,17.06)_ = 5.86 | 0.027 |
|  | Rostrocaudal level | *F*_(15,14.59)_ = 2.84 | 0.027 |
|  | Treatment *Rostrocaudal Level | *F*_(15,14.59)_ = 1.47 | 0.233 |
| **PMRF (First-Order Factor Analytic Constant Diagonal Offset)** | | |  |
|  | Diet (CD vs HFD) | *F*_(1,10.99)_ = 0.03 | 0.954 |
|  | Rostrocaudal level | *F*_(8,29.89)_ = 1.13 | 0.374 |
|  | Treatment *Rostrocaudal Level | *F*_(8,29.89)_ = 0.61 | 0.761 |
| **B9 (First-Order Ante-Dependence)** | | |  |
|  | Treatment (CD vs. HFD) | *F*_(1,11.90)_ = 0.05 | 0.830 |
|  | Rostrocaudal level | *F*_(9,14.51)_ = 5.67 | 0.002 |
|  | Treatment*Rostrocaudal Level | *F*_(9,14.51)_ = 4.77 | 0.004 |

**6. Supplementary statistical analysis for microbiome results.**

**6.1. Beta diversity results of weighted unifrac**

**Table S7. Weighted UniFrac pairwise PERMANOVA results for beta diversity**

| Group 1 | Group 2 | Sample size | pseudo-F | p-value | q-value |
| --- | --- | --- | --- | --- | --- |
| CD_M | CD_L | 41 | 4.250 | 0.003 | 0.003 |
| CD_M | CD_A | 60 | 5.202 | 0.002 | 0.002 |
| CD_M | HFD_M | 46 | 22.809 | 0.001 | 0.001 |
| CD_M | HFD_L | 41 | 29.884 | 0.001 | 0.001 |
| CD_M | HFD_A | 66 | 61.242 | 0.001 | 0.001 |
| CD_L | CD_A | 57 | 1.6963 | 0.133 | 0.139 |
| CD_L | HFD_M | 43 | 34.246 | 0.001 | 0.001 |
| CD_L | HFD_L | 38 | 48.159 | 0.001 | 0.001 |
| CD_L | HFD_A | 63 | 79.096 | 0.001 | 0.001 |
| CD_A | HFD_M | 62 | 37.499 | 0.001 | 0.001 |
| CD_A | HFD_L | 57 | 47.088 | 0.001 | 0.001 |
| CD_A | HFD_A | 82 | 90.580 | 0.001 | 0.001 |
| HFD_M | HFD_L | 43 | 1.737 | 0.139 | 0.139 |
| HFD_M | HFD_A | 68 | 11.203 | 0.001 | 0.001 |
| HFD_L | HFD_A | 63 | 6.895 | 0.001 | 0.001 |

**6.2. Beta diversity results of unweighted unifrac**

**Table S8. Unweighted UniFrac pairwise PERMANOVA results for beta diversity**

| Group 1 | Group 2 | Sample size | pseudo-F | p-value | q-value |
| --- | --- | --- | --- | --- | --- |
| CD_M | CD_L | 41 | 1.859 | 0.003 | 0.003 |
| CD_M | CD_A | 60 | 4.888 | 0.001 | 0.001 |
| CD_M | HFD_M | 46 | 11.260 | 0.001 | 0.001 |
| CD_M | HFD_L | 41 | 12.851 | 0.001 | 0.001 |
| CD_M | HFD_A | 66 | 23.163 | 0.001 | 0.001 |
| CD_L | CD_A | 57 | 2.992 | 0.001 | 0.001 |
| CD_L | HFD_M | 43 | 12.308 | 0.001 | 0.001 |
| CD_L | HFD_L | 38 | 14.235 | 0.001 | 0.001 |
| CD_L | HFD_A | 63 | 22.687 | 0.001 | 0.001 |
| CD_A | HFD_M | 62 | 17.864 | 0.001 | 0.001 |
| CD_A | HFD_L | 57 | 20.578 | 0.001 | 0.001 |
| CD_A | HFD_A | 82 | 31.714 | 0.001 | 0.001 |
| HFD_M | HFD_L | 43 | 2.684 | 0.005 | 0.005 |
| HFD_M | HFD_A | 68 | 8.166 | 0.001 | 0.001 |
| HFD_L | HFD_A | 63 | 6.072 | 0.001 | 0.001 |

**6.3. Results for relative abundance analysis using ANCOM for phylum, class, order, family, genus, and species.**

**Table S9. Results of phylum taxonomic ANCOM-II analysis**

| **Taxa: "k__Bacteria;p__Bacteroidetes"** | | | | | |
| --- | --- | --- | --- | --- | --- |
| Kruskal-Wallis *p-*value = 5.958e-05  Wilcoxon rank sum test (pairwise comparisons), value adjustment method: BH | | | | | |
|  | CD-M | CD-L | CD-A | HFD-M | HFD-L |
| CD-L | 0.87874 | - | - | - | - |
| CD-A | 0.08771 | 0.01388* | - | - | - |
| HFD-M | 0.40395 | 0.21071 | 0.48972 | - | - |
| HFD-L | 0.36699 | 0.13746 | 0.75077 | 0.77451 | - |
| HFD-A | 0.00296 | 0.00022 | 0.03604* | 0.03604* | 0.08771 |
| **Taxa: "k__Bacteria;p__Deferribacteres"** | | | | | |
| Kruskal-wallis *p-*value = 0.03179  Wilcoxon rank sum test (pairwise comparisons), value adjustment method: BH | | | | | |
|  | CD-M | CD-L | CD-A | HFD-M | HFD-L |
| CD-L | 0.398 | - | - | - | - |
| CD-A | 0.784 | 0.398 | - | - | - |
| HFD-M | 0.885 | 0.353 | 0.675 | - | - |
| HFD-L | 0.539 | 0.144 | 0.353 | 0.671 | - |
| HFD-A | 0.388 | 0.015 | 0.144 | 0.398 | 0.952 |
| **Taxa: "k__Bacteria;p__Tenericutes"** | | | | | |
| Kruskal-Wallis *p-*value = 0.01563  Wilcoxon rank sum test (pairwise comparisons), value adjustment method: BH | | | | | |
|  | CD-M | CD-L | CD-A | HFD-M | HFD-L |
| CD-L | 0.678 | - | - | - | - |
| CD-A | 0.678 | 0.517 | - | - | - |
| HFD-M | 0.094 | 0.083 | 0.094 | - | - |
| HFD-L | 0.562 | 0.562 | 0.678 | 0.562 | - |
| HFD-A | 0.083 | 0.045 | 0.083 | 0.562 | 0.925 |

*Differences detected by pairwise comparisons using Wilcoxon ran sum test with continuity correction.

**Table S10. Results of class taxonomic ANCOM analysis**

| **Taxa: "k__Bacteria;p__Bacteroidetes;c__Bacteroidia"** | | | | | |
| --- | --- | --- | --- | --- | --- |
| Kruskal-Wallis *p-*value = 5.958e-05  Wilcoxon rank sum test (pairwise comparisons), value adjustment method: BH | | | | | |
|  | CD-M | CD-L | CD-A | HFD-M | HFD-L |
| CD-L | 0.878 | - | - | - | - |
| CD-A | 0.087 | 0.013* | - | - | - |
| HFD-M | 0.403 | 0.210 | 0.489 | - | - |
| HFD-L | 0.366 | 0.137 | 0.750 | 0.774 | - |
| HFD-A | 0.002 | <0.001 | 0.036* | 0.036* | 0.087 |
| **Taxa: "k__Bacteria;p__Deferribacteres;c__Deferribacteres"** | | | | | |
| Kruskal-Wallis *p-*value = 0.03179  Wilcoxon rank sum test (pairwise comparisons), value adjustment method: BH | | | | | |
|  | CD-M | CD-L | CD-A | HFD-M | HFD-L |
| CD-L | 0.398 | - | - | - | - |
| CD-A | 0.784 | 0.398 | - | - | - |
| HFD-M | 0.885 | 0.353 | 0.675 | - | - |
| HFD-L | 0.539 | 0.144 | 0.353 | 0.671 | - |
| HFD-A | 0.388 | 0.015 | 0.144 | 0.398 | 0.952 |
| **Taxa: "k__Bacteria;p__Firmicutes;c__Bacilli"** | | | | | |
| Kruskal-Wallis *p-*value = 2.749e-06  Wilcoxon rank sum test (pairwise comparisons), value adjustment method: BH | | | | | |
|  | CD-M | CD-L | CD-A | HFD-M | HFD-L |
| CD-L | 0.605 | - | - | - | - |
| CD-A | 0.094 | 0.007* | - | - | - |
| HFD-M | 0.189 | 0.085 | 0.751 | - | - |
| HFD-L | 0.543 | 0.170 | 0.472 | 0.543 | - |
| HFD-A | <0.001 | <0.001 | 0.001* | 0.081 | 0.007* |

*Differences detected by pairwise comparisons using Wilcoxon ran sum test with continuity correction.

**Table S11. Results of order taxonomic ANCOM analysis**

| **Taxa: "k__Bacteria;p__Bacteroidetes;c__Bacteroidia;o__Bacteroidales"** | | | | | |
| --- | --- | --- | --- | --- | --- |
| Kruskal-Wallis *p-*value = 5.958e-05  Wilcoxon rank sum test (pairwise comparisons), value adjustment method: BH | | | | | |
|  | CD-M | CD-L | CD-A | HFD-M | HFD-L |
| CD-L | 0.878 | - | - | - | - |
| CD-A | 0.087 | 0.013* | - | - | - |
| HFD-M | 0.403 | 0.210 | 0.489 | - | - |
| HFD-L | 0.366 | 0.137 | 0.750 | 0.774 | - |
| HFD-A | 0.002 | <0.001 | 0.036* | 0.036* | 0.087 |
| **Taxa: "k__Bacteria;p__Firmicutes;c__Bacilli;o__Lactobacillales"** | | | | | |
| Kruskal-Wallis *p-*value = 1.388e-06  Wilcoxon rank sum test (pairwise comparisons), value adjustment method: BH | | | | | |
|  | CD-M | CD-L | CD-A | HFD-M | HFD-L |
| CD-L | 0.641 | - | - | - | - |
| CD-A | 0.097 | 0.006* | - | - | - |
| HFD-M | 0.189 | 0.094 | 0.763 | - | - |
| HFD-L | 0.494 | 0.129 | 0.484 | 0.641 | - |
| HFD-A | <0.001 | <0.001 | <0.001* | 0.041* | 0.006* |
| **Taxa:"k__Bacteria;p__Proteobacteria;c__Gammaproteobacteria;o__Enterobacte-riales"** | | | | | |
| Kruskal-Wallis *p-*value = 1.607e-07  Wilcoxon rank sum test (pairwise comparisons), value adjustment method: BH | | | | | |
|  | CD-M | CD-L | CD-A | HFD-M | HFD-L |
| CD-L | 0.004 | - | - | - | - |
| CD-A | 0.001* | 0.880 | - | - | - |
| HFD-M | 0.982 | 0.003 | <0.001 | - | - |
| HFD-L | 0.751 | 0.004* | <0.001 | 0.751 | - |
| HFD-A | 0.017 | 0.003 | 0.003* | 0.003* | 0.004* |

*Differences detected by pairwise comparisons using Wilcoxon ran sum test with continuity correction.

**Table S12. Results of family taxonomic ANCOM analysis**

| **Taxa:"k__Bacteria;p__Actinobacteria;c__Actinobacteria;o__Bifidobacteriales;f__Bifidobacteriaceae"** | | | | | |
| --- | --- | --- | --- | --- | --- |
| Kruskal-Wallis *p-*value = 0.0002731  Wilcoxon rank sum test (pairwise comparisons), value adjustment method: BH | | | | | |
|  | CD-M | CD-L | CD-A | HFD-M | HFD-L |
| CD-L | 0.934 | - | - | - | - |
| CD-A | 0.008* | 0.008* | - | - | - |
| HFD-M | 0.610 | 0.621 | 0.061 | - | - |
| HFD-L | 0.621 | 0.610 | 0.008 | 0.360 | - |
| HFD-A | 0.011 | 0.024 | 0.879 | 0.097 | 0.008* |
| **Taxa:"k__Bacteria;p__Bacteroidetes;c__Bacteroidia;o__Bacteroidales;f__Prevotellaceae"** | | | | | |
| Kruskal-Wallis *p-*value = 6.203e-07  Wilcoxon rank sum test (pairwise comparisons), value adjustment method: BH | | | | | |
|  | CD-M | CD-L | CD-A | HFD-M | HFD-L |
| CD-L | 0.572 | - | - | - | - |
| CD-A | 0.116 | 0.004 | - | - | - |
| HFD-M | 0.118 | 0.116 | 0.566 | - | - |
| HFD-L | 0.118 | 0.118 | 0.118 | 0.720 | - |
| HFD-A | 2.1e-05 | 1.2e-05 | 2.3e-05* | 0.096 | 0.293 |
| **Taxa**:**"k__Bacteria;p__Bacteroidetes;c__Bacteroidia;o__Bacteroidales;f__[Paraprevotellaceae]"** | | | | | |
| Kruskal-Wallis *p-*value = 0.001112  Wilcoxon rank sum test (pairwise comparisons), value adjustment method: BH | | | | | |
|  | CD-M | CD-L | CD-A | HFD-M | HFD-L |
| CD-L | 0.934 | - | - | - | - |
| CD-A | 0.216 | 0.128 | - | - | - |
| HFD-M | 0.299 | 0.144 | 0.854 | - | - |
| HFD-L | 0.046 | 0.034 | 0.128 | 0.295 | - |
| HFD-A | 0.033 | 0.005 | 0.046* | 0.251 | 0.934 |
| **Taxa:"k__Bacteria;p__Firmicutes;c__Clostridia;o__Clostridiales;f__Clostridiaceae"** | | | | | |
| Kruskal-Wallis *p-*value = 8.329e-05  Wilcoxon rank sum test (pairwise comparisons), value adjustment method: BH | | | | | |
|  | CD-M | CD-L | CD-A | HFD-M | HFD-L |
| CD-L | 0.296 | - | - | - | - |
| CD-A | 0.028* | 0.355 | - | - | - |
| HFD-M | <0.001 | 0.061 | 0.198 | - | - |
| HFD-L | <0.001 | 0.061 | 0.061 | 0.457 | - |
| HFD-A | <0.001 | 0.065 | 0.556 | 0.198 | 0.061 |
| **Taxa:"k__Bacteria;p__Proteobacteria;c__Betaproteobacteria;o__Burkholderiales;f__Alcaligenaceae"** | | | | | |
| Kruskal-Wallis *p-*value = 0.003392  Wilcoxon rank sum test (pairwise comparisons), value adjustment method: BH | | | | | |
|  | CD-M | CD-L | CD-A | HFD-M | HFD-L |
| CD-L | 0.882 | - | - | - | - |
| CD-A | 0.448 | 0.679 | - | - | - |
| HFD-M | 0.882 | 0.882 | 0.448 | - | - |
| HFD-L | 0.679 | 0.852 | 0.927 | 0.679 | - |
| HFD-A | 0.011 | 0.017 | 0.064 | 0.006* | 0.448 |

*Differences detected by pairwise comparisons using Wilcoxon ran sum test with continuity correction.

**Table S13. Results of genus taxonomic ANCOM analysis**

| **Taxa:"k__Bacteria;p__Bacteroidetes;c__Bacteroidia;o__Bacteroidales;f__Prevotellaceae;g__Prevotella"** | | | | | |
| --- | --- | --- | --- | --- | --- |
| Kruskal-Wallis *p-*value = 6.203e-07  Wilcoxon rank sum test (pairwise comparisons), value adjustment method: BH | | | | | |
|  | CD-M | CD-L | CD-A | HFD-M | HFD-L |
| CD-L | 0.572 | - | - | - | - |
| CD-A | 0.116 | 0.004* | - | - | - |
| HFD-M | 0.118 | 0.116 | 0.566 | - | - |
| HFD-L | 0.118 | 0.118 | 0.118 | 0.720 | - |
| HFD-A | 2.1e-05 | 1.2e-05 | 2.3e-05* | 0.096 | 0.293 |
| **Taxa:"k__Bacteria;p__Bacteroidetes;c__Bacteroidia;o__Bacteroidales;f__[Paraprevotellaceae];g__[Prevotella]"** | | | | | |
| Kruskal-Wallis *p-*value = 0.0004782  Wilcoxon rank sum test (pairwise comparisons), value adjustment method: BH | | | | | |
|  | CD-M | CD-L | CD-A | HFD-M | HFD-L |
| CD-L | 0.503 | - | - | - | - |
| CD-A | 0.449 | 0.051 | - | - | - |
| HFD-M | 0.363 | 0.039 | 0.386 | - | - |
| HFD-L | 0.045 | 0.014* | 0.045 | 0.386 | - |
| HFD-A | 0.039 | 0.002 | 0.039* | 0.410 | 0.479 |
| **Taxa:"k__Bacteria;p__Firmicutes;c__Clostridia;o__Clostridiales;f__Clostridiaceae;g__"** | | | | | |
| Kruskal-Wallis *p-*value = 3.211e-13  Wilcoxon rank sum test (pairwise comparisons), value adjustment method: BH | | | | | |
|  | CD-M | CD-L | CD-A | HFD-M | HFD-L |
| CD-L | 1.000 | - | - | - | - |
| CD-A | 0.002 | 0.002* | - | - | - |
| HFD-M | 1.2e-07* | 1.2e-07 | 0.039 | - | - |
| HFD-L | 2.5e-07 | 2.5e-07* | 0.020 | 0.427 | - |
| HFD-A | 1.2e-07 | 1.2e-07 | 0.118 | 0.207 | 0.071 |
| **Taxa:"k__Bacteria;p__Firmicutes;c__Clostridia;o__Clostridiales;f__Lachnospiraceae;g__"** | | | | | |
| Kruskal-Wallis *p-*value = 0.0008341  Wilcoxon rank sum test (pairwise comparisons), value adjustment method: BH | | | | | |
|  | CD-M | CD-L | CD-A | HFD-M | HFD-L |
| CD-L | 0.871 | - | - | - | - |
| CD-A | 0.622 | 0.907 | - | - | - |
| HFD-M | 0.033* | 0.071 | 0.082 | - | - |
| HFD-L | 0.033 | 0.067 | 0.024 | 0.082 | - |
| HFD-A | 0.033 | 0.082 | 0.200 | 0.183 | 0.003* |
| **Taxa:"k__Bacteria;p__Firmicutes;c__Clostridia;o__Clostridiales;f__Ruminococcaceae;g__Oscillospira"** | | | | | |
| Kruskal-Wallis *p-*value = 0.003418  Wilcoxon rank sum test (pairwise comparisons), value adjustment method: BH | | | | | |
|  | CD-M | CD-L | CD-A | HFD-M | HFD-L |
| CD-L | 0.946 | - | - | - | - |
| CD-A | 0.946 | 0.946 | - | - | - |
| HFD-M | 0.047* | 0.047 | 0.047 | - | - |
| HFD-L | 0.048 | 0.047* | 0.047 | 0.887 | - |
| HFD-A | 0.047 | 0.047 | 0.047* | 0.946 | 0.758 |
| **Taxa:"k__Bacteria;p__Firmicutes;c__Clostridia;o__Clostridiales;f__Veillonellaceae;g__"** | | | | | |
| Kruskal-Wallis *p-*value = 5.632e-11  Wilcoxon rank sum test (pairwise comparisons), value adjustment method: BH | | | | | |
|  | CD-M | CD-L | CD-A | HFD-M | HFD-L |
| CD-L | 0.943 | - | - | - | - |
| CD-A | 0.521 | 0.521 | - | - | - |
| HFD-M | <0.001* | <0.001 | <0.001 | - | - |
| HFD-L | 0.002 | 0.002* | <0.001 | 0.780 | - |
| HFD-A | 1.5e-06 | 1.5e-06 | 7.8e-07* | 0.679 | 0.480 |
| **Taxa:"k__Bacteria;p__Firmicutes;c__Clostridia;o__Clostridiales;f__Veillonellaceae;g__Anaerovibrio"** | | | | | |
| Kruskal-Wallis *p-*value = 6.562e-13  Wilcoxon rank sum test (pairwise comparisons), value adjustment method: BH | | | | | |
|  | CD-M | CD-L | CD-A | HFD-M | HFD-L |
| CD-L | 0.876 | - | - | - | - |
| CD-A | 0.581 | 0.619 | - | - | - |
| HFD-M | 0.003 | 0.006 | 0.003 | - | - |
| HFD-L | 4.8e-05 | 9.7e-05 | 4.1e-05 | 0.154 | - |
| HFD-A | 7.1e-09 | 2.3e-08 | 7.1e-09 | 0.025* | - |
| **Taxa:"k__Bacteria;p__Proteobacteria;c__Epsilonproteobacteria;o__Campylobacterales;f__Helicobacteraceae;g__"** | | | | | |
| Kruskal-Wallis *p-*value = 0.01934  Wilcoxon rank sum test (pairwise comparisons), value adjustment method: BH | | | | | |
|  | CD-M | CD-L | CD-A | HFD-M | HFD-L |
| CD-L | 0.360 | - | - | - | - |
| CD-A | 0.110 | 0.730 | - | - | - |
| HFD-M | 0.950 | 0.350 | 0.110 | - | - |
| HFD-L | 0.820 | 0.270 | 0.110 | 0.820 | - |
| HFD-A | 0.730 | 0.490 | 0.110 | 0.700 | 0.490 |
| **Taxa:"k__Bacteria;p__Tenericutes;c__Mollicutes;o__Mycoplasmatales;f__Mycoplasmataceae;g__Mycoplasma"** | | | | | |
| Kruskal-Wallis *p-*value = 0.01563  Wilcoxon rank sum test (pairwise comparisons), value adjustment method: BH | | | | | |
|  | CD-M | CD-L | CD-A | HFD-M | HFD-L |
| CD-L | 0.678 | - | - | - | - |
| CD-A | 0.678 | 0.517 | - | - | - |
| HFD-M | 0.094 | 0.083 | 0.094 | - | - |
| HFD-L | 0.562 | 0.562 | 0.678 | 0.562 | - |
| HFD-A | 0.083 | 0.045 | 0.083 | 0.562 | 0.925 |

*Differences detected by pairwise comparisons using Wilcoxon ran sum test with continuity correction.

**Table S14. Results of species taxonomic ANCOM analysis**

| **Taxa:"k__Bacteria;p__Bacteroidetes;c__Bacteroidia;o__Bacteroidales;f__Prevotellaceae;g__Prevotella;s__"** | | | | | |
| --- | --- | --- | --- | --- | --- |
| Kruskal-Wallis *p-*value = 0.00000001803  Wilcoxon rank sum test (pairwise comparisons), value adjustment method: BH | | | | | |
|  | CD-M | CD-L | CD-A | HFD-M | HFD-L |
| CD-L | 0.4722 | - | - | - | - |
| CD-A | 0.0191* | 0.0019* | - | - | - |
| HFD-M | 0.2006 | 0.0917 | 0.4270 | - | - |
| HFD-L | 0.0177 | 0.0147* | 0.0093 | 0.4722 | - |
| HFD-A | 0.0000055 | 0.0000029 | 0.0000029* | 0.0446* | 0.4722 |
| **Taxa:"k__Bacteria;p__Bacteroidetes;c__Bacteroidia;o__Bacteroidales;f__Prevotellaceae;g__Prevotella;s__copri"** | | | | | |
| Kruskal-Wallis *p-*value = 0.00000000000000022  Wilcoxon rank sum test (pairwise comparisons), value adjustment method: BH | | | | | |
|  | CD-M | CD-L | CD-A | HFD-M | HFD-L |
| CD-L | 0.13209 | - | - | - | - |
| CD-A | 0.19233 | < 0.0001 | - | - | - |
| HFD-M | <0.0001* | < 0.0001 | < 0.0001 | - | - |
| HFD-L | < 0.0001 | < 0.0001* | < 0.0001 | 0.14414 | - |
| HFD-A | < 0.0001 | < 0.0001 | < 0.0001* | 0.15575 | 0.80264 |
| **Taxa:"k__Bacteria;p__Bacteroidetes;c__Bacteroidia;o__Bacteroidales;f__Prevotellaceae;g__Prevotella;s__stercorea"** | | | | | |
| Kruskal-Wallis *p-*value = 0.00000000000000022  Wilcoxon rank sum test (pairwise comparisons), value adjustment method: BH | | | | | |
|  | CD-M | CD-L | CD-A | HFD-M | HFD-L |
| CD-L | 0.6465 | - | - | - | - |
| CD-A | 0.6299 | 0.1299 | - | - | - |
| HFD-M | 0.0072* | 0.0011 | 0.0011 | - | - |
| HFD-L | < 0.0001 | < 0.0001* | < 0.0001 | 0.0496* | - |
| HFD-A | < 0.0001 | < 0.0001 | < 0.0001* | 0.0023* | - |
| **Taxa:"k__Bacteria;p__Bacteroidetes;c__Bacteroidia;o__Bacteroidales;f__[Paraprevotellaceae];g__[Prevotella];s__"** | | | | | |
| Kruskal-Wallis *p-*value = 0.0004062  Wilcoxon rank sum test (pairwise comparisons), value adjustment method: BH | | | | | |
|  | CD-M | CD-L | CD-A | HFD-M | HFD-L |
| CD-L | 0.3925 | - | - | - | - |
| CD-A | 0.3925 | 0.0282 | - | - | - |
| HFD-M | 0.3633 | 0.0396 | 0.3925 | - | - |
| HFD-L | 0.0437 | 0.0145 | 0.0603 | 0.3925 | - |
| HFD-A | 0.0396 | 0.0018 | 0.0437 | 0.3925 | 0.4475 |
| **Taxa:"k__Bacteria;p__Bacteroidetes;c__Bacteroidia;o__Bacteroidales;f__p-2534-18B5;g__;s__"** | | | | | |
| Kruskal-Wallis *p-*value = 0.04366  Wilcoxon rank sum test (pairwise comparisons), value adjustment method: BH | | | | | |
|  | CD-M | CD-L | CD-A | HFD-M | HFD-L |
| CD-L | 0.785 | - | - | - | - |
| CD-A | 0.741 | 0.875 | - | - | - |
| HFD-M | 0.774 | 0.601 | 0.376 | - | - |
| HFD-L | 0.376 | 0.283 | 0.056 | 0.498 | - |
| HFD-A | 0.376 | 0.325 | 0.056 | 0.652 | 0.770 |
| **Taxa:"k__Bacteria;p__Firmicutes;c__Bacilli;o__Lactobacillales;f__Lactobacillaceae;g__Lactobacillus;s__helveticus"** | | | | | |
| Kruskal-Wallis *p-*value = 0.0000000006741  Wilcoxon rank sum test (pairwise comparisons), value adjustment method: BH | | | | | |
|  | CD-M | CD-L | CD-A | HFD-M | HFD-L |
| CD-L | 0.18347 | - | - | - | - |
| CD-A | 0.00994* | <0.0001* | - | - | - |
| HFD-M | 0.15113 | 0.01985 | 0.42693 | - | - |
| HFD-L | 0.52181 | 0.13557 | 0.11858 | 0.42693 | - |
| HFD-A | <0.0001 | <0.0001 | <0.0001* | <0.0001* | <0.0001* |
| **Taxa:"k__Bacteria;p__Firmicutes;c__Clostridia;o__Clostridiales;f__Clostridiaceae;g__;s__"** | | | | | |
| Kruskal-Wallis *p-*value = 0.000000000001296  Wilcoxon rank sum test (pairwise comparisons), value adjustment method: BH | | | | | |
|  | CD-M | CD-L | CD-A | HFD-M | HFD-L |
| CD-L | 0.9442 | - | - | - | - |
| CD-A | 0.0035* | 0.0074* | - | - | - |
| HFD-M | <0.0001* | <0.0001 | 0.0130 | - | - |
| HFD-L | <0.0001 | <0.0001* | 0.0074 | 0.4273 | - |
| HFD-A | <0.0001 | <0.0001 | 0.0438* | 0.2075 | 0.0655 |
| **Taxa:"k__Bacteria;p__Firmicutes;c__Clostridia;o__Clostridiales;f__Lachnospiraceae;g__Blautia;s__producta"** | | | | | |
| Kruskal-Wallis *p-*value = 0.00000000000000022  Wilcoxon rank sum test (pairwise comparisons), value adjustment method: BH | | | | | |
|  | CD-M | CD-L | CD-A | HFD-M | HFD-L |
| CD-L | 0.44 | - | - | - | - |
| CD-A | 0.67 | 0.28 | - | - | - |
| HFD-M | <0.0001* | <0.0001 | <0.0001 | - | - |
| HFD-L | <0.0001 | <0.0001* | <0.0001 | 0.54 | - |
| HFD-A | <0.0001 | <0.0001 | <0.0001* | 0.11 | 0.10 |
| **Taxa:"k__Bacteria;p__Firmicutes;c__Clostridia;o__Clostridiales;f__Lachnospiraceae;g__Dorea;s__"** | | | | | |
| Kruskal-Wallis *p-*value = 0.114  Wilcoxon rank sum test (pairwise comparisons), value adjustment method: BH | | | | | |
|  | CD-M | CD-L | CD-A | HFD-M | HFD-L |
| CD-L | 0.45 | - | - | - | - |
| CD-A | 0.94 | 0.35 | - | - | - |
| HFD-M | 0.35 | 0.67 | 0.35 | - | - |
| HFD-L | 0.35 | 0.90 | 0.31 | 0.45 | - |
| HFD-A | 0.37 | 0.37 | 0.67 | 0.35 | 0.22 |
| **Taxa:"k__Bacteria;p__Firmicutes;c__Clostridia;o__Clostridiales;f__Ruminococcaceae;g__Oscillospira;s__"** | | | | | |
| Kruskal-Wallis *p-*value = 0.002705  Wilcoxon rank sum test (pairwise comparisons), value adjustment method: BH | | | | | |
|  | CD-M | CD-L | CD-A | HFD-M | HFD-L |
| CD-L | 1.000 | - | - | - | - |
| CD-A | 0.995 | 0.995 | - | - | - |
| HFD-M | 0.054 | 0.054 | 0.054 | - | - |
| HFD-L | 0.054 | 0.068 | 0.054 | 0.887 | - |
| HFD-A | 0.054 | 0.054 | 0.054 | 0.995 | 0.758 |
| **Taxa:"k__Bacteria;p__Firmicutes;c__Clostridia;o__Clostridiales;f__Veillonellaceae;g__;s__"** | | | | | |
| Kruskal-Wallis *p-*value = 0.00000000005198  Wilcoxon rank sum test (pairwise comparisons), value adjustment method: BH | | | | | |
|  | CD-M | CD-L | CD-A | HFD-M | HFD-L |
| CD-L | 1.00000 | - | - | - | - |
| CD-A | 0.35168 | 0.49071 | - | - | - |
| HFD-M | <0.0001* | 0.00050 | 0.00025 | - | - |
| HFD-L | 0.00234 | 0.00234* | 0.00106 | 0.78077 | - |
| HFD-A | <0.0001 | <0.0001 | <0.0001* | 0.67924 | 0.43640 |
| **Taxa:"k__Bacteria;p__Firmicutes;c__Clostridia;o__Clostridiales;f__Veillonellaceae;g__Anaerovibrio;s__"** | | | | | |
| Kruskal-Wallis *p-*value = 0.0000000000006597  Wilcoxon rank sum test (pairwise comparisons), value adjustment method: BH | | | | | |
|  | CD-M | CD-L | CD-A | HFD-M | HFD-L |
| CD-L | 0.88279 | - | - | - | - |
| CD-A | 0.55782 | 0.76444 | - | - | - |
| HFD-M | 0.00264* | 0.01454 | 0.00145 | - | - |
| HFD-L | <0.0001 | 0.00035* | <0.0001 | 0.15410 | - |
| HFD-A | <0.0001 | <0.0001 | <0.0001* | 0.02543* | - |
| **Taxa:"k__Bacteria;p__Proteobacteria;c__Epsilonproteobacteria;o__Campylobacterales;f__Helicobacteraceae;g__;s__"** | | | | | |
| Kruskal-Wallis *p-*value = 0.02646  Wilcoxon rank sum test (pairwise comparisons), value adjustment method: BH | | | | | |
|  | CD-M | CD-L | CD-A | HFD-M | HFD-L |
| CD-L | 0.23 | - | - | - | - |
| CD-A | 0.17 | 0.99 | - | - | - |
| HFD-M | 0.99 | 0.22 | 0.17 | - | - |
| HFD-L | 0.88 | 0.17 | 0.17 | 0.88 | - |
| HFD-A | 0.74 | 0.28 | 0.17 | 0.70 | 0.49 |
| **Taxa**:**"k__Bacteria;p__Tenericutes;c__Mollicutes;o__Mycoplasmatales;f__Mycoplasmataceae;g__Mycoplasma;s__"** | | | | | |
| Kruskal-Wallis *p-*value = 0.019  Wilcoxon rank sum test (pairwise comparisons), value adjustment method: BH | | | | | |
|  | CD-M | CD-L | CD-A | HFD-M | HFD-L |
| CD-L | 0.652 | - | - | - | - |
| CD-A | 0.735 | 0.562 | - | - | - |
| HFD-M | 0.094 | 0.067 | 0.067 | - | - |
| HFD-L | 0.562 | 0.562 | 0.652 | 0.562 | - |
| HFD-A | 0.067 | 0.067 | 0.067 | 0.562 | 0.925 |

*Differences detected by pairwise comparisons using Wilcoxon ran sum test with continuity correction.

**References**

Day, H. E., and H. Akil. 1996. 'Differential pattern of c-fos mRNA in rat brain following central and systemic administration of interleukin-1-beta: implications for mechanism of action', *Neuroendocrinology*, 63: 207-18.

Lieb, M. W., M. Weidner, M. R. Arnold, K. M. Loupy, K. T. Nguyen, J. E. Hassell, Jr., K. S. Schnabel, R. Kern, H. E. W. Day, K. P. Lesch, J. Waider, and C. A. Lowry. 2019. 'Effects of maternal separation on serotonergic systems in the dorsal and median raphe nuclei of adult male Tph2-deficient mice', *Behav Brain Res*, 373: 112086.
